# Synthesis of a library of tricyclic azepinoisoindolinones

**DOI:** 10.3762/bjoc.8.120

**Published:** 2012-07-13

**Authors:** Bettina Miller, Shuli Mao, Kara M George Rosenker, Joshua G Pierce, Peter Wipf

**Affiliations:** 1 Center for Chemical Methodologies & Library Development (CMLD), Department of Chemistry, University of Pittsburgh, 219 Parkman Avenue, Pittsburgh, PA 15260, USA

**Keywords:** chemical diversity, epoxide aminolysis, hydrozirconation, isoindolinones, metathesis, *N*-acyliminium ion

## Abstract

Hydrozirconation of 1-hexyne, the addition to in situ prepared *N*-acyliminium species, and ring-closing metathesis (RCM) were key steps in the preparation of a tricyclic isoindolinone scaffold. An unusual alkene isomerization process during the RCM was identified and studied in some detail. Chemical diversification for library synthesis was achieved by a subsequent alkene epoxidation and zinc-mediated aminolysis reaction. The resulting library products provided selective hits among a large number of high-throughput screens reported in PubChem, thus illustrating the utility of the novel scaffold.

## Introduction

**Figure 1:**
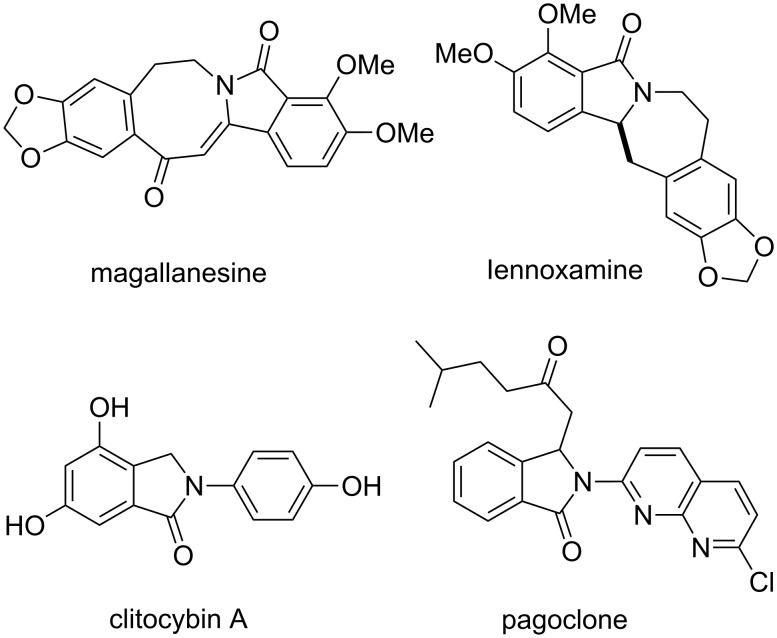
Representative isoindolinone natural products and pharmaceuticals.

Isoindolinones represent a common scaffold seen in naturally occurring compounds such as magallanesine [1], lennoxamine [2] and clitocybin A [3], or drug candidates such as pagoclone [4] (Figure 1). These heterocycles have demonstrated a variety of pharmacological activities, including anti-inflammatory [5], antihypertensive [6] and vasodilatory [7], antipsychotic [8–9], and anticancer effects [10]. Due to the broad biological properties and the general utility of isoindolinones in the preparation of other synthetic building blocks, a variety of approaches for the preparation of these heterocycles have been explored [11–18]. Previously, we reported on the addition of organometallic reagents to in situ generated *N*-acyliminium ions [19]. This methodology applies to a variety of commercially available or easily prepared starting materials and creates many opportunities for further functionalization and chemical library synthesis. For example, a ring-closing metathesis of the alkene addition product affords structurally novel tricyclic isoindolinones with a newly formed seven-membered ring [19]. We have now developed this concept further toward a library synthesis of functionalized azepino-isoindolinone derivatives.

## Results and Discussion

**Scheme 1:**
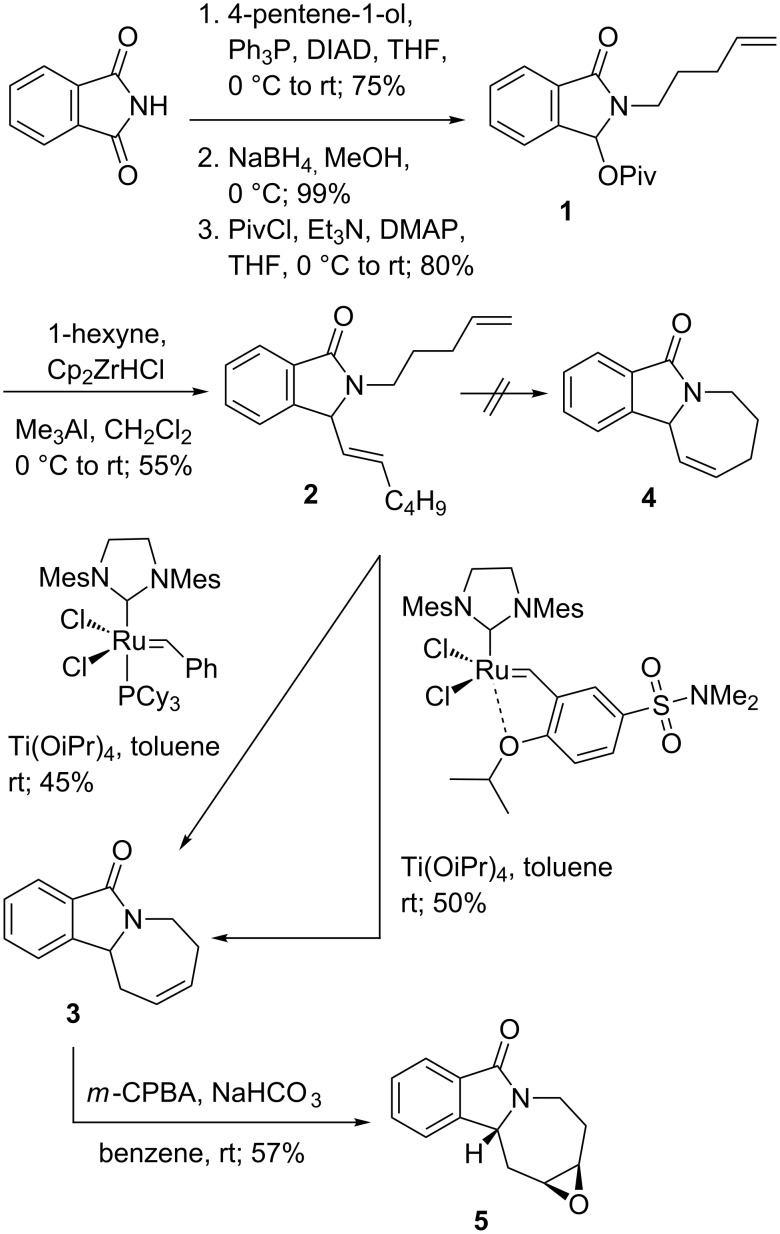
Formation of isomerized azepinoisoindoline **3** and oxirane **5**.

N-Alkylation of phthalimide with 4-penten-1-ol under Mitsunobu conditions, followed by NaBH_4_ reduction and pivaloate protection of the intermediate hemiaminal, provided alkene **1** in 59% overall yield (Scheme 1). After hydrozirconation of 1-hexyne with zirconocene hydrochloride [20–23], addition of trimethylaluminium activated the in situ generated alkenylzirconocene and allowed the displacement of the pivaloate on **1** in 55% yield to afford diene **2** [19,24].

**Figure 2:**
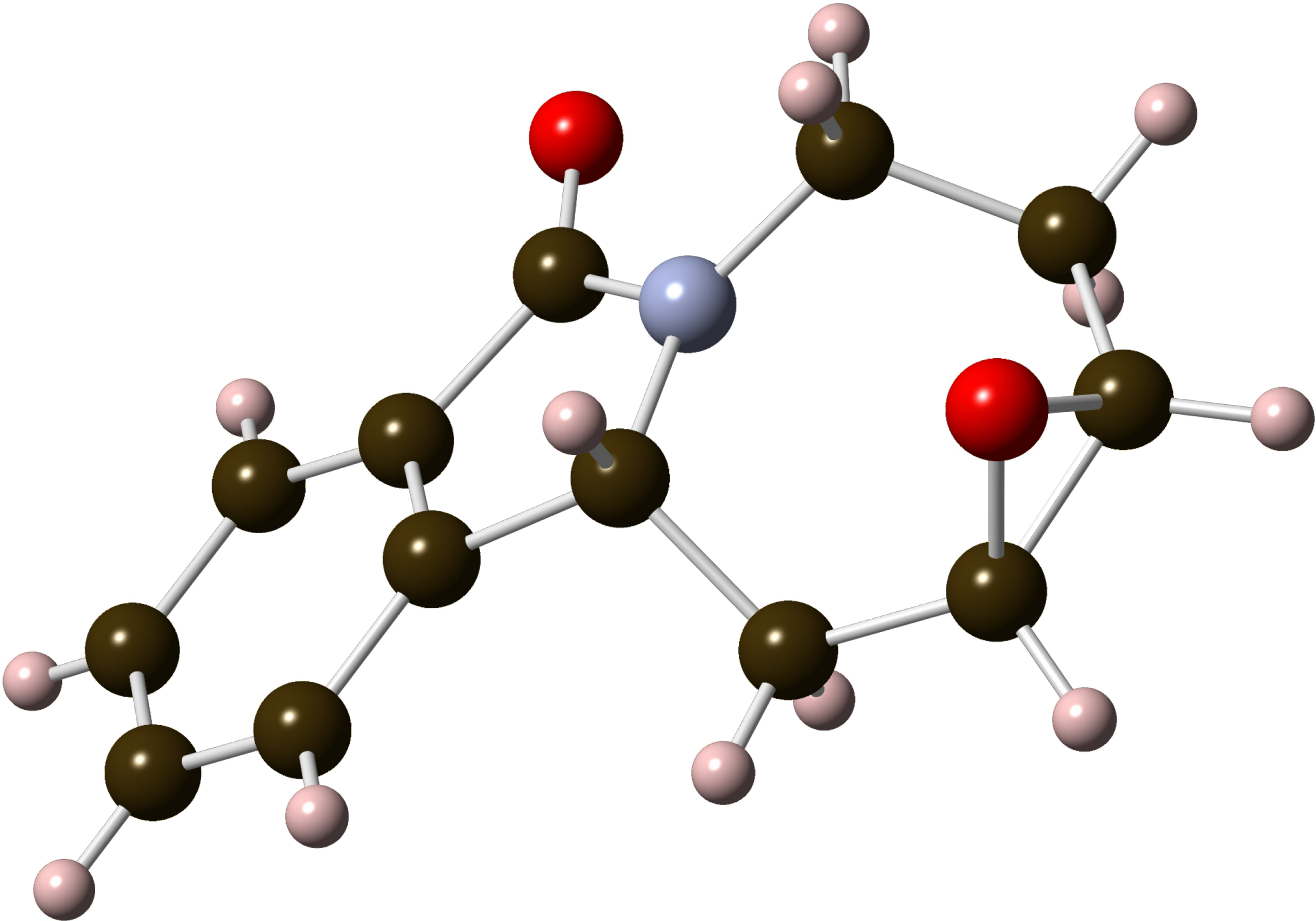
X-Ray structure of epoxide **5**.

Ring-closing metathesis of **2** using Grubbs 2^nd^ generation catalyst [25] in the presence of 1 equiv of Ti(OiPr)_4_ [26–27] at room temperature provided, surprisingly, a modest 45% yield of the alkene-isomerized homoallylic amide **3** instead of the expected allylic amide **4** (Scheme 1). This result was reproduced with Zhan catalyst-1B [28–29], which gave **3** in 50% yield. The structure of alkene **3** was determined based on the X-ray analysis of epoxide **5** (Figure 2), obtained with NaHCO_3_-buffered *meta*-chloroperbenzoic acid (*m*-CPBA) in 57% yield [30–31].

**Figure 3:**
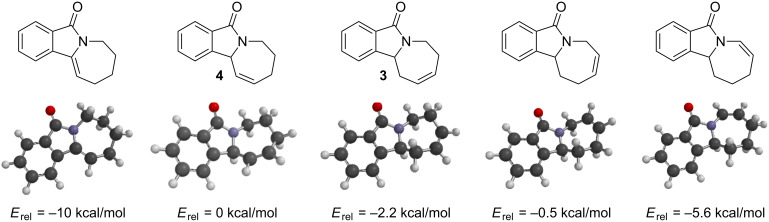
Relative energies of alkene isomers based on RB3LYP/6-311G* calculations with MacSpartan ’06.

The surprising formation of **3** instead of **4** under the metathesis conditions could be explained by a ruthenium-catalyzed double-bond isomerization [32]. The release of ring strain, however, can only be partially responsible for this facile isomerization. DFT calculations of the five possible alkene isomers of **4** indicated a decrease in relative energy from **4** to **3**, but other isomers were even lower in energy (Figure 3). The starting geometries for the alkene isomers prior to DFT optimizations were obtained by a conformational search using the MMFF force field.

**Scheme 2:**
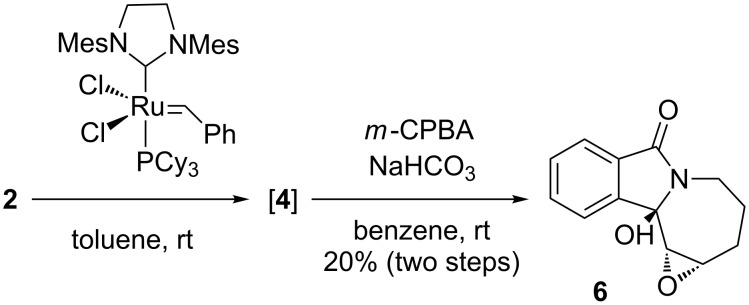
Ring-closing metathesis of diene **2** in the absence of Ti(OiPr)_4_ and isolation of hydroxy epoxide **6** after treatment with *m*-CPBA.

**Figure 4:**
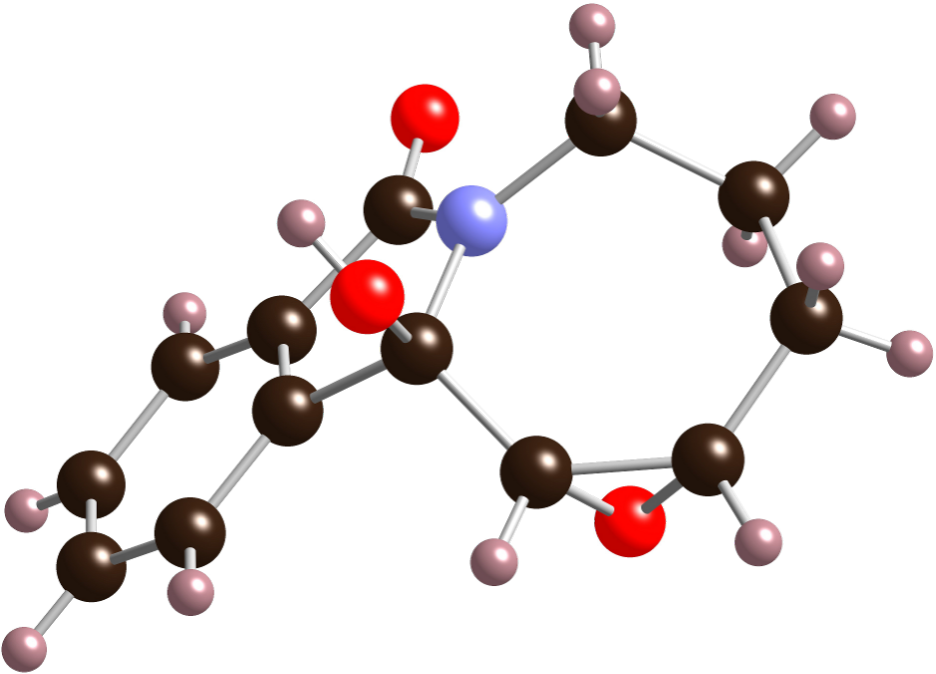
X-Ray structure of epoxyalcohol **6**.

In order to investigate the factors influencing the alkene isomerization process, we conducted a ring-closing metathesis in the absence of Ti(OiPr)_4_ (Scheme 2). The resulting product was different from **3**, based on a TLC analysis, but proved to be quite labile during workup. Therefore, it was immediately subjected to *m*-CPBA epoxidation conditions to give a modest yield of the further oxidized **6**, which was structurally assigned by X-ray analysis (Figure 4). The formation of **6** implies the intermediate presence of alkene **4**, the product of a regular RCM of diene **2**. Accordingly, the isolation of **6**, and the absence of significant quantities of **5**, confirmed the chelating additive Ti(OiPr)_4_ as the primary factor responsible for the isomerization of **4** to **3** in the previous reaction sequence. An additional contributing reason for the exclusive formation of **3** in the earlier metathesis reactions could be the decomposition of the acid-labile isomer **4** under the reaction and chromatographic-purification conditions. A possible pathway for decomposition is indicated by the benzylic/allylic methine oxidation product **6**. The ability of Ti(OiPr)_4_ to induce alkene isomerization during the ring-closing metathesis reaction is noteworthy; while there are a number of additives known to decrease the rate of isomerization in RCM [33–35], we are unaware of any previous report on an alkene-isomerization-promoting effect of an additive in this reaction. We can speculate that the presence of Ti(OiPr)_4_ stabilizes the ruthenium alkylidene complex and, thus allows product isomerization to take place during and after the RCM reaction (see below).

**Scheme 3:**
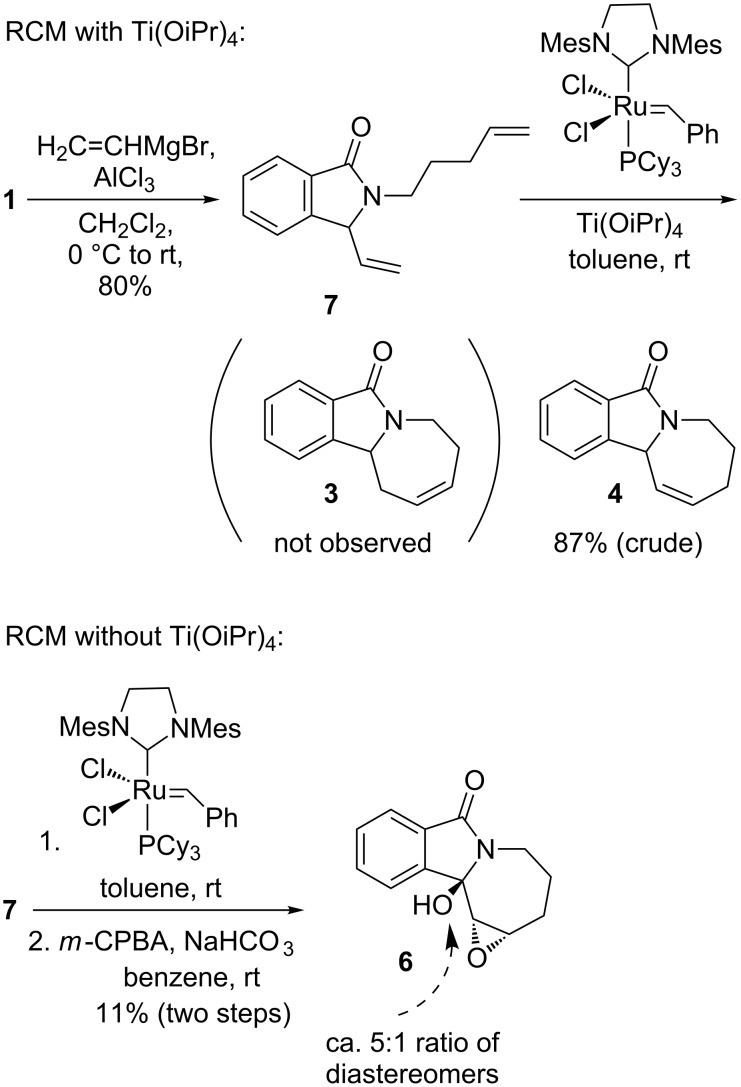
Preparation and RCM reaction of bis-terminal diene analogue **7**.

We also briefly studied the influence of the diene substitution pattern on the rate of isomerization from **4** to **3** and the corresponding product distribution (Scheme 3). Addition of in situ prepared vinyl alane to pivaloate **1** provided the diene **7** in 80% yield. RCM with Grubbs second-generation catalyst in the presence of Ti(OiPr)_4_ led to an exclusive conversion to alkene **4**, i.e., no alkene isomerization was observed in this case, and no homoallylic amide **3** was detected in the reaction mixture. Similarly, in the absence of Ti(OiPr)_4_, crude **4** was obtained in 77% yield (Supporting Information File 1). The different reaction course with alkenes **2** and **7** indicates a role of the ruthenium carbene intermediate in the isomerization. Metathesis of **2** leads to an alkylidene complex, that could form a ruthenium hydride species. In contrast, metathesis of **7** provides a more reactive methylidene complex that is also likely to decompose more quickly and, thus, be unavailable for isomerization of the kinetic product **4** beyond the time span of the completion of the RCM reaction [36]. It was, however, difficult to purify product **4** due to its chemical instability. When the RCM reaction of **7** was conducted on larger scale in the absence of Ti(OiPr)_4_, and the crude intermediate was subjected to *m*-CPBA oxidation, epoxy alcohol **6** was isolated in 11% overall yield. LC–MS as well as NMR analyses suggested a 5:1 ratio of epimers at the hemiacetal carbon. Hydroxylation/oxidation at the benzylic position with *m*-CPBA in air in the presence of bicarbonate has been observed previously, and a radical mechanism was proposed [37].

In summary, these studies suggest that the alkene isomerization from allylic to homoallylic amides under RCM conditions is both dependent on the presence of the Lewis acidic additive Ti(OiPr)_4_ as well as the substitution pattern of the α,ω-diene precursor.

**Scheme 4:**
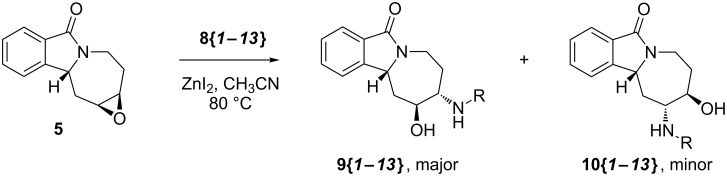
Conversion of epoxide **5** to 1,2-amino alcohols.

**Figure 5:**
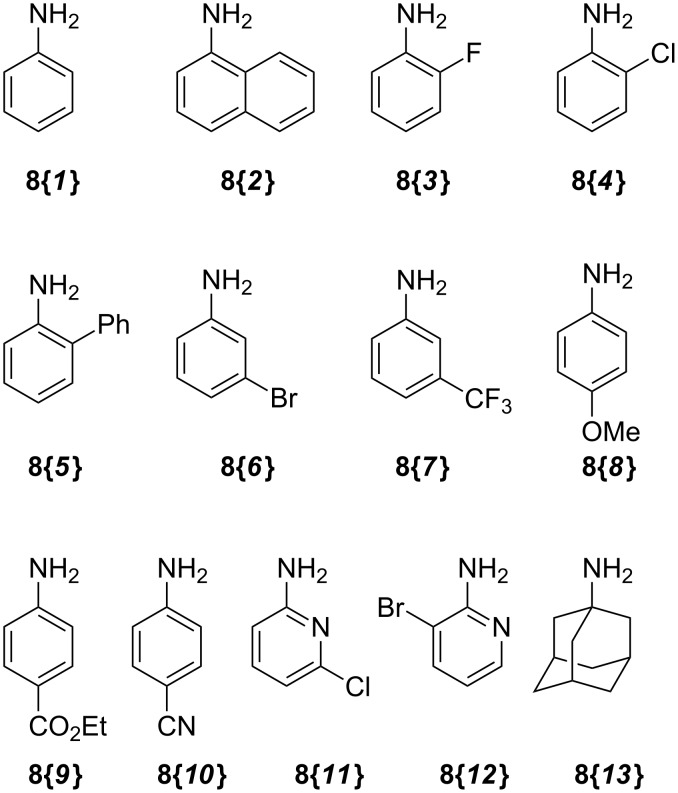
Amine building blocks for library synthesis.

**Figure 6:**
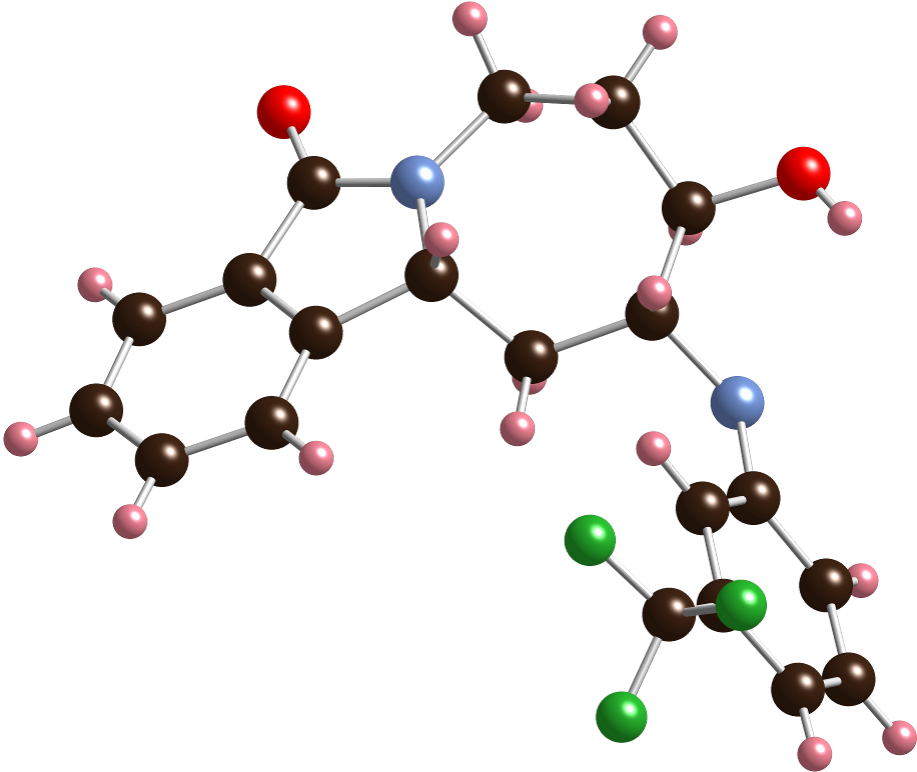
X-ray structure of amino alcohol **10{*7*}**.

With alkene **3** and the corresponding epoxide **5** in hand, a ZnI_2_-mediated amine alkylation protocol could be employed, which introduced a variety of nitrogen nucleophiles **8{*1*–*13*}** (Scheme 4) [38–39]. Co(ClO_4_)_2_ hexahydrate could also be used in place of ZnI_2_, but was generally less efficient (Table 1). Anilines with electron-withdrawing (CF_3_, CN, CO_2_Et), electron-donating (OCH_3_), and halogen substituents (F, Cl, Br) in *ortho*-, *meta*- and *para*-positions were used (Figure 5). Furthermore, aminopyridines **8{*11*}** and **8{*12*}** as well as aliphatic amine **8{*13*}** were compatible with the reaction conditions. With the exception of **8{*13*}**, two regioisomeric products were formed: the major isomer **9{*1*–*12*}** was obtained by an attack on the distal carbon atom of the epoxide, while the minor isomer **10{*1*–*13*}** was obtained by proximal ring opening. These isomers were separated by chromatography on SiO_2_, and an X-ray analysis confirmed the structural assignment for **10{*7*}** (Figure 6). The remainder of the library products were assigned based on the characteristic chemical-shift data for **10{*7*}** and its congener **9{*7*}** [40]. Yields and purities of amino alcohols **9{*1*–*12*}** and **10{*1*–*13*}** are summarized in Table 1.

**Table 1:** Library matrix of products **9{*1*–*13*}** and **10{*1*–*13*}** [isolated yield (%) and purity by ELSD (%)].

	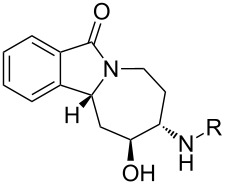	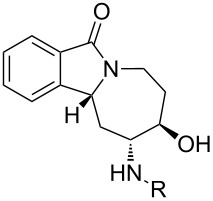

Amine segment R	**9{*1–13*}**	**10{*1–13*}**

**8{*1*}**	56 (>99)	19 (94)^a^
**8{*2*}**	81 (95)	15 (>99)
**8{*3*}**	24 (>99)	10 (99)
**8{*4*}**	72 (99)	17 (99)
**8{*5*}**	44 (99)	33 (95)
**8{*6*}**	69 (>99)	17 (95)
**8{*7*}**	41 (>99)	12 (>99)
**8{*8*}**	58 (99)	26 (98)
**8{*9*}**	85 (99)	15 (99)
**8{*10*}**	59 (>99)	15 (72)
**8{*11*}**	59 (>99)	32 (>99)
**8{*12*}**	20 (99)	–^b^
**8{*13*}**	–^b^	46 (>99)

^a^Co(ClO_4_)_2_·6H_2_O was used in place of ZnI_2_; ^b^Product was not isolated.

The **9{*1*–*11*}**/**10{*1*–*11*}** isolated product ratios varied between 5:1 and 1.5:1, with no obvious trends discernable. Interestingly, for adamantyl amine **8{*13*}**, only the corresponding **10{*13*}** was isolated, most likely due to the steric bulk of the adamantyl group: molecular mechanics calculations indicate that the aminolysis of **5** to regioisomer **10** proceeds with minimal isomerization of the seven-membered ring geometry in the lowest-energy product conformer, whereas the formation of **9** requires a substantial ring flip [41].

## Conclusion

A library of novel tricyclic isoindolinone amino alcohols was prepared in seven steps from commercially available starting materials. Key transformations include the addition of in situ generated alkenylalanes to an *N*-acyliminium ion derived from pivaloate **1**, a tandem ring-closing metathesis–isomerization sequence and a ZnI_2_-mediated epoxide aminolysis. We investigated the factors influencing the alkene isomerization during the RCM process, and identified the presence of the additive Ti(OiPr)_4_, the substitution pattern on the alkene, and the chemical reactivity of the benzylic allylic methine carbon to be significant contributors. Regioisomeric library products **9** and **10** were submitted to the NIH Small Molecule Repository (SMR) [42], screened in the Molecular Libraries Probe Center Network (MLPCN) [43], and biological results were deposited in PubChem [44]. For example, **9{*2*}** was tested in 188 assays and identified as an active hit (based on the hit criteria in the individual test systems) in 4 assays, including a cell-based assay to identify antagonists of the orexin 1 receptor; an assay to identify inhibitors of Apaf-1 (apoptotic peptidase activating factor 1); a cell-based assay to identify antagonists of the human M1 muscarinic receptor; and a cellular assay to identify human immunodeficiency virus 1 inhibitors. Amino alcohol **9{*7*}** was tested in 185 bioassays reported in PubChem, and found to serve as an inhibitor of human platelet activating factor acetylhydrolase 2 (PAFAH2). It is clear from these and other screening data disclosed for this series in the PubChem database that the tricyclic isoindolinone scaffolds hold strong potential for the development of selective and potent lead structures.

## Supporting Information

Supporting information contains experimental procedures for newly synthesized compounds and NMR spectra.

File 1 Experimental procedures and characterization details of synthesized compounds.
