# Supplementary material for: Synthesis of a library of tricyclic azepinoisoindolinones
Source: Beilstein J Org Chem. 2012 Jul 13;8:1091–7. doi: 10.3762/bjoc.8.120 (PMC3458725; doi:10.3762/bjoc.8.120)
Supplement: File 1 — Experimental procedures and characterization details of synthesized compounds. [file Beilstein_J_Org_Chem-08-1091-s001.pdf]

Supporting Information

for

Synthesis of a library of tricyclic azepinoisoindolinones

Bettina Miller, Shuli Mao, Kara M. George Rosenker, Joshua G. Pierce and Peter Wipf*

Address: Center for Chemical Methodologies & Library Development (CMLD), Department of Chemistry, University of Pittsburgh, 219 Parkman Avenue, Pittsburgh, PA 15260

Email: Peter Wipf* - pwipf@pitt.edu

* Corresponding author

Experimental procedures and characterization details of synthesized compounds

General. All glassware was dried in an oven at 140 °C for 2 h prior to use. All air and moisture-sensitive reactions were performed using syringe-septum cap techniques under a dry N_2 or Ar atmosphere. Reactions carried out at 0 °C and below employed a cryocool and an isopropanol/ethanol bath. CH_2Cl_2 and toluene were distilled from CaH. All other materials were obtained from commercial sources and used as received unless otherwise stated.

Reaction visualization was accomplished with a 254 nm UV light or by staining with a solution of $KMnO_4$ (1.5 g of $KMnO_4$, 10 g of K_2CO_3, and 2.5 mL of 5% aq NaOH in 150 mL H_2O). Flash chromatography was performed using SiO_2 (Silicycle, Silia-P Flash Silica Gel, 40–63 μm) or Florisil® (100–200 mesh). Concentrating under reduced pressure refers to the use of a rotary evaporator connected to a membrane vacuum pump or a PIAB Lab Vac H40 to remove solvent. Infrared spectra were determined as neat solids on a Smiths Detection IdentifyIR FT-IR spectrometer. Mass spectra were obtained on a Waters QtoF API US or Thermo Scientific Exactive Orbitrap LC–MS. ^{1}H and ^{13}C NMR spectra were obtained on a Bruker Avance 300 or 500 MHz in $CDCl_3$ unless otherwise noted. Chemical shifts (δ) were reported in parts per million with the residual solvent peak used as an internal standard δ $^{1}H/^{13}C$ (solvent); 7.26/77.16 ($CDCl_3$); and are tabulated as follows: chemical shift, multiplicity (s = singlet, bs = broad singlet, d = doublet, t = triplet, app t = apparent triplet, q = quartet, m = multiplet), number of protons, and coupling constant(s). ^{13}C NMR spectra were obtained at 125 MHz using a proton-decoupled pulse sequence and are tabulated by observed peak. $CDCl_3$ was filtered through dried basic alumina prior to sample preparation. Compounds **1**, **2**, and **3** were prepared previously [1], but compound **3** had been erroneously assigned as **4** in [1].

Products were analyzed by reverse-phase HPLC (Alltech Prevail C-18, 100 × 4.6 mm, 1 mL/min, CH_3CN, H_2O and 0.1% TFA) with UV (210, 220 and 254 nm), ELS (nebulizer 45 °C, evaporator

45 °C, N_2 flow 1.25 SLM), and MS detection using a Thermo Finnigan Surveyor LC and LCQ Advantage MS system (ESI positive mode).

Experimental

7,8,9,11a-Tetrahydro-5*H*-azepino[2,1-*a*]isoindol-5-one (4). To a solution of **7** (0.060 g, 0.26 mmol) in toluene (50 mL) at rt under an atmosphere of N_2 was added Grubbs 2nd generation catalyst (0.011 g, 0.013 mmol). The solution was stirred at rt for 15.5 h. The entire reaction mixture was directly applied to a column of Florisil® and eluted with 100% hexanes and then 30% EtOAc/hexanes to afford **4** (0.041 g, 77%) as a crude yellow oil: ^{1}H NMR ($CDCl_3$, 500 MHz) δ 7.79 (d, 1H, *J* = 7.6 Hz), 7.53 (t, 1H, *J* = 7.5 Hz), 7.47–7.40 (m, 2H), 5.85–5.75 (m, 2H), 5.20 (s, 1H), 4.32 (ddd, 1H, *J* = 13.6, 9.1, 4.2 Hz), 3.35 (ddd, 1H, *J* = 10.6, 6.7, 3.9 Hz), 2.36–2.30 (m, 2H), 2.10–2.01 (m, 1H), 1.95–1.88 (m, 1H); ^{13}C NMR ($CDCl_3$, 125 MHz) δ 168.3, 145.2, 132.2, 131.9, 131.7, 128.2, 127.3, 123.6, 122.0, 60.7, 43.5, 28.4, 26.0; HRMS–ESI (*m*/*z*): $[M + H]^+$ calcd for $C_{13}H_{14}NO$ 200.1070; found, 200.1067.

(1a*RS*,9b*RS*,10a*SR*)-2,3,10,10a-Tetrahydro-1a*H*-oxireno[2',3':4,5]azepino[2,1-*a*]isoindol-5(9b*H*)-one (5). To a microwave vial charged with **3** [1] (20 mg, 0.10 mmol) under an atmosphere of Ar was added benzene (2 mL). The colorless solution was treated with $NaHCO_3$ (0.04 g, 0.5 mmol), followed by *m*-CPBA (0.12 g, 70–75% purity, 0.50 mmol) to give a white suspension. The suspension was stirred at rt for 13.5 h. The jelly-like white mixture was diluted with CH_2Cl_2 and quenched with sat. aq $NaHCO_3$. The layers were separated and the aqueous layer was twice extracted with CH_2Cl_2. The combined organic layers were dried ($MgSO_4$) and concentrated in vacuo. The crude residue was purified by chromatography on SiO_2

(hexanes/EtOAc, 3:1 to 2:1 to 1:1) to provide **5** (12.3 mg, 57%) as a white solid: Mp 186.5–187.1 °C; IR (neat) 2922, 1685 cm^{-1}; ^{1}H NMR ($CDCl_3$, 300 MHz) δ 7.82 (d, 1H, *J* = 7.5 Hz), 7.57–7.52 (m, 1H), 7.48–7.40 (m, 2H), 4.54 (dd, 1H, *J* = 11.9, 2.6 Hz), 4.32 (dt, 1H, *J* = 14.1, 3.9 Hz), 3.36–3.31 (m, 2H), 3.13 (ddd, 1H, *J* = 12.3, 12.0, 1.8 Hz), 2.96–2.87 (m, 1H), 2.50–2.40 (m, 1H), 2.16 (ddd, 1H, *J* = 15.0, 12.3, 3.3 Hz), 1.83 (dd, 1H, *J* = 15.3, 11.7 Hz); ^{13}C NMR ($CDCl_3$, 75 MHz) δ 167.8, 145.3, 131.9, 131.5, 128.2, 123.9, 121.7, 57.7, 55.1, 54.0, 36.4, 34.1, 27.8; HRMS–ESI (*m*/*z*): [M + Na]$^+$ calcd for $C_{13}H_{13}NO_2Na$, 238.0846; found, 238.0866.

(1a*SR*,10b*RS*,10c*SR*)-10b-Hydroxy-3,4,10b,10c-tetrahydro-1a*H*-oxireno[2',3':3,4]azepino[2,1-*a*]isoindol-6(2*H*)-one (6). To a solution of **7** (0.050 g, 0.22 mmol) in toluene (40 mL) at rt under an atmosphere of N_2 was added Grubbs 2nd generation catalyst (0.009 g, 0.01 mmol). The solution was stirred at rt for 14 h. The reaction mixture was concentrated under reduced pressure to provide **4** as a crude residue. The residue was taken on to the next step without further purification.

To a flask containing crude **4** in CH_2Cl_2 (4.0 mL) at rt open to air was added $NaHCO_3$ (0.185 g, 2.20 mmol) and *m*-CPBA (0.542 g, 2.20 mmol). The reaction mixture was stirred at rt open to air for 33 h, diluted with CH_2Cl_2 and washed with sat. aq $NaHCO_3$ and sat. aq Na_2CO_3. The aqueous layers were then extracted with CH_2Cl_2. The combined organic layers were washed with brine, dried ($MgSO_4$), and concentrated under reduced pressure to provide **6** (0.057 g, 11% from **7**) as a colorless residue and a ca. 5:1 mixture of diastereomers by LC–MS/NMR analysis; major diastereomer: ^{1}H NMR ($CDCl_3$, 500 MHz) δ 7.68–7.64 (m, 2H), 7.60 (t, 1H, *J* = 7.5 Hz), 7.49–7.45 (m, 1H), 3.86 (d, 1H, *J* = 14.5 Hz), 3.54 (d, 1H, *J* = 4.2 Hz), 3.46–3.41 (m, 1H), 3.37–3.31 (m, 2H), 2.84–2.77 (m, 1H), 2.49–2.41 (m, 1H), 2.18–2.09 (m, 1H), 1.69–1.61 (m, 1H);

^{13}C NMR ($CDCl_3$, 125 MHz) δ 166.9, 145.9, 132.7, 131.0, 130.3, 123.7, 121.8, 89.6, 59.2, 57.8, 37.9, 28.0, 24.2; HRMS–ESI (*m*/*z*): $[M + H]^+$ calcd for $C_{13}H_{14}NO$, 232.0968; found, 232.0966.

2-(Pent-4-enyl)-3-vinylisoindolin-1-one (7). To a flask containing $AlCl_3$ (3.73 g, 27.9 mmol) in CH_2Cl_2 (16 mL) cooled to 0 °C under an atmosphere of Ar was added vinyl magnesium bromide (1.0 M in THF, 83.6 mL, 8.39 mmol) dropwise over 20 min. The solution was stirred at 0 °C for 4 h. A solution of **1** [1] (3.00 g, 9.95 mmol) in CH_2Cl_2 (9.0 mL) was added in one portion. The reaction mixture was allowed to stir at 0 °C for 1 h and then warmed to rt and stirred for 3 h. The reaction mixture was cooled to 0 °C and carefully quenched with 1 M HCl, warmed to rt, and extracted with CH_2Cl_2. The combined organic extracts were washed with brine, dried ($MgSO_4$), and concentrated under reduced pressure to provide a brown residue. The residue was purified by chromatography on SiO_2 (100% hexanes, 5% EtOAc/hexanes, 10% EtOAc/hexanes) to provide **7** (1.80 g, 80%) as an orange oil: IR (ATR, neat): 3074, 2923, 1685, 1639, 1614, 1596, 1467, 1437, 1400, 1312, 1275, 1094 cm^{-1}; ^1H NMR ($CDCl_3$, 500 MHz) δ 7.83 (d, 1H, *J* = 7.5 Hz), 7.52 (t, 1H, *J* = 7.5 Hz), 7.46 (t, 1H, *J* = 7.5 Hz), 7.34 (d, 1H, *J* = 7.4 Hz) 5.82 (dddd, 1H, *J* = 16.9, 13.2, 10.2, 6.6 Hz), 5.65–5.56 (m, 1H), 5.52–5.42 (m, 2H), 5.04 (d, 1H, *J* = 17.2 Hz), 4.97 (d, 1H, *J* = 10.2 Hz), 4.87 (d, 1H, *J* = 8.2 Hz), 3.87 (ddd, 1H, *J* = 14.5, 7.6, 7.6 Hz), 3.22 (ddd, 1H, *J* = 14.0, 8.6, 5.5 Hz), 2.14–2.05 (m, 2H), 1.80–1.65 (m, 2H); ^{13}C NMR ($CDCl_3$, 125 MHz) δ 168.2, 144.2, 137.8, 135.3, 132.2, 131.6, 128.6, 123.6, 123.1, 120.9, 115.2, 64.3, 40.1, 31.2, 27.7; HRMS–ESI (*m*/*z*): $[M + H]^+$ calcd for $C_{15}H_{18}NO$, 228.1383; found, 228.1380.

General protocol for amine opening of epoxide (5:9*SR*,10*SR*,11a*RS*)-9-((2-chlorophenyl)amino)-10-hydroxy-7,8,9,10,11,11a-hexahydro-5*H*-azepino[2,1-*a*]isoindol-5-

one (**9{*4*}**). To a 2–5 mL vial charged with a solution of **5** (15.3 mg, 0.0710 mmol) in CH_3CN (0.71 mL) under an atmosphere of Ar was added ZnI_2 (45.0 mg, 0.142 mmol). 2-Chloroaniline (80.0 μL, 0.76 mmol) was added dropwise and the resulting yellow solution was placed into a preheated oil bath at 80 °C and stirred for 14.5 h. The solution was cooled to rt and the solvents were removed in vacuo. The crude product was purified by chromatography on SiO_2 (100% CH_2Cl_2, CH_2Cl_2/MeOH, 40:1 to 20:1 to 10:1) to afford **9{*4*}** (17.6 mg, 72%) as a yellow oil and **10{*4*}** (4.1 mg, 17%) as a yellow oil. **9{*4*}**: ^{1}H NMR (300 MHz, $CDCl_3$) δ 7.86 (dd, 1H, *J* = 7.5, 0.9 Hz), 7.64–7.56 (m, 1H), 7.54–7.45 (m, 2H), 7.31 (dd, 1H, *J* = 8.1, 1.5 Hz), 7.19–7.11 (m, 1H), 6.85 (dd, 1H, *J* = 8.4, 1.2 Hz), 6.72 (dt, 1H, *J* = 7.8, 1.5 Hz), 4.86 (t, 1H, *J* = 5.1 Hz), 4.25 (ddd, 1H, *J* = 14.7, 6.0, 3.3 Hz), 4.07 (d, 1H, *J* = 9.6 Hz), 3.68–3.55 (m, 1H), 3.50–3.40 (m, 1H), 3.26 (ddd, 1H, *J* = 14.4, 10.5, 2.1 Hz), 3.04 (s, 1H), 2.52 (ddd, 1H, *J* = 15.0, 7.5, 5.1 Hz), 2.39 (ddd, 1H, *J* = 15.0, 5.1, 2.4 Hz), 2.25 (dddd, 1H, *J* = 14.7, 6.0, 3.6, 2.1 Hz), 1.88 (dtd, 1H, *J* = 13.5, 10.5, 3.3 Hz); ^{13}C NMR (75 MHz, $CDCl_3$) δ 168.6, 145.8, 143.3, 132.7, 132.0, 129.7, 128.7, 128.1, 123.7, 122.4, 121.1, 119.3, 113.5, 69.9, 61.2, 56.7, 38.7, 37.2, 30.2. **(9*RS*,10*RS*,11a*RS*)-10-((2-Chlorophenyl)amino)-9-hydroxy-7,8,9,10,11,11a-hexahydro-5*H*-azepino[2,1-*a*]isoindol-5-one** (**10{*4*}**): ^{1}H NMR (300 MHz, $CDCl_3$) δ 7.83 (d, 1H, *J* = 6.9 Hz), 7.53–7.43 (m, 2H), 7.36–7.22 (m, 3H), 6.97 (dd, 1H, *J* = 8.1, 1.2 Hz), 6.79 (td, 1H, *J* = 7.8, 1.5 Hz), 4.67 (d, 1H, *J* = 9.6 Hz), 4.10 (bs, 1H), 3.88–3.84 (m, 2H), 3.76–3.71 (m, 2H), 3.04–2.98 (m, 1H), 2.65–2.60 (m, 1H), 2.43–2.33 (m, 1H), 2.19–2.12 (m, 1H), 1.51–1.39 (m, 1H).

(9*SR*,10*SR*,11a*RS*)-10-Hydroxy-9-(phenylamino)-7,8,9,10,11,11a-hexahydro-5*H*-azepino[2,1-*a*]isoindol-5-one (**9{*1*}**). According to the general protocol, epoxide **5** (15.0 mg, 0.070 mmol), ZnI_2 (1 mg, 0.003 mmol) and aniline (7.0 μL, 0.070 mmol) were heated at 90 °C in

CH_3CN (0.5 mL) for 76 h. The crude product was purified by chromatography on SiO_2 (CH_2Cl_2/MeOH 40:1) to afford **9{*1*}** (12.0 mg, 56%) as a yellow oil: IR (film) 3371, 2914, 1660 cm^{-1}; ^{1}H NMR (300 MHz, $CDCl_3$) δ 7.82 (d, 1H, *J* = 7.5 Hz), 7.62–7.43 (m, 3H), 7.21 (d, 1H, *J* = 7.5 Hz), 7.18 (d, 1H, *J* = 7.5 Hz), 6.80 (t, 1H, *J* = 7.3 Hz), 6.71 (d, 2H, *J* = 7.5 Hz), 4.81 (t, 1H, *J* = 5.0 Hz), 4.24 (ddd, 1H, *J* = 15.0, 5.5, 3.1 Hz), 3.55–3.42 (m, 1H), 3.34–3.12 (m, 4H), 2.50–2.37 (m, 2H), 2.28–2.18 (m, 1H), 1.86–1.69 (m, 1H); ^{13}C NMR (75 MHz, $CDCl_3$) δ 168.4, 147.0, 145.5, 132.5, 131.8, 129.5, 128.4, 123.3, 122.2, 119.5, 115.0, 69.4, 61.9, 56.7, 38.6, 36.6, 30.4; ESIMS *m*/*z* (% rel. intensity): 309 ([M + H]$^+$, 100), 331 ([M + Na]$^+$, 40), 198 (40); HRMS–ESI (*m*/*z*): [M + H]$^+$ calcd for $C_{19}H_{21}N_2O_2$, 309.1603; found, 309.1613.

(9*RS*,10*RS*,11a*RS*)-9-Hydroxy-10-(phenylamino)-7,8,9,10,11,11a-hexahydro-5*H*-azepino[2,1-*a*]isoindol-5-one (**10**{*1*}). According to the general protocol, **5** (15.0 mg, 0.070 mmol), $Co(ClO_4)_2·6H_2O$ (1.2 mg, 0.007 mmol) and aniline (14.0 μL, 0.167 mmol) were stirred at rt for 90 h and then heated to 90 °C in CH_3CN (0.5 mL) for 48 h. The crude product was purified by chromatography on SiO_2 (CH_2Cl_2/MeOH 20:1) to afford **10**{*1*} (4.0 mg, 19%) as a yellow oil: ^{1}H NMR (300 MHz, $CDCl_3$) δ 7.82 (d, 2H, *J* = 6.9 Hz), 7.53–7.39 (m, 2H), 7.37–7.24 (m, 3H), 6.87 (t, 1H, *J* = 7.4 Hz), 6.78 (d, 1H, *J* = 7.8 Hz), 4.64 (d, 1H, *J* = 10.2 Hz), 3.93–3.82 (m, 2H), 3.69–3.55 (m, 2H), 3.34 (bs, 1H), 3.20 (bs, 1H), 2.67 (d, 1H, *J* = 14.1 Hz), 2.47–2.35 (m, 1H), 2.22–2.07 (m, 1H), 1.39–1.22 (m, 1H).

(9*SR*,10*SR*,11a*RS*)-10-Hydroxy-9-(naphthalen-1-ylamino)-7,8,9,10,11,11a-hexahydro-5*H*-azepino[2,1-*a*]isoindol-5-one (**9{*2*}**). According to the general protocol, **5** (20.0 mg, 0.093 mmol), ZnI_2 (31.4 mg, 0.098 mmol) and 1-naphthylamine (80.5 mg, 0.557 mmol) were heated at 90 °C in CH_3CN (0.5 mL) for 125 h. The crude product was purified by

chromatography on SiO_2 (CH_2Cl_2/MeOH 40:1 to 20:1) to afford **9{*2*}** (27.0 mg, 81%) as a brown oil and **10{*2*}** (5.0 mg, 15%) as a red-brown oil. **9{*2*}**: IR (film) 3362, 2926, 1658 cm^{-1}; ^1H NMR (300 MHz, $CDCl_3$) δ 7.86–7.73 (m, 3H), 7.61–7.28 (m, 7H), 6.78 (dd, 1H, *J* = 5.8, 2.9 Hz), 4.83 (t, 1H, *J* = 5.1 Hz), 4.26 (ddd, 1H, *J* = 15.0, 6.0, 3.0 Hz), 4.16 (bs, 1H), 3.83–3.71 (m, 1H), 3.54 (td, 2 H, *J* = 7.5, 2.1 Hz), 3.30 (ddd, 1H, *J* = 15.0, 10.7, 2.2 Hz), 2.59–2.37 (m, 2H), 2.36–2.26 (m, 1H), 1.95–1.78 (m, 1H); ^{13}C NMR (75 MHz, $CDCl_3$) δ 168.4, 145.6, 142.1, 134.4, 132.3, 131.7, 128.7, 128.4, 126.2, 125.9, 125.2, 124.2, 123.3, 122.2, 119.9, 119.1, 107.0, 69.6, 60.8, 56.5, 38.6, 37.2, 29.7; ESIMS *m*/*z* (% rel. intensity): 359 ($[M + H]^+$, 100), 216 (8), 198 (35), 158 (5), 146 (15); HRMS–ESI (*m*/*z*): $[M + H]^+$ calcd for $C_{23}H_{23}N_2O_2$, 359.1760; found, 359.1747. **(9*RS*,10*RS*,11a*RS*)-9-Hydroxy-10-(naphthalen-1-ylamino)-7,8,9,10,11,11a-hexahydro-5*H*-azepino[2,1-*a*]isoindol-5-one** (**10{*2*}**): ^1H NMR (300 MHz, $CDCl_3$) δ 7.87–7.78 (m, 2H), 7.74 (dd, 1H, *J* = 8.1, 1.2 Hz), 7.51–7.35 (m, 6H), 7.32–7.27 (m, 1H), 6.90 (dd, 1H, *J* = 6.9, 1.5 Hz), 4.69 (d, 1H, *J* = 6.9 Hz), 4.14 (bs, 1H), 3.93–3.76 (m, 4H), 3.28 (bs, 1H), 2.78 (dt, 1H, *J* = 14.4, 2.4 Hz), 2.48–2.37 (m, 1H), 2.26–2.11 (m, 1H), 1.43 (dt, 1H, *J* = 14.4, 10.5 Hz); ^{13}C NMR (75 MHz, $CDCl_3$) δ 168.4, 145.2, 141.7, 134.6, 131.8, 131.5, 128.9, 128.4, 126.3, 126.1, 125.4, 124.4, 123.4, 122.0, 119.8, 107.4, 75.6, 61.7, 58.1, 39.4, 36.7, 31.5; ESIMS *m*/*z* (% rel. intensity): 381 ($[M + Na]^+$, 100), 359 $[M + H]^+$, 70), 198 (15); HRMS–ESI (*m*/*z*): $[M + H]^+$ calcd for $C_{23}H_{23}N_2O_2$, 359.1760; found, 359.1737.

(9*SR*,10*SR*,11a*RS*)-9-(2-Fluorophenylamino)-10-hydroxy-7,8,9,10,11,11a-hexahydro-5*H*-azepino[2,1-*a*]isoindol-5-one (**9{*3*}**). According to the general protocol, **5** (20 mg, 0.093 mmol), ZnI_2 (30 mg, 0.093 mmol) and 2-fluoroaniline (45 μL, 0.46 mmol) were heated at 90 °C in CH_3CN (0.7 mL) for 92 h. The crude product was purified by HPLC ($MeCN/H_2O$ gradient) to

afford **9{*3*}** (7.2 mg, 24%) as a colorless solid and **10{*3*}** (2.9 mg, 10%) as a colorless solid. **9{*3*}**: ^{1}H NMR (300 MHz, $CDCl_3$) δ 7.86 (d, 1H, *J* = 7.5 Hz), 7.65–7.47 (m, 3H), 7.07–6.98 (m, 2H), 6.87 (td, 1H, *J* = 8.3, 1.4 Hz), 6.78–6.72 (m, 1H), 4.83 (t, 1H, *J* = 5.0 Hz), 4.28 (ddd, 1H, *J* = 12.0, 5.5, 3.2 Hz), 3.64–3.46 (m, 2H), 3.33 (td, 1H, *J* = 7.3, 3.0 Hz), 3.32 (ddd, 1H, *J* = 15.0, 11.2, 2.1 Hz), 3.05 (bs, 1H), 2.59–2.41 (m, 2H), 2.29–2.16 (m, 1H), 1.92–1.77 (m, 1H). **(9*RS*,10*RS*,11a*RS*)-10-((2-Fluorophenyl)amino)-9-hydroxy-7,8,9,10,11,11a-hexahydro-5*H*-azepino[2,1-*a*]isoindol-5-one** (**10{*3*}**): ^{1}H NMR (300 MHz, $CDCl_3$) δ 7.82 (d, 1H, *J* = 6.6 Hz), 7.54–7.41 (m, 2H), 7.33 (d, 1H, *J* = 7.2 Hz), 7.14–6.95 (m, 3H), 6.85–6.76 (m, 1H), 4.64 (d, 1H, *J* = 10.5 Hz), 3.91–3.80 (m, 2H), 3.72–3.58 (m, 3H), 3.14 (bs, 1H), 2.68–2.58 (m, 1H), 2.45–2.35 (m, 1H), 2.22–2.07 (m, 1H), 1.45–1.32 (m, 1H).

(9*SR*,10*SR*,11a*RS*)-9-([1,1'-Biphenyl]-2-ylamino)-10-hydroxy-7,8,9,10,11,11a-hexahydro-5*H*-azepino[2,1-*a*]isoindol-5-one (**9{*5*}**). According to the general protocol, **5** (17 mg, 0.079 mmol), ZnI_2 (50 mg, 0.16 mmol) and 2-aminobiphenyl (96 mg, 0.55 mmol) were heated at 90 °C in CH_3CN (0.7 mL) for 28 h. The crude product was purified by chromatography on SiO_2 (CH_2Cl_2/MeOH 40:1 to 20:1) to obtain **9{*5*}** (13.3 mg, 44%) as a dark brown oil and **10{*5*}** (9.9 mg, 33%) as a black oil. **9{*5*}**: ^{1}H NMR (300 MHz, $CDCl_3$) δ 7.79 (d, 1H, *J* = 7.5 Hz), 7.59–7.24 (m, 9H), 7.11 (dd, 1H, *J* = 7.5, 1.5 Hz), 6.94–6.83 (m, 2H), 4.76 (t, 1H, *J* = 5.0 Hz), 4.18 (ddd, 1H, *J* = 12.0, 5.4, 3.3 Hz), 3.62–3.44 (m, 2H), 3.18–2.98 (m, 3H), 2.49–2.33 (m, 2H), 2.20–2.10 (m, 1H), 1.65–1.57 (m, 1H). **(9*RS*,10*RS*,11a*RS*)-10-([1,1'-Biphenyl]-2-ylamino)-9-hydroxy-7,8,9,10,11,11a-hexahydro-5*H*-azepino[2,1-*a*]isoindol-5-one** (**10{*5*}**): ^{1}H NMR (300 MHz, MeOD) δ 7.72 (d, 1H, *J* = 7.5 Hz), 7.60–7.20 (m, 11H), 7.04–6.96 (m, 2H), 6.73 (td,

1H, J = 7.2, 1.2 Hz), 3.84–3.72 (m, 3H), 3.63–3.56 (m, 2H), 2.72 (dt, 1H, J = 14.7, 2.7 Hz), 2.28–2.07 (m, 2H), 1.43 (dt, 1H, J = 15, 10.1 Hz).

(9*SR*,10*SR*,11a*RS*)-9-((3-Bromophenyl)amino)-10-hydroxy-7,8,9,10,11,11a-hexahydro-5*H*-azepino[2,1-*a*]isoindol-5-one (**9{*6*}**). According to the general protocol, **5** (16 mg, 0.074 mmol), ZnI_2 (47 mg, 0.15 mmol) and 3-bromoaniline (0.08 mL, 0.74 mmol) were heated at 80 °C in CH_3CN (0.71 mL) for 22 h and 45 min. The crude product was purified by chromatography on SiO_2 (CH_2Cl_2/MeOH 40:1 to 20:1) to afford **9{*6*}** (20 mg, 69%) as a brown oil and **10{*6*}** (5 mg, 17%) as a yellow oil. **9{*6*}**: IR (neat) 3358, 2920, 1662, 1593 cm^{-1}; ^1H NMR (300 MHz, $CDCl_3$) δ 7.83 (d, 1H, J = 7.8 Hz), 7.62–7.57 (m, 1H), 7.52–7.46 (m, 2H), 7.04 (t, 1H, J = 7.9 Hz), 6.93–6.87 (m, 2H), 6.62 (dd, 1H, J = 7.9, 2.2 Hz), 4.83 (t, 1H, J = 5.1 Hz), 4.24 (ddd, 1H, J = 15.0, 6.0, 3.3 Hz), 3.51–3.40 (m, 2H), 3.36–3.22 (m, 2H), 2.87 (bs, 1H), 2.54–2.36 (m, 2H), 2.29–2.18 (m, 1H), 1.88–1.75 (m, 1H); ^{13}C NMR (75 MHz, $CDCl_3$) δ 168.7, 148.7, 145.7, 132.7, 132.1, 131.0, 128.7, 123.7, 123.6, 122.4, 122.3, 117.5, 113.8, 69.7, 61.5, 56.7, 38.7, 37.1, 30.3; ESIMS *m*/*z* (% rel. intensity): 342 ($[M]^+$, 14), 215 (29), 166 (100), 145 (61), 69 (55); HRMS–ESI (*m*/*z*): $[M + Na]^+$ calcd for $C_{19}H_{19}BrN_2O_2Na$, 409.0528; found, 409.0492. **(9*RS*,10*RS*,11a*RS*)-10-((3-Bromophenyl)amino)-9-hydroxy-7,8,9,10,11,11a-hexahydro-5*H*-azepino[2,1-*a*]isoindol-5-one** (**10{*6*}**): Characteristic peaks: ^1H NMR (300 MHz, $CDCl_3$) δ 7.86 (d, 1H, J = 6.6 Hz), 7.59–7.42 (m, 2H), 7.34 (d, 1H, J = 7.5 Hz), 7.09 (t, 1H, J = 7.9 Hz), 6.99–6.96 (m, 1H), 6.92 (bs, 1H), 6.69–6.66 (m, 1H), 4.68 (d, 1H, J = 9.3 Hz), 3.88–3.84 (m, 2H), 3.66–3.61 (m, 2H), 2.68–2.63 (m, 1H), 2.42–2.37 (m, 1H), 2.18–2.09 (m, 1H).

(9*SR*,10*SR*,11a*RS*)-10-Hydroxy-9-((3-(trifluoromethyl)phenyl)amino)-7,8,9,10,11,11a-hexahydro-5*H*-azepino[2,1-*a*]isoindol-5-one (**9{*7*}**). According to the general protocol, **5** (21.2 mg, 0.098 mmol), ZnI_2 (62.9 mg, 0.197 mmol) and 3-aminobenzotrifluoride (0.12 mL, 0.98 mmol) were heated at 90 °C in CH_3CN (1 mL) for 4 h. The crude product was purified by chromatography on SiO_2 (CH_2Cl_2/MeOH 40:1 to 20:1) to afford **9{*7*}** (15.3 mg, 41%) as an off-white solid and **10{*7*}** (4.5 mg, 12%) as an off-white solid. **9{*7*}**: Mp 178.0–178.5 °C; IR (neat) 3336, 2926, 1659, 1612 cm^{-1}; ^1H NMR (300 MHz, $CDCl_3$) δ 7.77 (d, 1H, *J* = 7.5 Hz), 7.60–7.55 (m, 1H), 7.51–7.42 (m, 2H), 7.30–7.25 (m, 1H), 7.02 (d, 1H, *J* = 7.8 Hz), 6.93 (s, 1H), 6.86 (d, 1H, *J* = 8.1 Hz), 4.85 (t, 1H, *J* = 5.0 Hz), 4.21 (ddd, 1H, *J* = 15.0, 6.5, 3.1 Hz), 3.82 (d, 1H, *J* = 9.3 Hz), 3.65–3.57 (m, 1H), 3.39 (t, 1H, *J* = 7.3 Hz), 3.27 (ddd, 1H, *J* = 15.0, 10.4, 2.3 Hz), 3.08 (s, 1H), 2.56–2.47 (m, 1H), 2.42–2.35 (m, 1H), 2.31–2.24 (m, 1H), 1.91–1.78 (m, 1H); ^{13}C NMR (75 MHz, $CDCl_3$) δ 168.7, 147.7, 145.8, 132.6, 132.0, 130.1, 128.7, 128.0 (q, *J* = 279.8 Hz), 123.5, 122.4, 117.8, 115.7 (q, *J* = 3.8 Hz), 110.8 (q, *J* = 3.8 Hz), 69.6, 61.0, 56.6, 38.6, 37.1, 29.7; ESIMS *m*/*z* (% rel. intensity): 376 ($[M]^+$, 48), 332 (45), 200 (100), 145 (47); HRMS–ESI (*m*/*z*): $[M]^+$ calcd for $C_{20}H_{19}F_3N_2O_2$, 376.1399; found, 376.1401. **(9*RS*,10*RS*,11a*RS*)-9-Hydroxy-10-((3-(trifluoromethyl)phenyl)amino)-7,8,9,10,11,11a-hexahydro-5*H*-azepino[2,1-*a*]isoindol-5-one** (**10{*7*}**): Mp 206.9–207.8 °C; IR (neat) 3362, 2922, 1664, 1612 cm^{-1}; ^1H NMR (300 MHz, $CDCl_3$) δ 7.82 (dd, 1H, *J* = 6.4, 1.4 Hz), 7.52–7.42 (m, 2H), 7.36–7.31 (m, 2H), 7.08 (d, 1H, *J* = 7.5 Hz), 6.93–6.88 (m, 2H), 4.69 (d, 1H, *J* = 9.9 Hz), 3.86–3.82 (m, 2H), 3.68–3.65 (m, 3H), 2.98 (s, 1H), 2.66 (dt, 1H, *J* = 14.7, 2.1 Hz), 2.38 (dt, 1H, *J* = 11.1, 3.9 Hz), 2.21–2.09 (m, 1H), 1.42 (dt, 1H, *J* = 14.4, 9.9 Hz); ^{13}C NMR (75 MHz, $CDCl_3$) δ 168.6, 147.3, 145.3, 132.4, 132.0, 131.8, 130.3, 128.8, 124.3 (q, *J* = 271.5 Hz), 123.7, 122.1,

116.0 (q, J = 3.8 Hz), 111.0 (q, J = 3.8 Hz), 75.7, 61.8, 58.1, 39.5, 37.0, 31.5; ESIMS m/z (% rel. intensity): 376 ($[M]^+$, 40), 357 (27), 200 (20), 189 (100), 146 (84).

(9*SR*,10*SR*,11a*RS*)-10-Hydroxy-9-((4-methoxyphenyl)amino)-7,8,9,10,11,11a-hexahydro-5*H*-azepino[2,1-*a*]isoindol-5-one (**9{*8*}**). According to the general protocol, **5** (19.3 mg, 0.093 mmol), ZnI_2 (57.2 mg, 0.179 mmol) and *p*-anisidine (82.9 mg, 0.717 mmol) were heated at 90 °C in CH_3CN (0.7 mL) for 20 h. The crude product was purified by chromatography on SiO_2 (CH_2Cl_2/MeOH 60:1 to 30:1) to obtain **9{*8*}** (17.6 mg, 58%) as a dark brown oil and **10{*8*}** (8.0 mg, 26%) as a black oil. **9{*8*}**: IR (film) 3351, 2926, 1662, 1508 cm^{-1}; ^1H NMR (300 MHz, $CDCl_3$) δ 7.82 (d, 1H, J = 7.2 Hz), 7.61–7.43 (m, 3H), 6.82–6.62 (m, 4H), 4.78 (t, 1H, J = 4.9 Hz), 4.24 (ddd, 1H, J = 15.0, 5.1, 3.3 Hz), 3.75 (s, 4H), 3.26 (ddd, 1H, J = 12.0, 8.4, 3.9 Hz), 3.18–3.06 (m, 3H), 2.52–2.42 (m, 2H), 2.27–2.16 (m, 1H), 1.82–1.67 (m, 1H); ^{13}C NMR (75 MHz, $CDCl_3$) δ 168.4, 153.7, 145.5, 140.7, 132.5, 131.8, 128.4, 123.3, 122.2, 117.3, 116.4, 114.9, 114.8, 69.2, 64.0, 56.8, 55.7, 38.6, 36.4, 30.7; ESIMS m/z (% rel. intensity): 339 ($[M + H]^+$, 100); HRMS–ESI (m/z): $[M + H]^+$ calcd for $C_{20}H_{23}N_2O_3$, 339.1706; found, 339.1709. **(9*RS*,10*RS*,11a*RS*)-9-Hydroxy-10-((4-methoxyphenyl)amino)-7,8,9,10,11,11a-hexahydro-5*H*-azepino[2,1-*a*]isoindol-5-one** (**10{*8*}**): ^1H NMR (300 MHz, $CDCl_3$) δ 7.74 (d, 1H, J = 7.5 Hz), 7.61–7.42 (m, 3H), 6.87–6.73 (m, 4H), 4.95–4.85 (m, 1H), 3.84–3.77 (m, 2H), 3.75 (s, 3H), 3.71–3.56 (m, 2H), 3.47–3.38 (m, 4H), 2.66 (ddd, 1H, J = 15.0, 3.1, 1.9 Hz), 2.27–2.15 (m, 2H), 1.45 –1.28 (m, 1H).

Ethyl 4-(((9*SR*,10*SR*,11a*RS*)-10-hydroxy-5-oxo-7,8,9,10,11,11a-hexahydro-5*H*-azepino[2,1-*a*]isoindol-9-yl)amino)benzoate 9{*9*}. According to the general protocol, **5** (20 mg,

0.093 mmol), ZnI_2 (123 mg, 0.375 mmol) and ethyl 4-aminobenzoate (93.0 mg, 0.557 mmol) were heated at 90 °C in CH_3CN (0.5 mL) for 93 h. The crude product was purified by chromatography on SiO_2 (CH_2Cl_2/MeOH, 60:1 to 40:1 to 20:1) to afford **9{*9*}** (5.4 mg, 85%) as a yellow-orange foam and **10{*9*}** (5.4 mg, 15%) as a yellow oil. **9{*9*}**: ^{1}H NMR (300 MHz, $CDCl_3$) δ 7.85 (d, 2H, *J* = 6.9 Hz), 7.79 (d, 1H, *J* = 7.5 Hz), 7.59–7.42 (m, 3H), 6.64 (d, 1H, *J* = 8.7 Hz), 4.86 (t, 1 H, *J* = 5.4 Hz), 4.28 (q, 2H, *J* = 7.2 Hz), 4.21–4.11 (m, 1H), 4.05 (m, 1H), 3.73 (m, 1H), 3.52–3.44 (m, 1H), 3.35–3.10 (m, 1H), 2.52–2.43 (m, 1H), 2.38–2.22 (m, 2H), 1.91–1.82 (m, 1H), 1.48–1.22 (m, 4H). **Ethyl 4-(((9*RS*,10*RS*,11a*RS*)-9-hydroxy-5-oxo-7,8,9,10,11,11a-hexahydro-5*H*-azepino[2,1-*a*]isoindol-10-yl)amino)benzoate** (**10{*9*}**): ^{1}H NMR (300 MHz, $CDCl_3$) δ 7.91 (d, 2H, *J* = 8.7 Hz), 7.81 (d, 1H, *J* = 6.9 Hz), 7.52–7.42 (m, 2H), 7.32 (d, 1H, *J* = 7.2 Hz), 6.69 (d, 2H, *J* = 9.0 Hz), 4.68 (d, 1H, *J* = 9.0 Hz), 4.34 (q, 2H, *J* = 7.2 Hz), 3.93 (bs, 1H), 3.88–3.62 (m, 3H), 2.94 (bs, 1H), 2.65 (dt, 1H, *J* = 14.4, 2.5 Hz), 2.42–2.30 (m, 1H), 2.22–2.07 (m, 1H), 1.38 (t, 3H, *J* = 7.2 Hz) 1.32–1.22 (m, 2H).

4-(((9*SR*,10*SR*,11a*RS*)-10-Hydroxy-5-oxo-7,8,9,10,11,11a-hexahydro-5*H*-azepino[2,1-*a*]isoindol-9-yl)amino)benzonitrile (**9{*10*}**). According to the general protocol, **5** (20 mg, 0.093 mmol), ZnI_2 (59 mg, 0.19 mmol) and 4-aminobenzonitrile (56 mg, 0.46 mmol) were heated at 80 °C in CH_3CN (0.93 mL) for 23 h. The crude product was purified by chromatography on SiO_2 (CH_2Cl_2/MeOH, 40:1 to 20:1) to afford **9{*10*}** (14.8 mg, 59%) as a yellow oil and **10{*10*}** (3.7 mg, 15%) as a brown oil. **9{*10*}**: ^{1}H NMR (300 MHz, CD_3OD) δ 7.74 (d, 1H, *J* = 7.5 Hz), 7.59–7.53 (m, 1H), 7.48–7.41 (m, 2H), 7.40–7.36 (m, 2H), 6.67 (dt, 2H, *J* = 8.7, 1.8 Hz), 4.98 (dd, 1H, *J* = 8.7, 2.4 Hz), 4.03 (bs, 2H), 3.99–3.93 (m, 1H), 3.84–3.83 (m, 2H), 3.48 (ddd, 1H, *J* = 15.0, 7.3, 2.9 Hz), 2.48–2.35 (m, 2H), 1.99–1.86 (m, 2H). **4-**

(((9*RS*,10*RS*,11a*RS*)-9-Hydroxy-5-oxo-7,8,9,10,11,11a-hexahydro-5*H*-azepino[2,1-*a*]isoindol-10-yl)amino)benzonitrile (10{*10*}): Characteristic peaks: ^{1}H NMR (300 MHz, CD_3OD) δ 7.73 (d, 1H, *J* = 7.5 Hz), 7.58–7.40 (m, 5H), 6.75 (d, 1H, *J* = 9.0 Hz), 4.94 (d, 1H, *J* = 12.6 Hz), 3.87–3.71 (m, 4H), 2.64–2.58 (m, 1H), 2.24–2.20 (m, 2H), 1.58–1.43 (m, 1H).

(9*SR*,10*SR*,11a*RS*)-9-((6-Chloropyridin-2-yl)amino)-10-hydroxy-7,8,9,10,11,11a-hexahydro-5*H*-azepino[2,1-*a*]isoindol-5-one (**9{*11*}**). According to the general protocol, **5** (15.7 mg, 0.073 mmol), 6-chloro-2-aminopyridine (44 mg, 0.34 mmol) and ZnI_2 (46.6 mg, 0.15 mmol) in CH_3CN (0.73 mL) were heated at 80 °C for 41 h. The crude product was purified by chromatography on SiO_2 (CH_2Cl_2/MeOH, 40:1 to 20:1) to afford **9{*11*}** (14.9 mg, 59%) as a yellow solid and **10{*11*}** (7.9 mg, 32%) as a red solid. **9{*11*}**: ^{1}H NMR (300 MHz, $CDCl_3$) δ 7.75 (d, 1H, *J* = 7.5 Hz), 7.58–7.52 (m, 1H), 7.48–7.40 (m, 2H), 7.33 (t, 1H, *J* = 7.7 Hz), 6.61 (d, 1H, *J* = 7.5 Hz), 6.40 (d, 1H, *J* = 8.4 Hz), 4.94–4.89 (m, 2H), 4.33 (ddd, 1H, *J* = 15.0, 7.5, 3.0 Hz), 4.23–4.17 (m, 2H), 3.74 (t, 1H, *J* = 5.4 Hz), 3.36 (ddd, 1H, *J* = 15.0, 9.3, 2.7 Hz), 2.52 (ddd, 1H, *J* = 15.0, 6.1, 3.7 Hz), 2.30–2.20 (m, 1H), 2.11 (ddd, 1H, *J* = 15.0, 7.3, 2.3 Hz), 2.02–1.90 (m, 1H). **(9*RS*,10*RS*,11a*RS*)-10-((6-Chloropyridin-2-yl)amino)-9-hydroxy-7,8,9,10,11,11a-hexahydro-5*H*-azepino[2,1-*a*]isoindol-5-one** (**10{*11*}**): ^{1}H NMR (300 MHz, CD_3OD) δ 7.72 (d, 1H, *J* = 7.5 Hz), 7.57–7.42 (m, 4H), 7.31 (t, 1H, *J* = 7.8 Hz), 6.52 (d, 1H, *J* = 7.5 Hz), 6.37 (d, 1H, *J* = 8.1 Hz), 4.91 (d, 1H, *J* = 8.1 Hz), 4.20–4.13 (m, 1H), 3.80–3.70 (m, 4H), 2.73 (dt, 1H, *J* = 9.3, 2.6 Hz), 2.22–2.16 (m, 2H), 1.55 (dt, 1H, *J* = 14.4, 10.4 Hz).

(9*S*,10*S*,11a*R*)-9-((3-Bromopyridin-2-yl)amino)-10-hydroxy-7,8,9,10,11,11a-hexahydro-5*H*-azepino[2,1-*a*]isoindol-5-one (**9{*12*}**). According to the general protocol, **5** (25 mg, 0.12 mmol),

ZnI_2 (74 mg, 0.23 mmol) and 2-amino-3-bromopyridine (0.10 g, 0.58 mmol) were heated at 80 °C in CH_3CN (1.4 mL) for 41.5 h. The crude product was purified by chromatography on SiO_2 (CH_2Cl_2/MeOH, 40:1 to 20:1 to 10:1) to afford **9{*12*}** (4.1 mg, 20%) as a yellow oil: ^{1}H NMR (300 MHz, $CDCl_3$) δ 8.03 (dd, 1H, *J* = 4.9, 1.7 Hz), 7.85 (d, 1H, *J* = 7.2 Hz), 7.67 (dd, 1H, *J* = 7.5, 1.5 Hz), 7.61–7.43 (m, 3H), 6.55 (dd, 1H, *J* = 7.6, 5.0 Hz), 5.06–5.00 (m, 1H), 4.92 (dd, 1H, *J* = 7.2, 3.3 Hz), 4.49 (ddd, 1H, *J* = 15.0, 6.0, 3.0 Hz), 4.30–4.21 (m, 1H), 3.76 (td, 1H, *J* = 6.0, 2.4 Hz), 3.38–3.29 (m, 1H), 2.54 (ddd, 2 H, *J* = 15.0, 6.0, 3.6 Hz), 2.21–2.09 (m, 3H).

(9*RS*,10*R*,11a*R*)-10-((3*S*,5*S*,7*S*)-adamantan-1-ylamino)-9-hydroxy-7,8,9,10,11,11a-hexahydro-5*H*-azepino[2,1-*a*]isoindol-5-one (**10{*13*}**). According to general protocol, **5** (17 mg, 0.079 mmol), ZnI_2 (50 mg, 0.158 mmol) and 1-adamantylamine (86 mg, 0.553 mmol) were heated at 90 °C in CH_3CN (0.7 mL) for 28 h. The crude product was purified by chromatography on SiO_2 (CH_2CL_2/MeOH 40:1) to obtain **10{*13*}** (13.2 mg, 46%) as a yellow foam: ^{1}H NMR (300 MHz, $CDCl_3$) δ 7.78 (d, 1H, *J* = 7.5 Hz), 7.58–7.42 (m, 3H), 4.68 (d, 1H, *J* = 6 Hz), 4.25 (dt, 1H, *J* = 14.4, 3.5 Hz) 3.04 (dt, 1H, *J* = 12.8, 1.8 Hz), 2.67–2.51 (m, 3H), 2.37–2.27 (m, 1H), 2.15–2.03 (m, 5H) 1.73–1.51 (m, 14H).

References

1. Pierce, J. G.; Waller, D. L.; Wipf, P. *J. Organomet. Chem.* **2007,** *692*, 4618–4629. doi:10.1016/j.jorganchem.2007.05.035

4

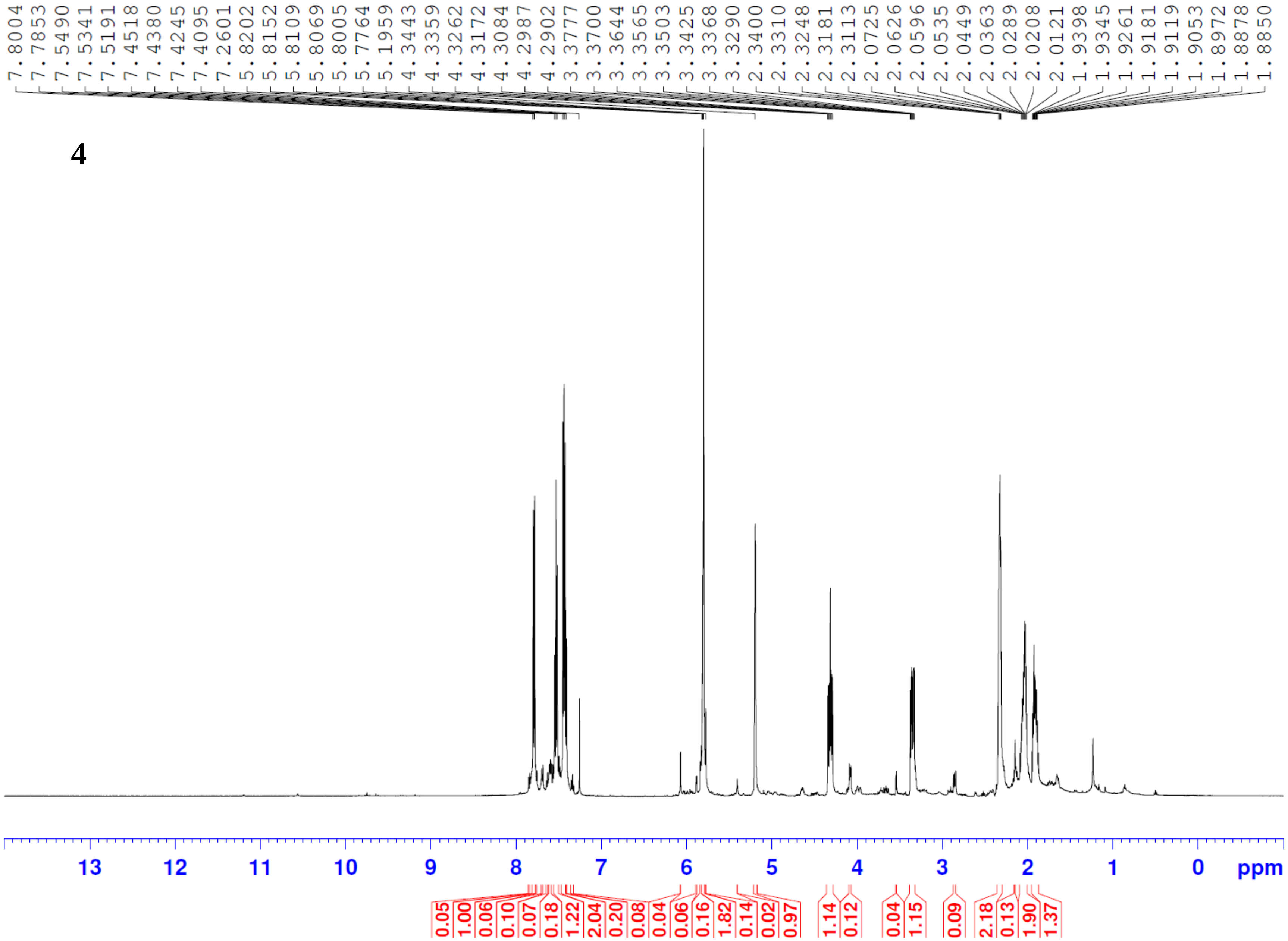

4

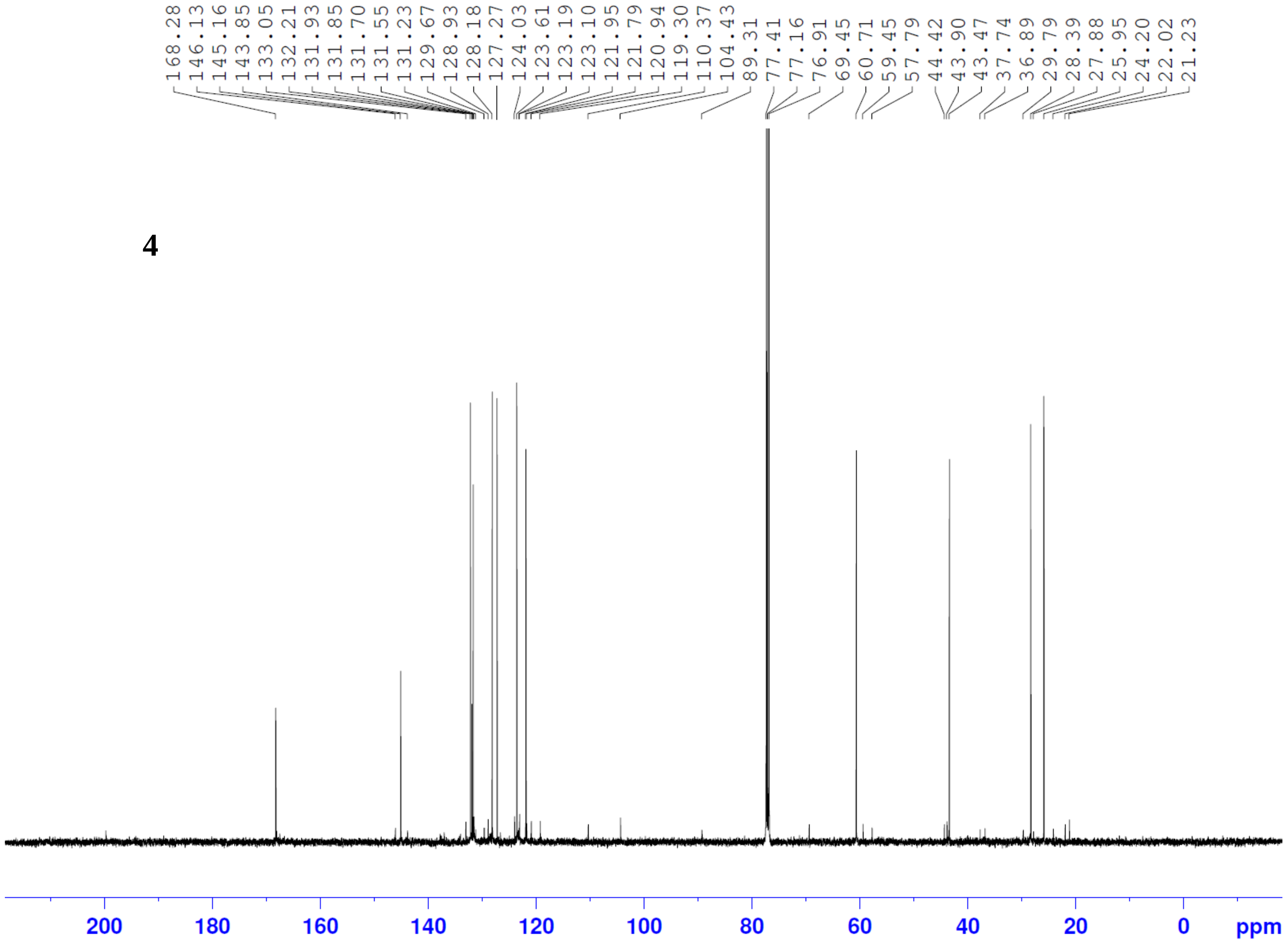
168.28
146.13
145.16
143.85
133.05
132.21
131.93
131.85
131.70
131.55
131.23
129.67
128.93
128.18
127.27
124.03
123.61
123.19
123.10
121.95
121.79
120.94
119.30
110.37
104.43
89.31
77.41
77.16
76.91
69.45
60.71
59.45
57.79
44.42
43.90
43.47
37.74
36.89
29.79
28.39
27.88
25.95
24.20
22.02
21.23
200
180
160
140
120
100
80
60
40
20
0
ppm

5

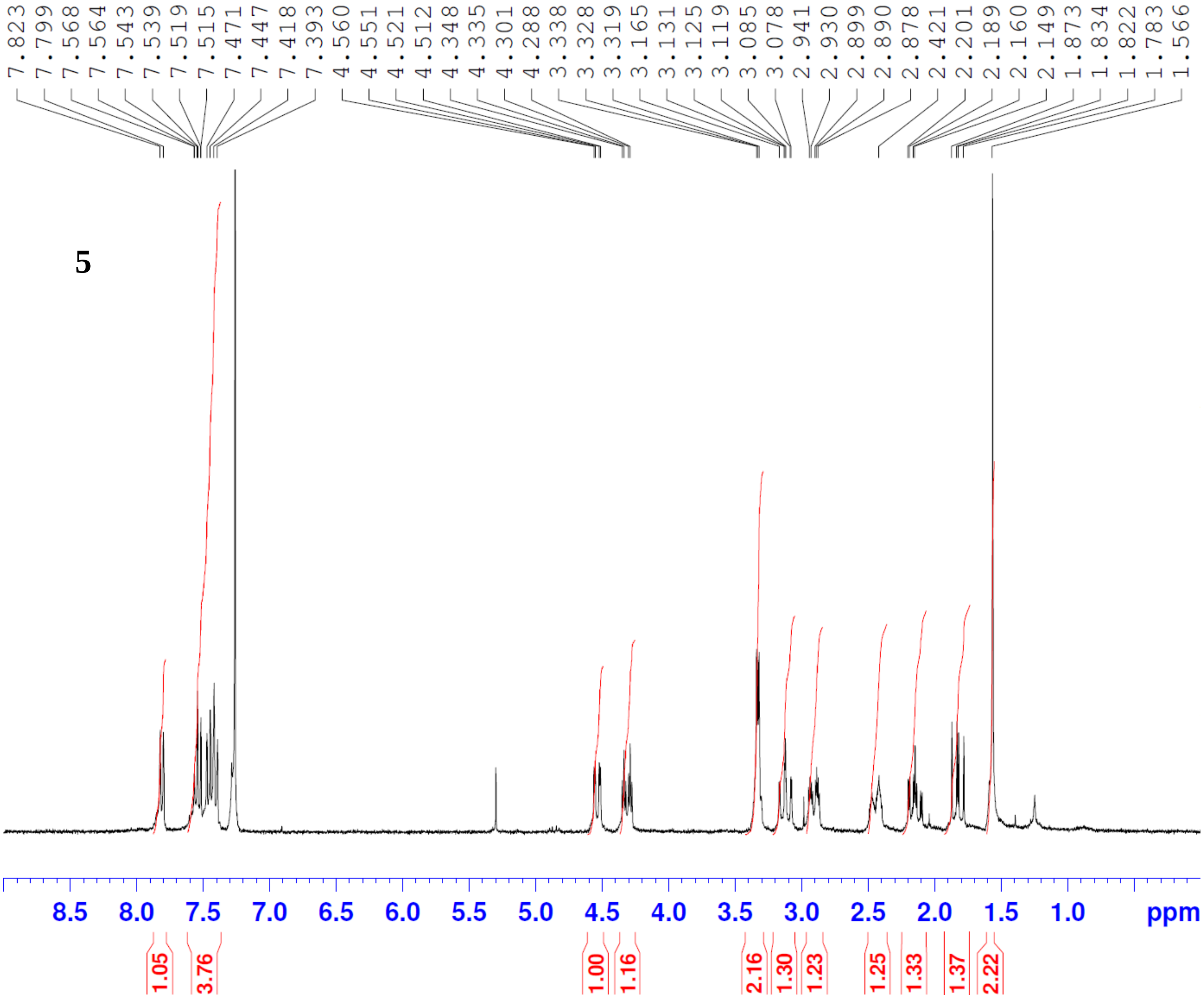
7.823
7.799
7.568
7.564
7.543
7.539
7.519
7.515
7.471
7.447
7.418
7.393
4.560
4.551
4.521
4.512
4.348
4.335
4.301
4.288
3.338
3.328
3.319
3.165
3.131
3.125
3.119
3.085
3.078
2.941
2.930
2.899
2.890
2.878
2.421
2.201
2.189
2.160
2.149
1.873
1.834
1.822
1.783
1.566
8.5
8.0
7.5
7.0
6.5
6.0
5.5
5.0
4.5
4.0
3.5
3.0
2.5
2.0
1.5
1.0
ppm
1.05
3.76
1.00
1.16
2.16
1.30
1.23
1.25
1.33
1.37
2.22

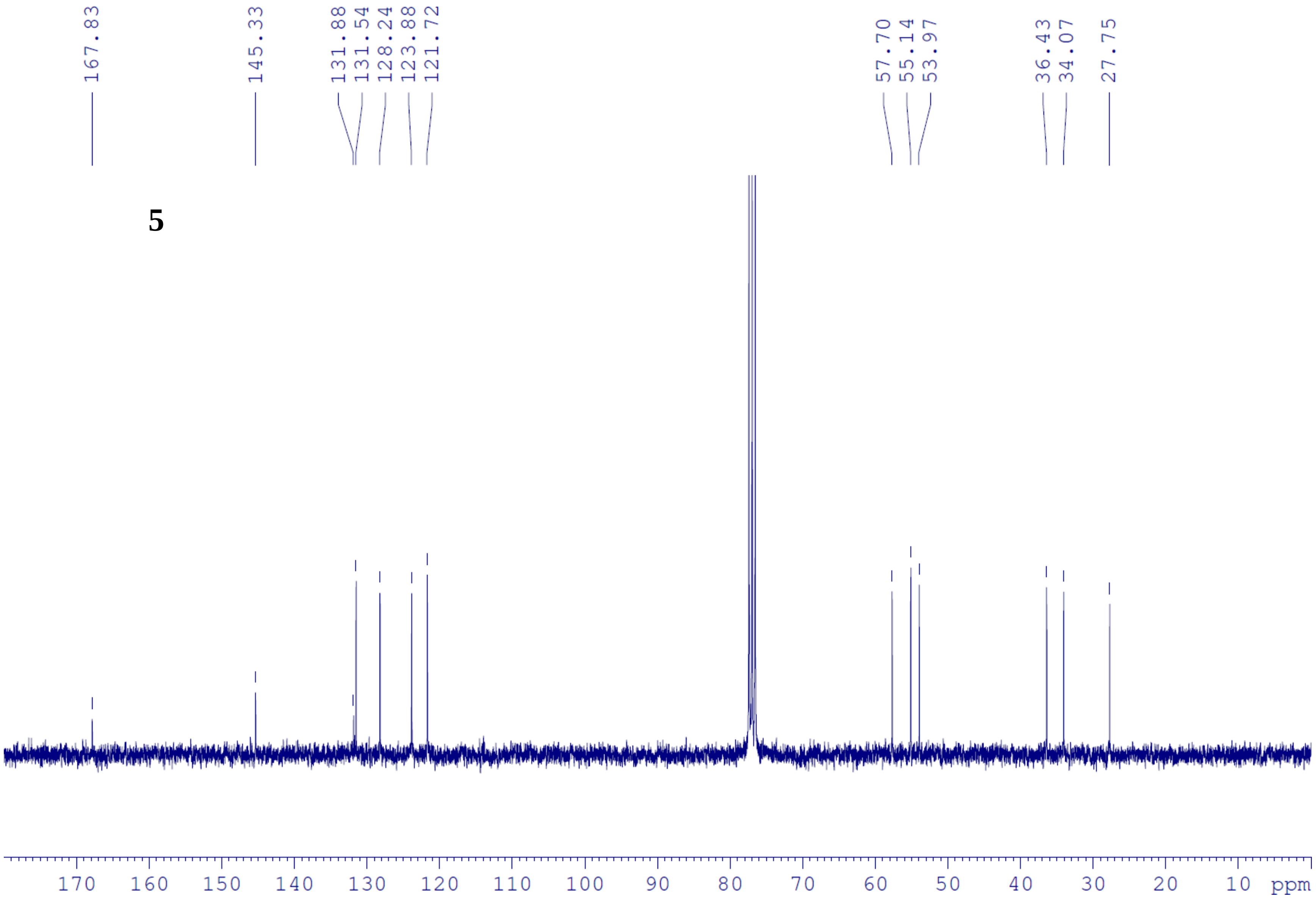
167.83
145.33
131.88
131.54
128.24
123.88
121.72
57.70
55.14
53.97
36.43
34.07
27.75
5
170
160
150
140
130
120
110
100
90
80
70
60
50
40
30
20
10
ppm

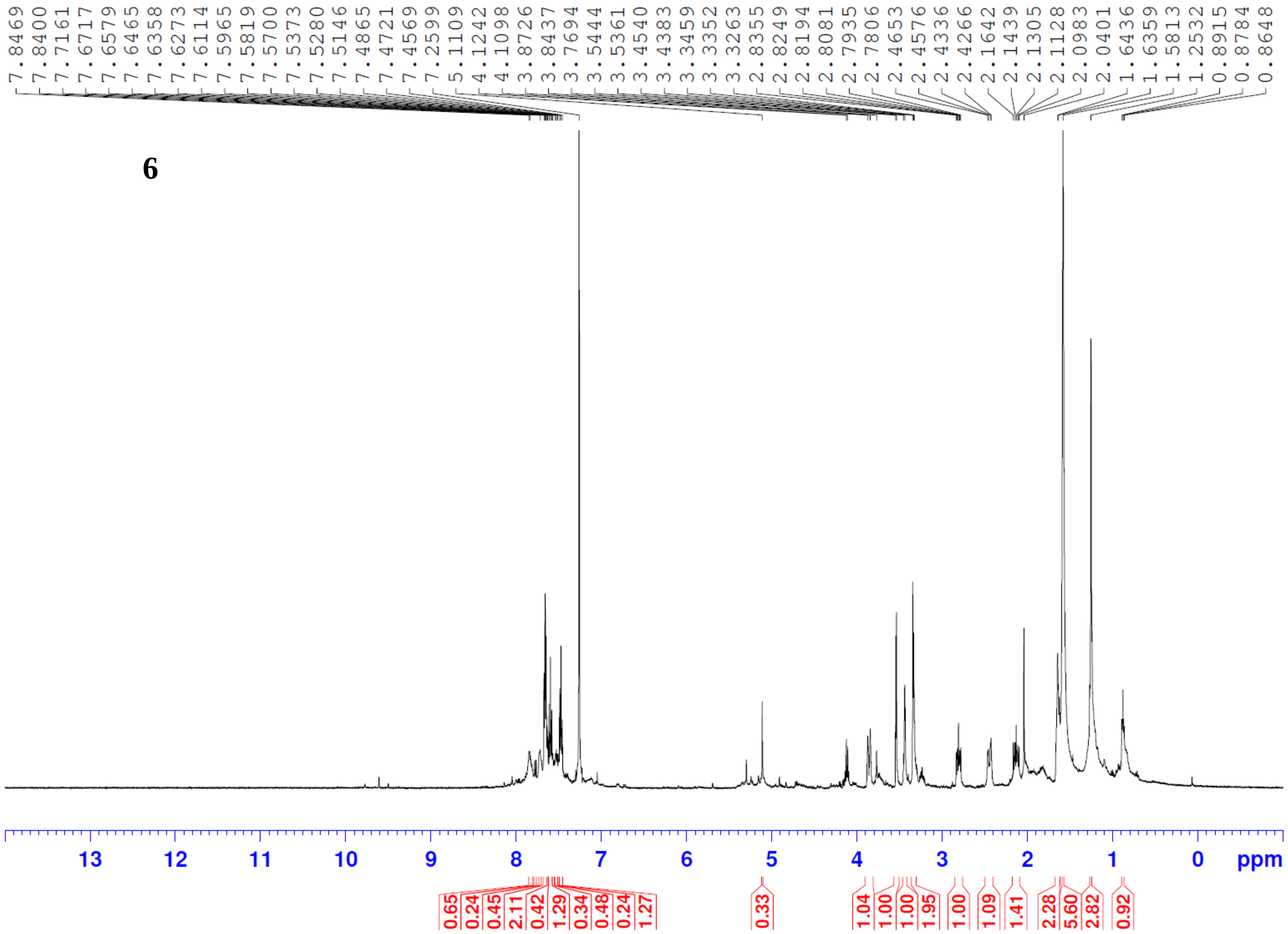

6

6

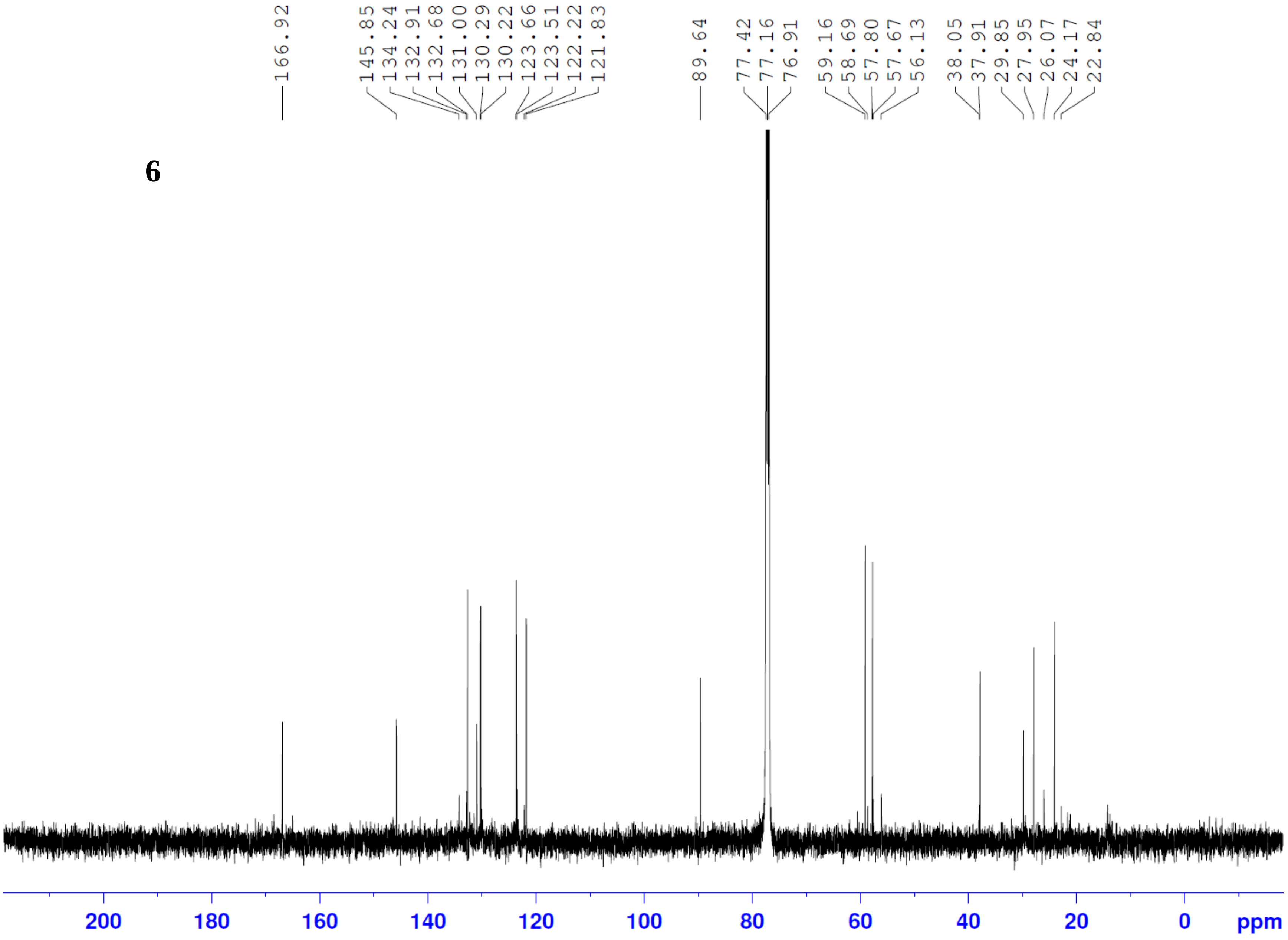
166.92
145.85
134.24
132.91
132.68
131.00
130.29
130.22
123.66
123.51
122.22
121.83
89.64
77.42
77.16
76.91
59.16
58.69
57.80
57.67
56.13
38.05
37.91
29.85
27.95
26.07
24.17
22.84
200
180
160
140
120
100
80
60
40
20
0
ppm

7

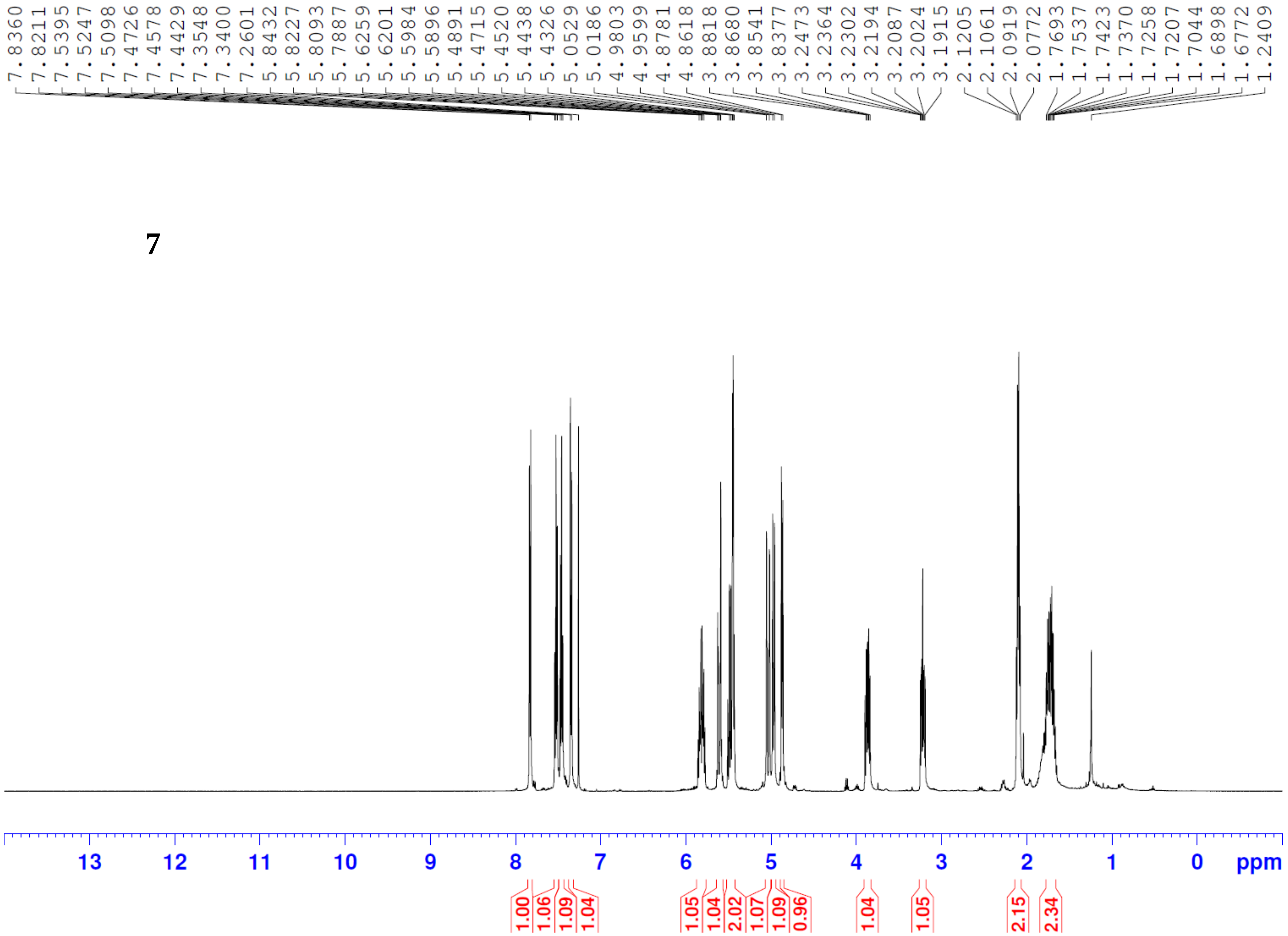

7.8360
7.8211
7.5395
7.5247
7.5098
7.4726
7.4578
7.4429
7.3548
7.3400
7.2601
5.8432
5.8227
5.8093
5.7887
5.6259
5.6201
5.5984
5.5896
5.4891
5.4715
5.4520
5.4438
5.4326
5.0529
5.0186
4.9803
4.9599
4.8781
4.8618
3.8818
3.8680
3.8541
3.8377
3.2473
3.2364
3.2302
3.2194
3.2087
3.2024
3.1915
2.1205
2.1061
2.0919
2.0772
1.7693
1.7537
1.7423
1.7370
1.7258
1.7207
1.7044
1.6898
1.6772
1.2409
13 12 11 10 9 8 7 6 5 4 3 2 1 0 ppm
1.00
1.06
1.09
1.04
1.05
1.04
2.02
1.07
1.09
0.96
1.04
1.05
2.15
2.34

7

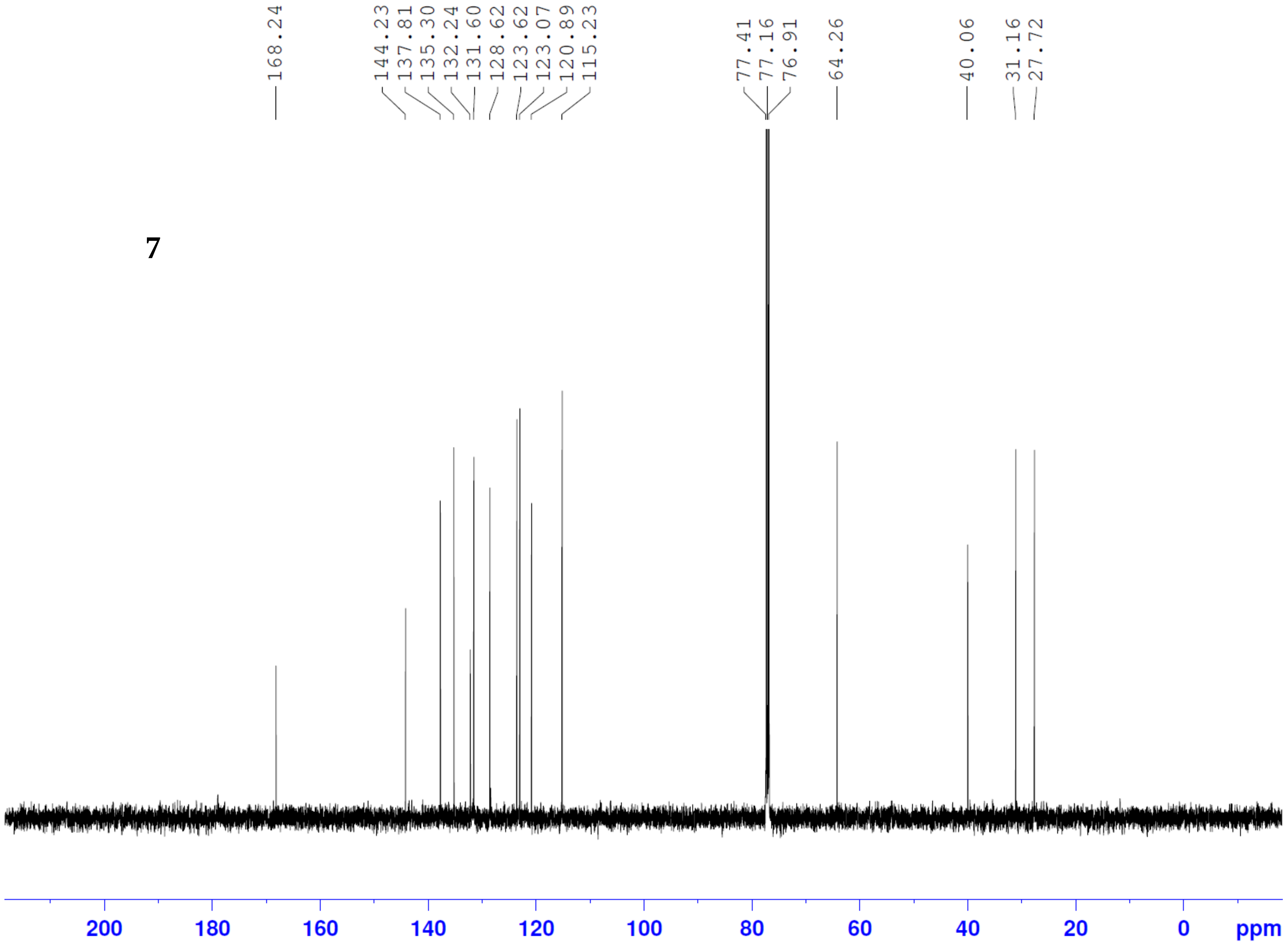
168.24
144.23
137.81
135.30
132.24
131.60
128.62
123.62
123.07
120.89
115.23
77.41
77.16
76.91
64.26
40.06
31.16
27.72
200
180
160
140
120
100
80
60
40
20
0
ppm

9{*1*}

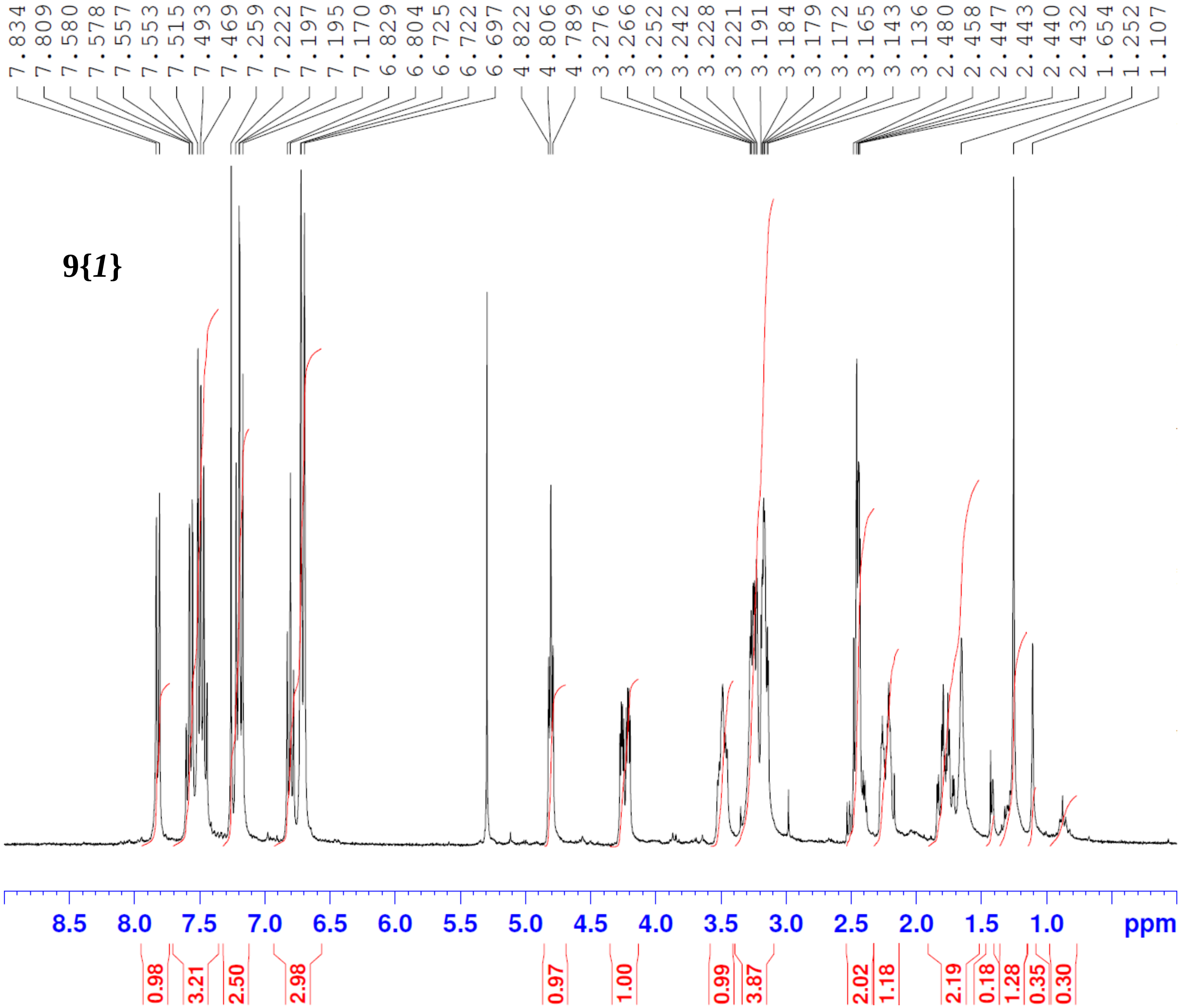
7.834
7.809
7.580
7.578
7.557
7.553
7.515
7.493
7.469
7.259
7.222
7.197
7.195
7.170
6.829
6.804
6.725
6.722
6.697
4.822
4.806
4.789
3.276
3.266
3.252
3.242
3.228
3.221
3.191
3.184
3.179
3.172
3.165
3.143
3.136
2.480
2.458
2.447
2.443
2.440
2.432
1.654
1.252
1.107
8.5 8.0 7.5 7.0 6.5 6.0 5.5 5.0 4.5 4.0 3.5 3.0 2.5 2.0 1.5 1.0 ppm
0.98 3.21 2.50 2.98 0.97 1.00 0.99 3.87 2.02 1.18 2.19 0.18 1.28 0.35 0.30

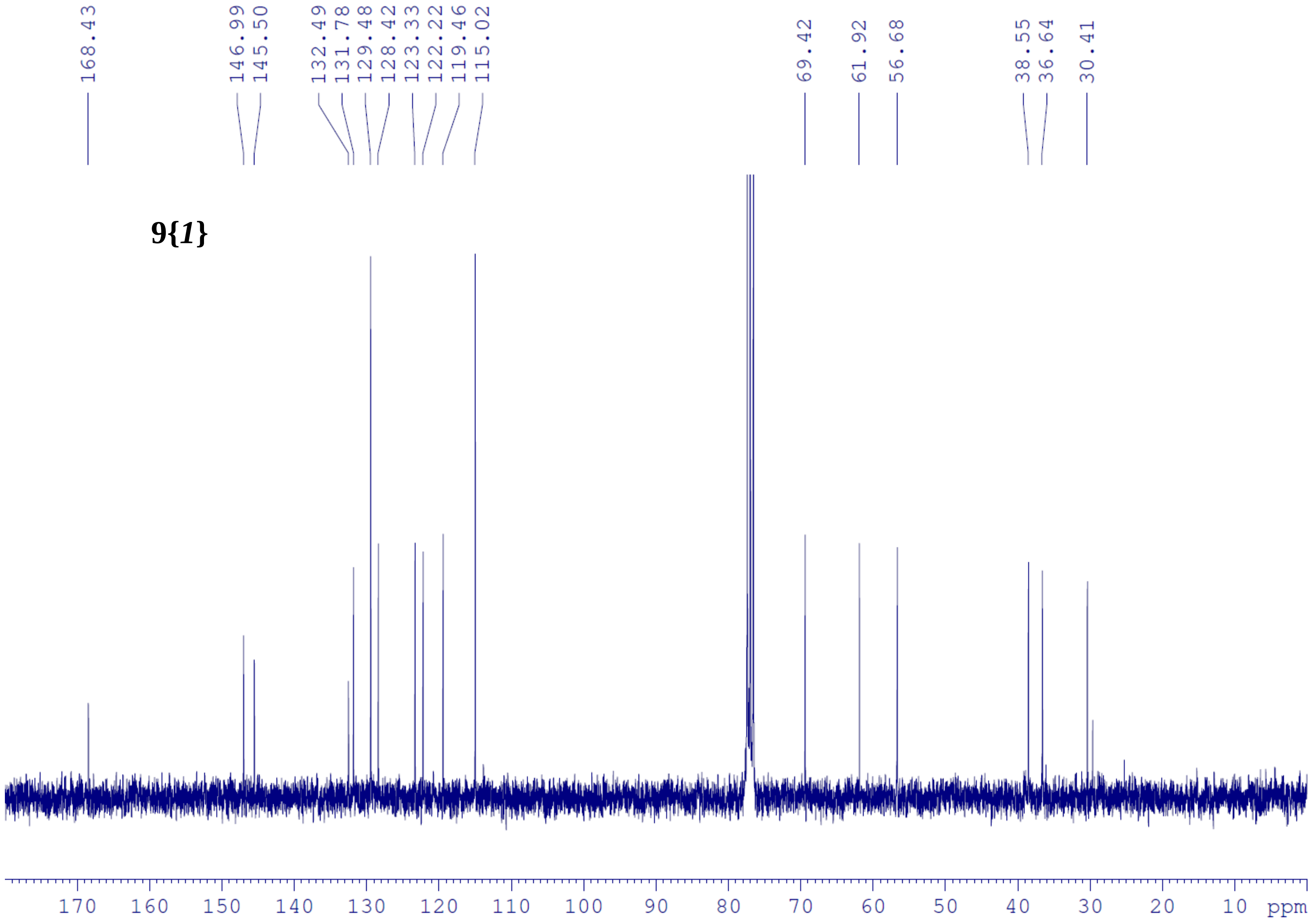
168.43
146.99
145.50
132.49
131.78
129.48
128.42
123.33
122.22
119.46
115.02
69.42
61.92
56.68
38.55
36.64
30.41
9{1}
170
160
150
140
130
120
110
100
90
80
70
60
50
40
30
20
10
ppm

10{*1*}

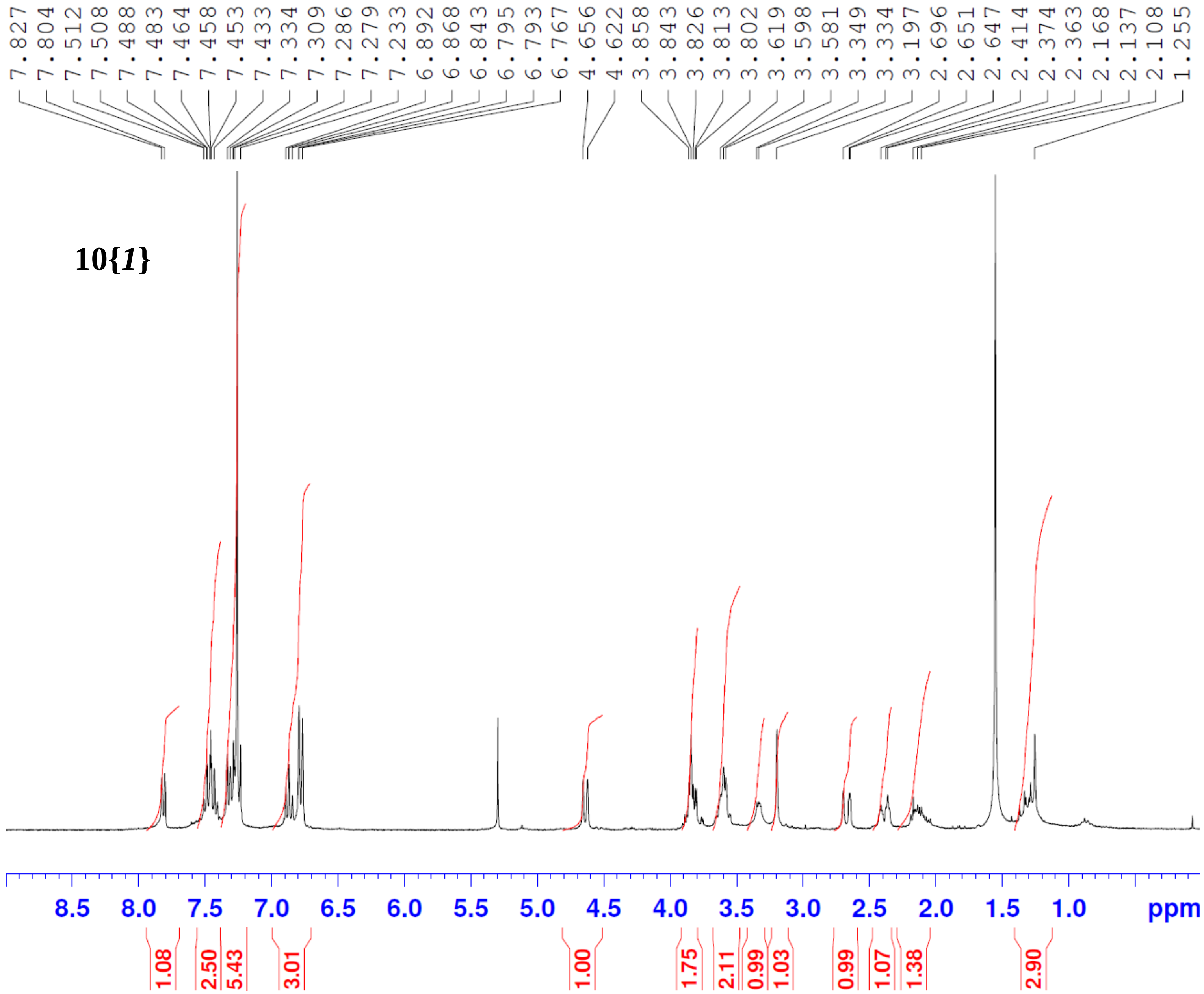
7.827
7.804
7.512
7.508
7.488
7.483
7.464
7.458
7.453
7.433
7.334
7.309
7.286
7.279
7.233
6.892
6.868
6.843
6.795
6.793
6.767
4.656
4.622
3.858
3.843
3.826
3.813
3.802
3.619
3.598
3.581
3.349
3.334
3.197
2.696
2.651
2.647
2.414
2.374
2.363
2.168
2.137
2.108
1.255
8.5
8.0
7.5
7.0
6.5
6.0
5.5
5.0
4.5
4.0
3.5
3.0
2.5
2.0
1.5
1.0
ppm
1.08
2.50
5.43
3.01
1.00
1.75
2.11
0.99
1.03
0.99
1.07
1.38
2.90

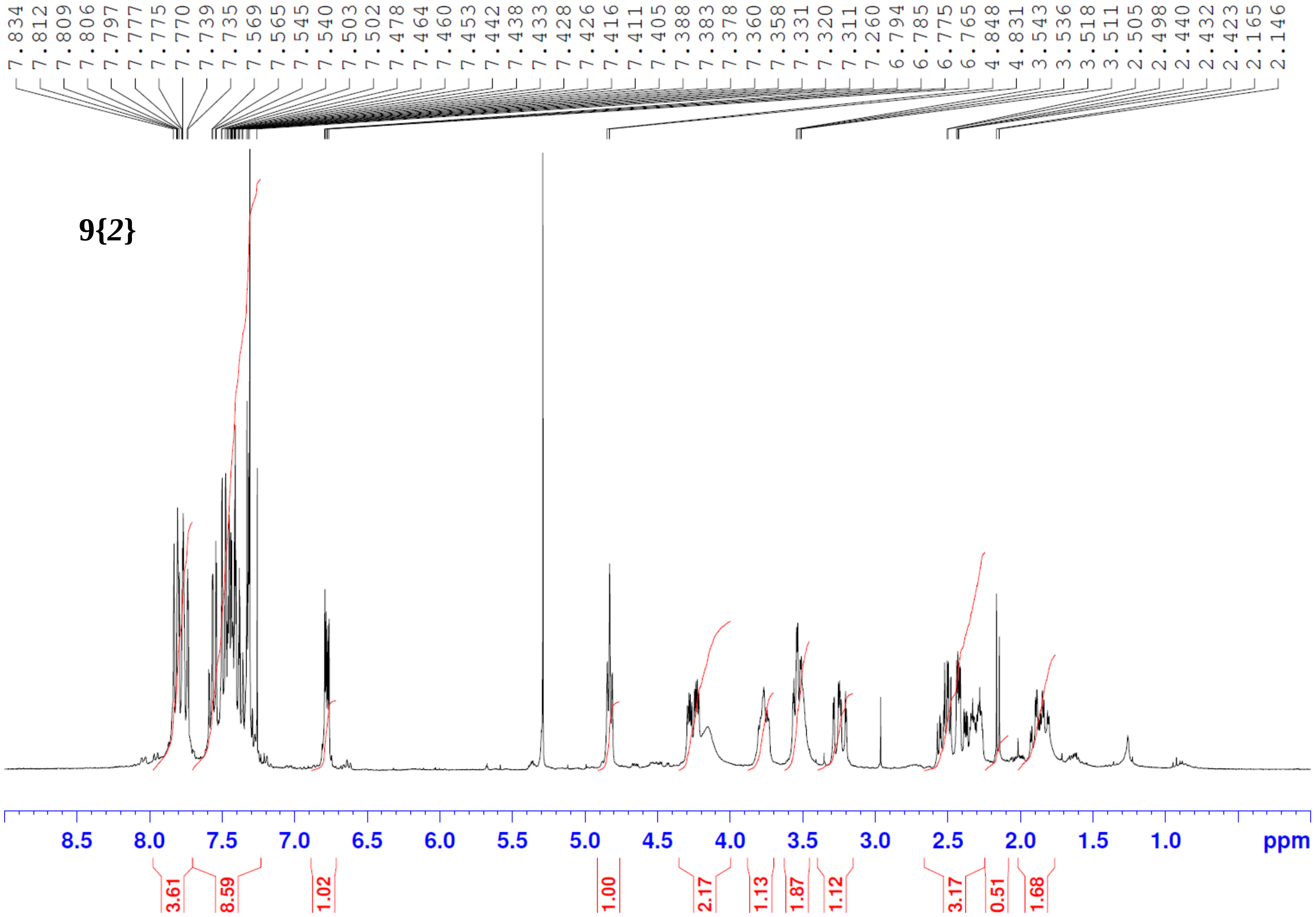
7.834 7.812 7.809 7.806 7.797 7.777 7.775 7.770 7.739 7.735 7.569 7.565 7.545 7.540 7.503 7.502 7.478 7.464 7.460 7.453 7.442 7.438 7.433 7.428 7.426 7.416 7.411 7.405 7.388 7.383 7.378 7.360 7.358 7.331 7.320 7.311 7.260 6.794 6.785 6.775 6.765 4.848 4.831 3.543 3.536 3.518 3.511 2.505 2.498 2.440 2.432 2.423 2.165 2.146
9{2}
8.5 8.0 7.5 7.0 6.5 6.0 5.5 5.0 4.5 4.0 3.5 3.0 2.5 2.0 1.5 1.0 ppm
3.61 8.59 1.02 1.00 2.17 1.13 1.87 1.12 3.17 0.51 1.68

9{2}

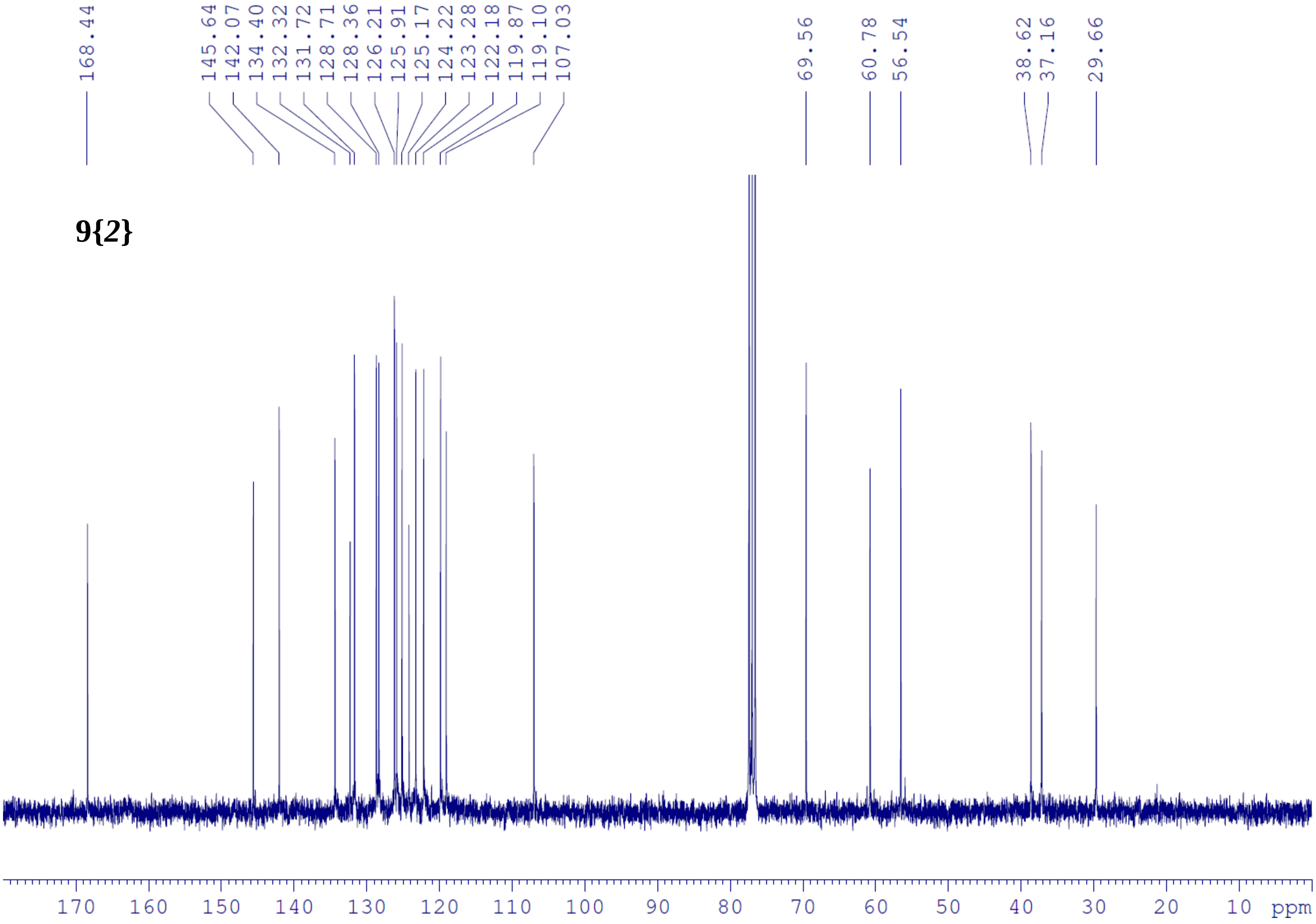

168.44
145.64
142.07
134.40
132.32
131.72
128.71
128.36
126.21
125.91
125.17
124.22
123.28
122.18
119.87
119.10
107.03
69.56
60.78
56.54
38.62
37.16
29.66
170 160 150 140 130 120 110 100 90 80 70 60 50 40 30 20 10 ppm

10{*2*}

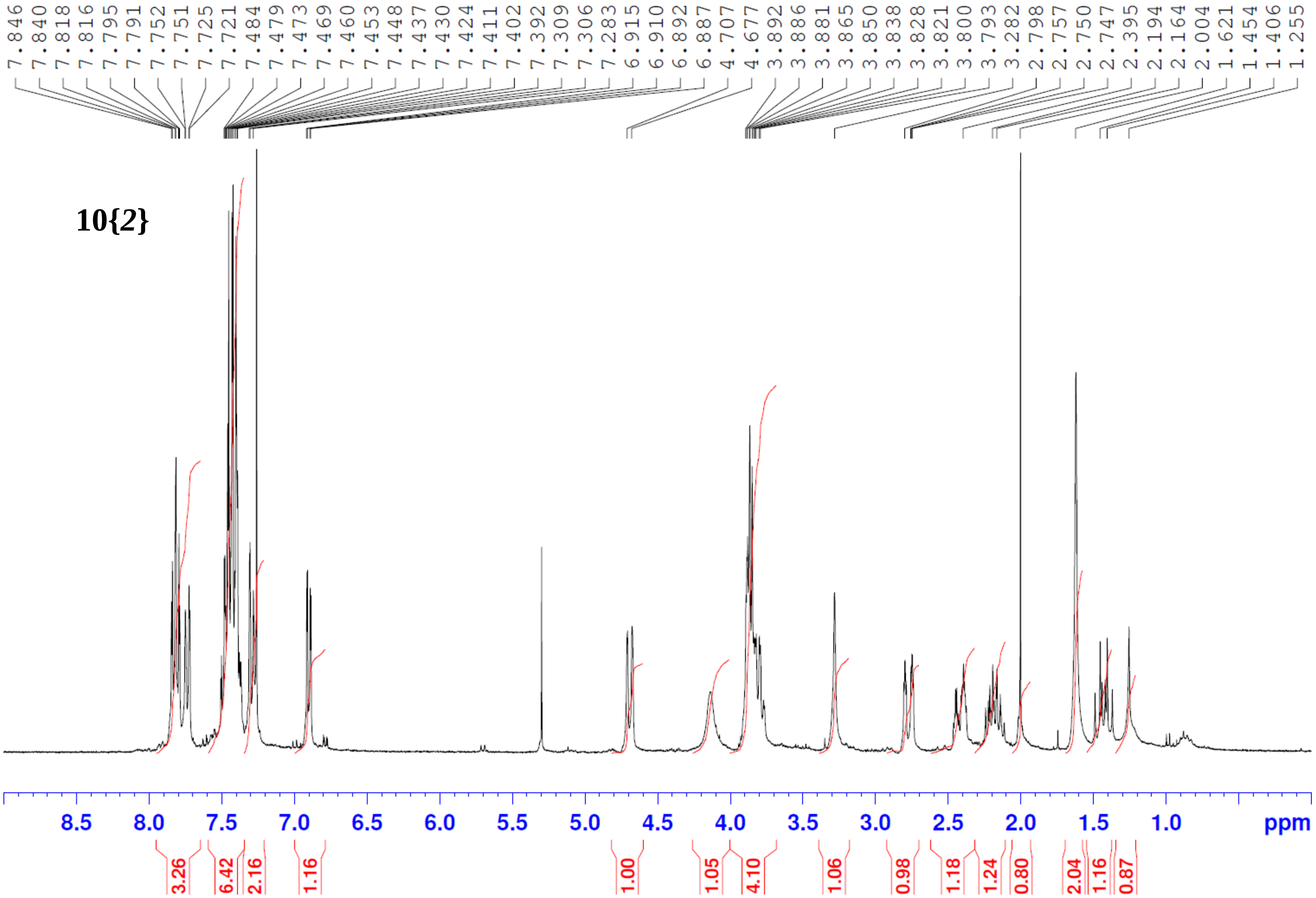

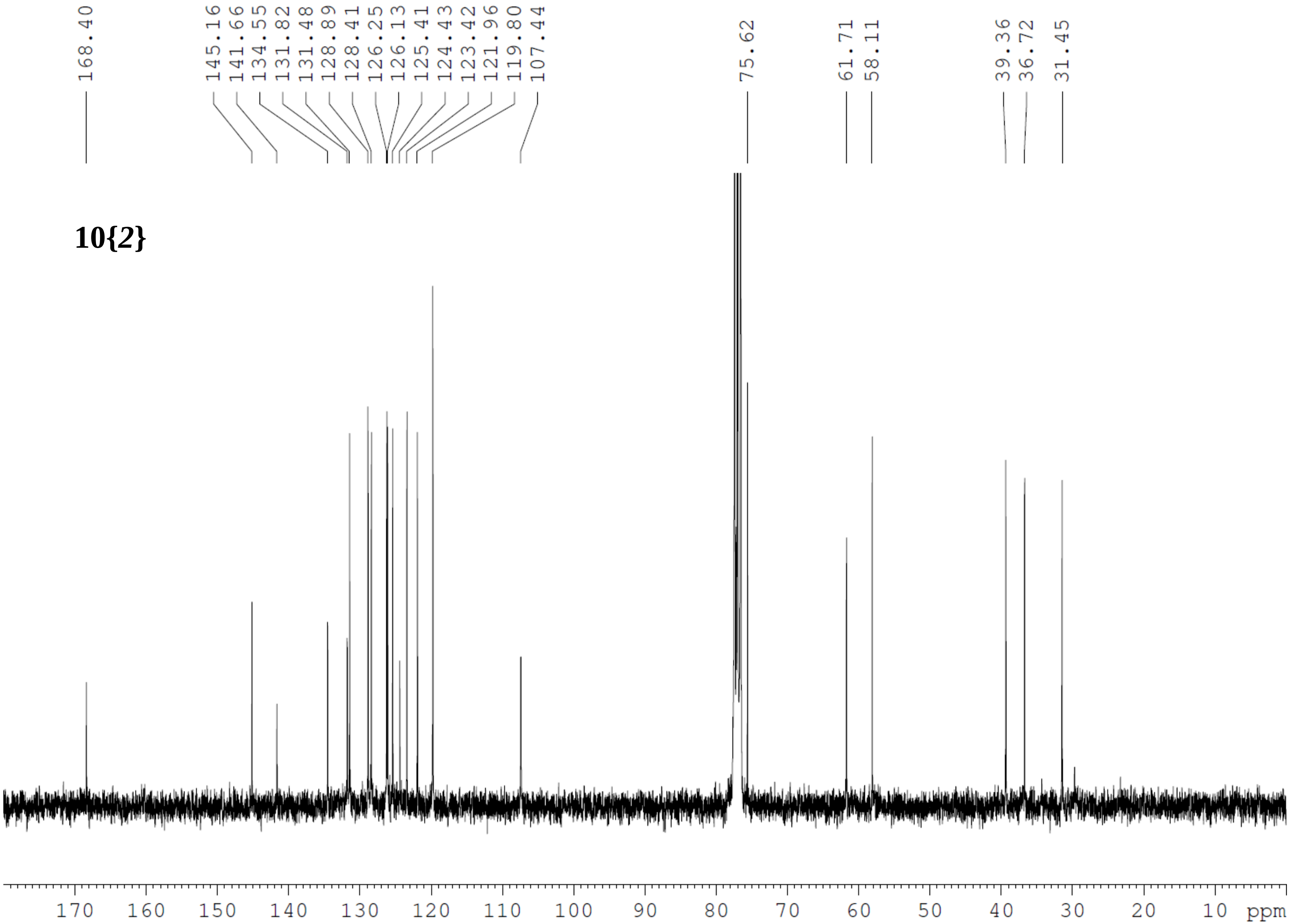
168.40
145.16
141.66
134.55
131.82
131.48
128.89
128.41
126.25
126.13
125.41
124.43
123.42
121.96
119.80
107.44
75.62
61.71
58.11
39.36
36.72
31.45
170
160
150
140
130
120
110
100
90
80
70
60
50
40
30
20
10
ppm

10{*2*}

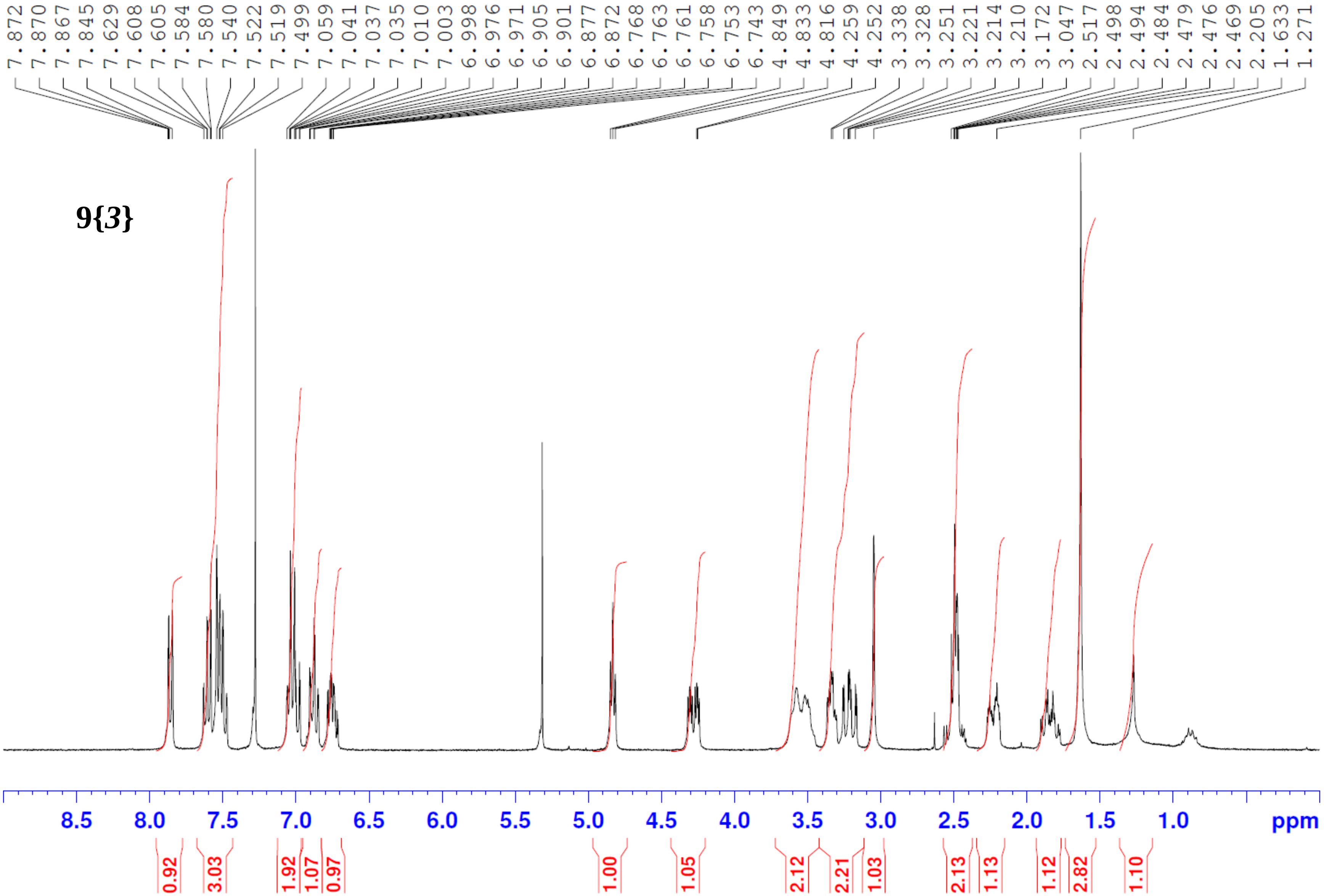
9{3}
7.872
7.870
7.867
7.845
7.629
7.608
7.605
7.584
7.580
7.540
7.522
7.519
7.499
7.059
7.041
7.037
7.035
7.010
7.003
6.998
6.976
6.971
6.905
6.901
6.877
6.872
6.768
6.763
6.761
6.758
6.753
6.743
4.849
4.833
4.816
4.259
4.252
3.338
3.328
3.251
3.221
3.214
3.210
3.172
3.047
2.517
2.498
2.494
2.484
2.479
2.476
2.469
2.205
1.633
1.271
8.5
8.0
7.5
7.0
6.5
6.0
5.5
5.0
4.5
4.0
3.5
3.0
2.5
2.0
1.5
1.0
ppm
0.92
3.03
1.92
1.07
0.97
1.00
1.05
2.12
2.21
1.03
2.13
1.13
1.12
2.82
1.10

10{*3*}

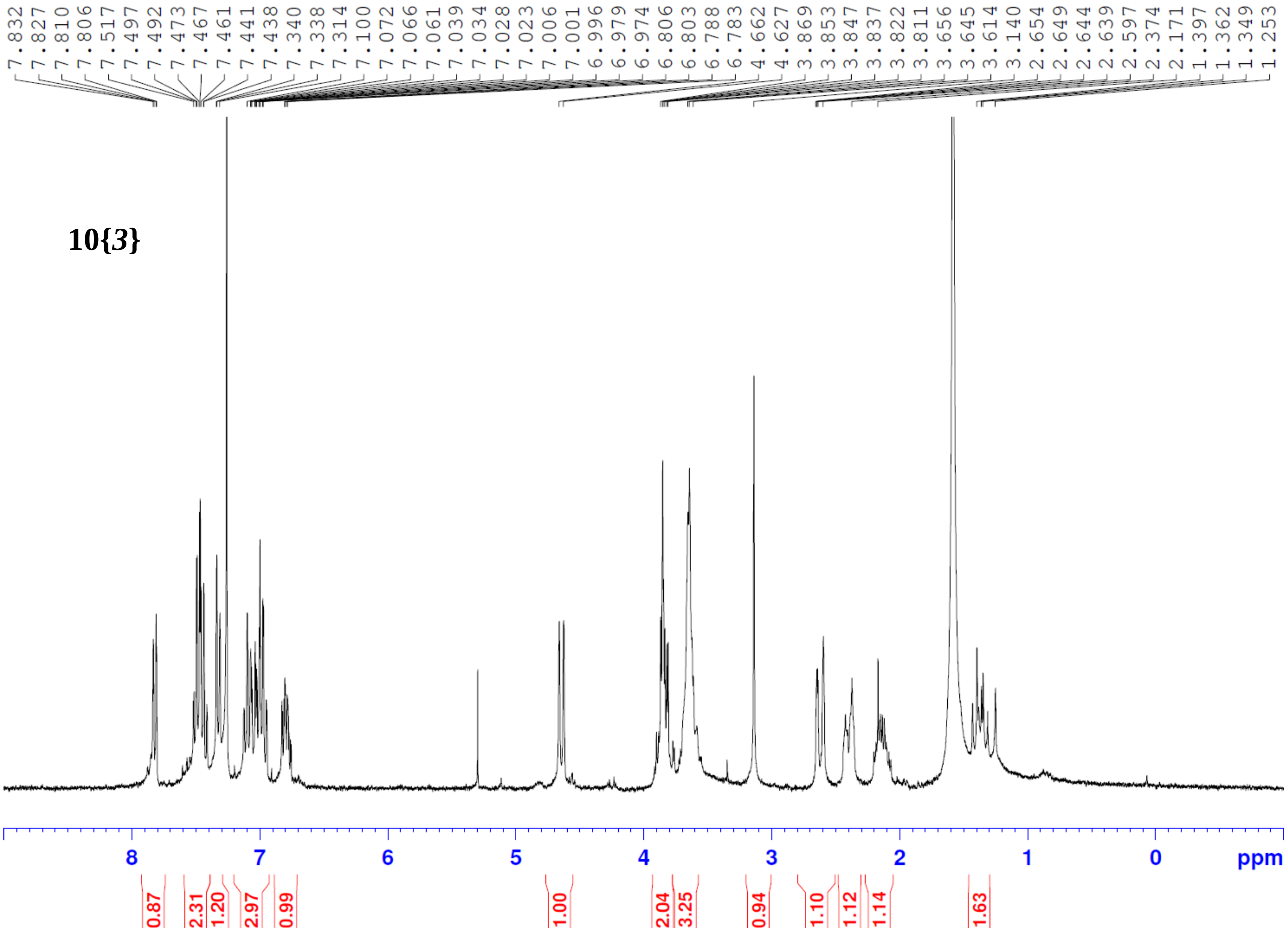
7.832
7.827
7.810
7.806
7.517
7.497
7.492
7.473
7.467
7.461
7.441
7.438
7.340
7.338
7.314
7.100
7.072
7.066
7.061
7.039
7.034
7.028
7.023
7.006
7.001
6.996
6.979
6.974
6.806
6.803
6.788
6.783
4.662
4.627
3.869
3.853
3.847
3.837
3.822
3.811
3.656
3.645
3.614
3.140
2.654
2.649
2.644
2.639
2.597
2.374
2.171
1.397
1.362
1.349
1.253
8
7
6
5
4
3
2
1
0
ppm
0.87
2.31
1.20
2.97
0.99
1.00
2.04
3.25
0.94
1.10
1.12
1.14
1.63

9{*4*}

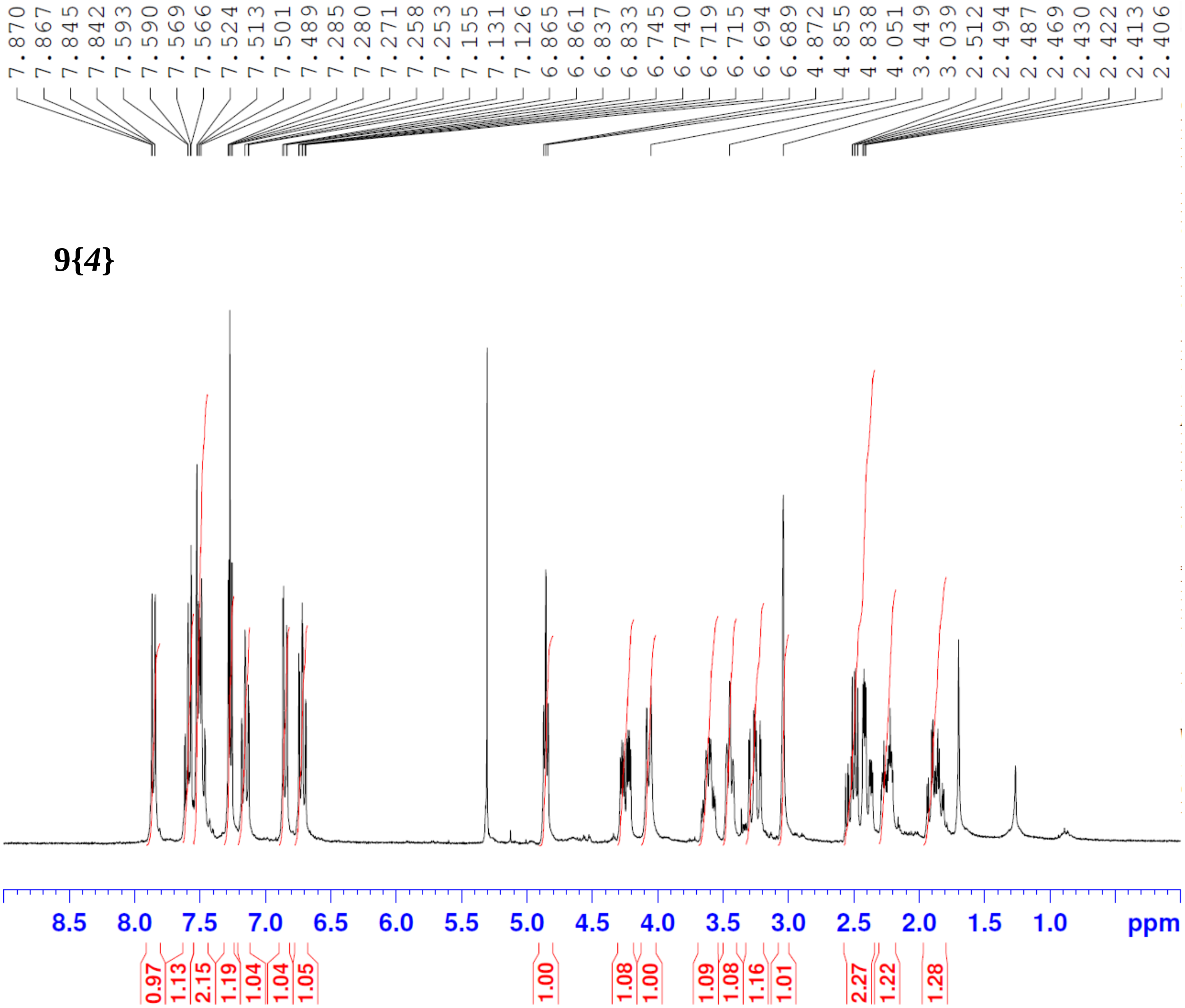
7.870 7.867 7.845 7.842 7.593 7.590 7.569 7.566 7.524 7.513 7.501 7.489 7.285 7.280 7.271 7.258 7.253 7.155 7.131 7.126 6.865 6.861 6.837 6.833 6.745 6.740 6.719 6.715 6.694 6.689 4.872 4.855 4.838 4.051 3.449 3.039 2.512 2.494 2.487 2.469 2.430 2.422 2.413 2.406
8.5 8.0 7.5 7.0 6.5 6.0 5.5 5.0 4.5 4.0 3.5 3.0 2.5 2.0 1.5 1.0 ppm
0.97 1.13 2.15 1.19 1.04 1.04 1.05 1.00 1.08 1.00 1.09 1.08 1.16 1.01 2.27 1.22 1.28

9{*4*}

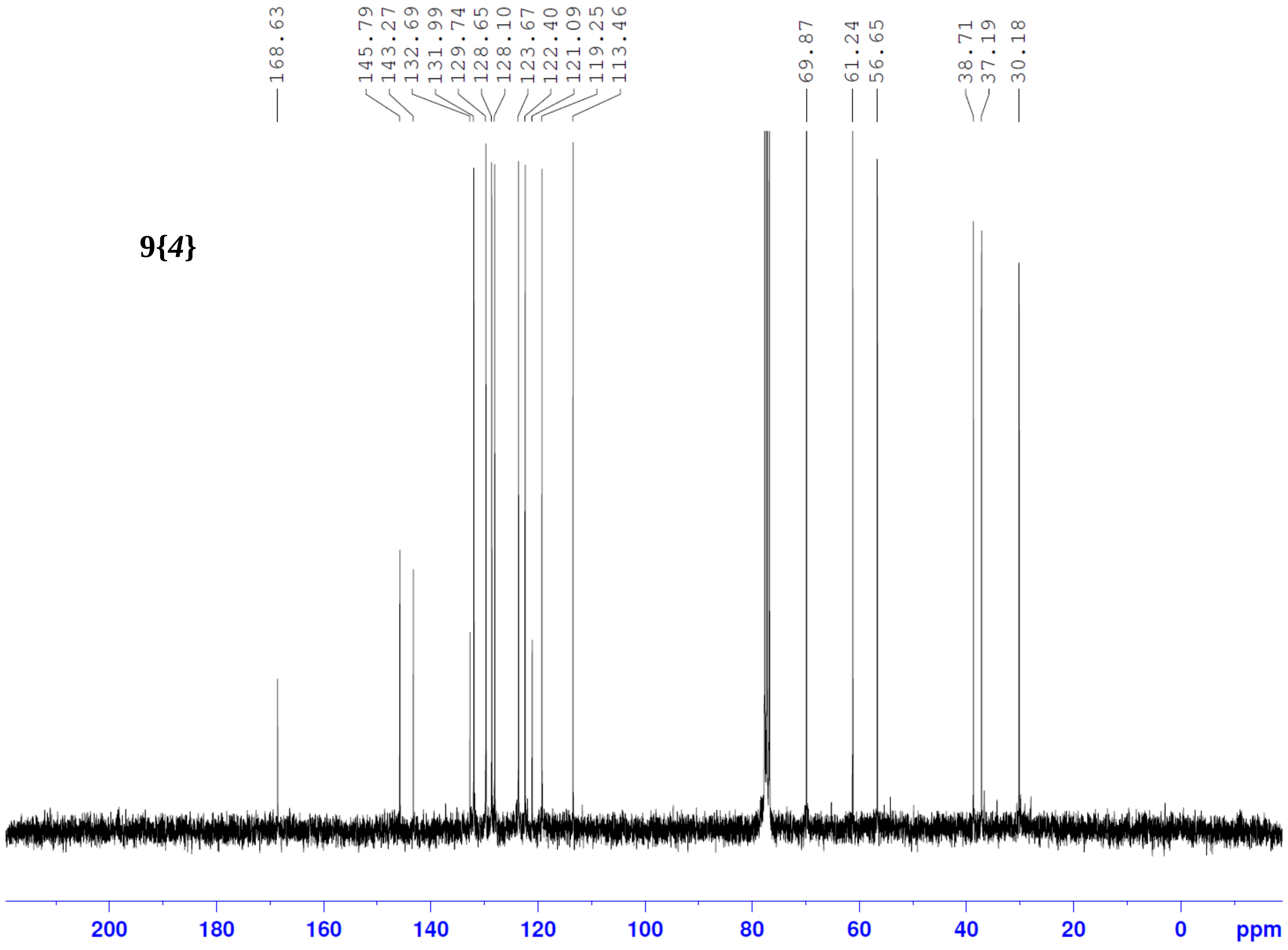
168.63
145.79
143.27
132.69
131.99
129.74
128.65
128.10
123.67
122.40
121.09
119.25
113.46
69.87
61.24
56.65
38.71
37.19
30.18
200
180
160
140
120
100
80
60
40
20
0
ppm

10{*4*}

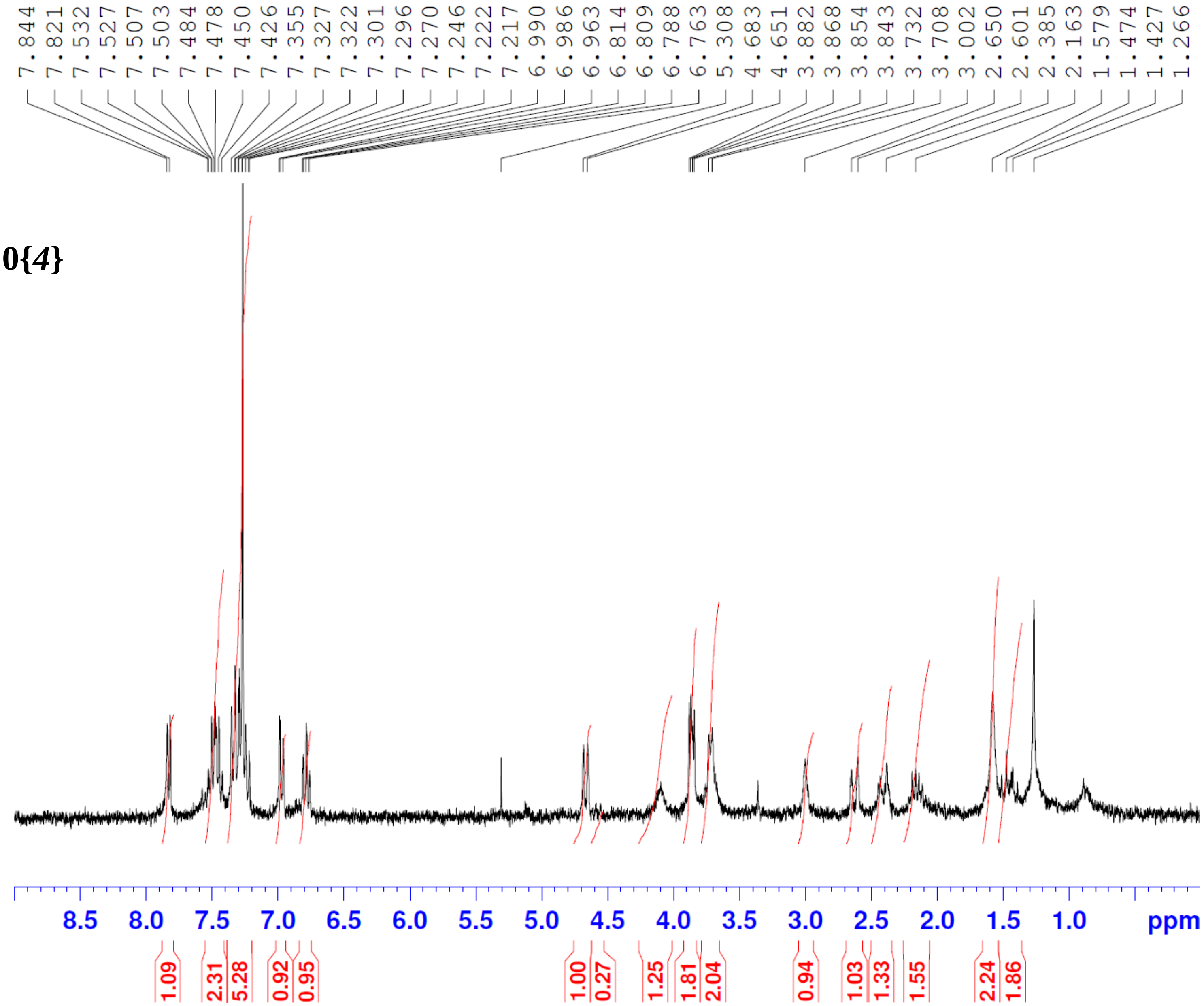
7.844
7.821
7.532
7.527
7.507
7.503
7.484
7.478
7.450
7.426
7.355
7.327
7.322
7.301
7.296
7.270
7.246
7.222
7.217
6.990
6.986
6.963
6.814
6.809
6.788
6.763
5.308
4.683
4.651
3.882
3.868
3.854
3.843
3.732
3.708
3.002
2.650
2.601
2.385
2.163
1.579
1.474
1.427
1.266
8.5
8.0
7.5
7.0
6.5
6.0
5.5
5.0
4.5
4.0
3.5
3.0
2.5
2.0
1.5
1.0
ppm
1.09
2.31
5.28
0.92
0.95
1.00
0.27
1.25
1.81
2.04
0.94
1.03
1.33
1.55
2.24
1.86

9{5}

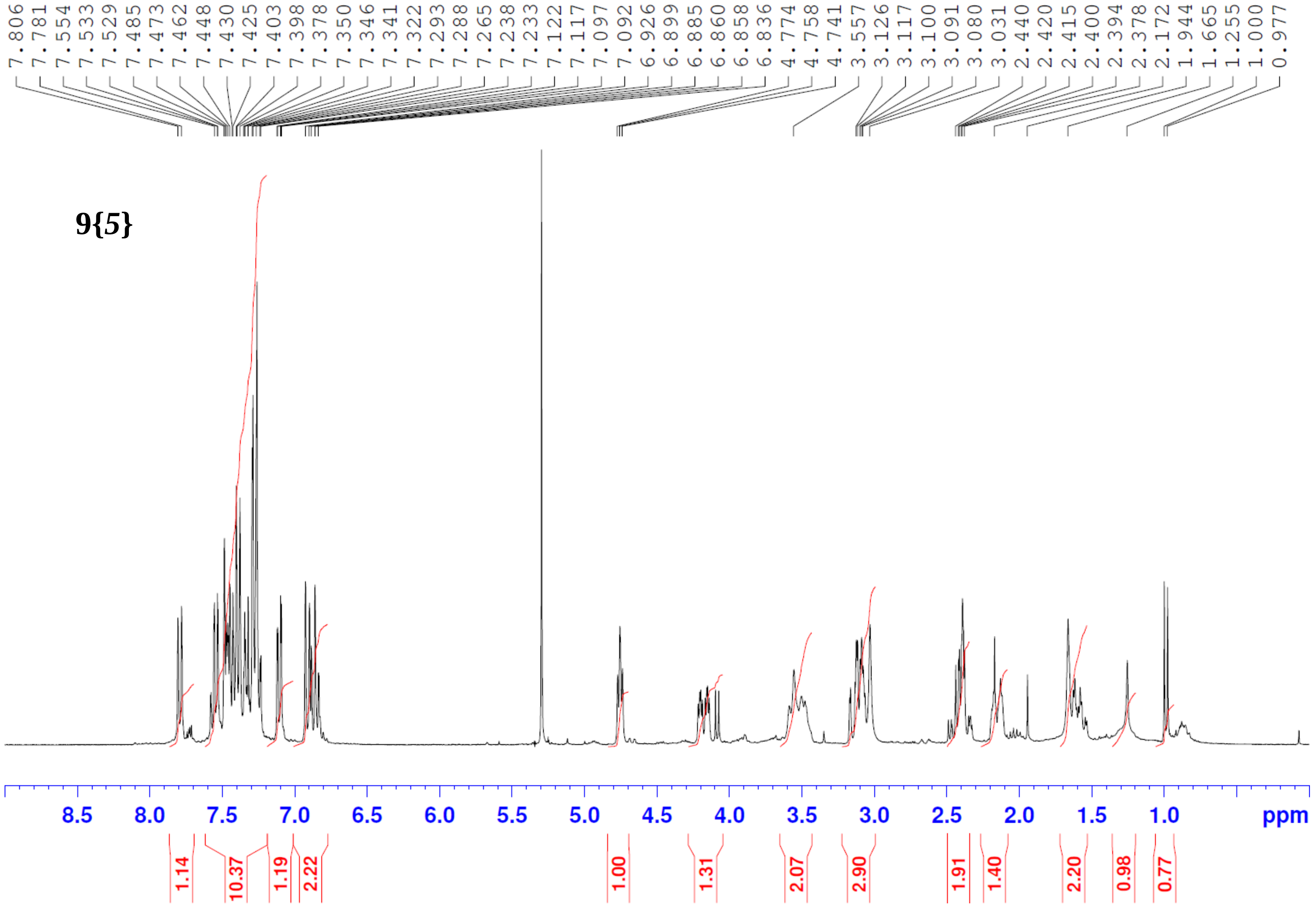
7.806
7.781
7.554
7.533
7.529
7.485
7.473
7.462
7.448
7.430
7.425
7.403
7.398
7.378
7.350
7.346
7.341
7.322
7.293
7.288
7.265
7.238
7.233
7.122
7.117
7.097
7.092
6.926
6.899
6.885
6.860
6.858
6.836
4.774
4.758
4.741
3.557
3.126
3.117
3.100
3.091
3.080
3.031
2.440
2.420
2.415
2.400
2.394
2.378
2.172
1.944
1.665
1.255
1.000
0.977
8.5
8.0
7.5
7.0
6.5
6.0
5.5
5.0
4.5
4.0
3.5
3.0
2.5
2.0
1.5
1.0
ppm
1.14
10.37
1.19
2.22
1.00
1.31
2.07
2.90
1.91
1.40
2.20
0.98
0.77

10{*5*}

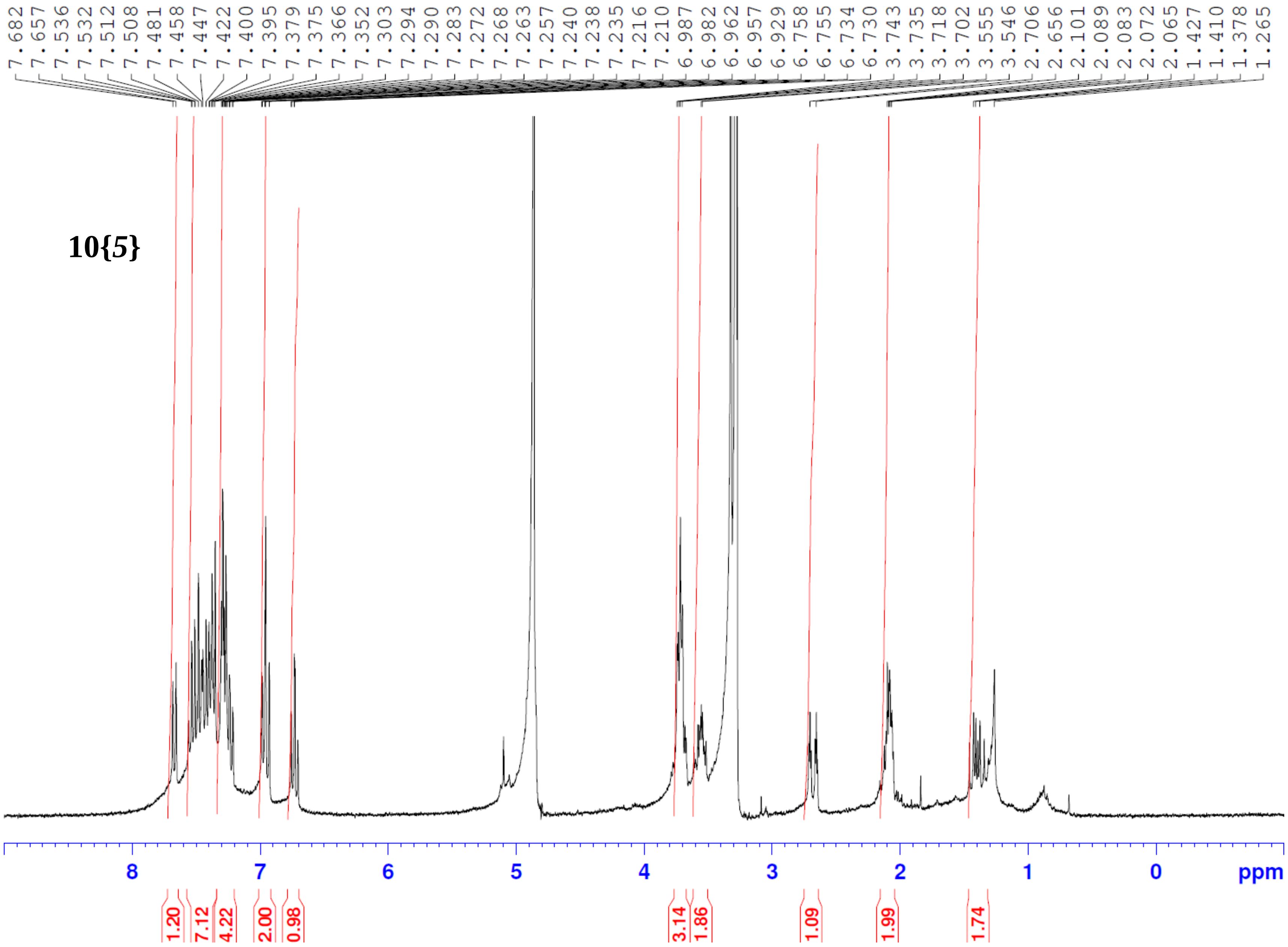
7.682
7.657
7.536
7.532
7.512
7.508
7.481
7.458
7.447
7.422
7.400
7.395
7.379
7.375
7.366
7.352
7.303
7.294
7.290
7.283
7.272
7.268
7.263
7.257
7.240
7.238
7.235
7.216
7.210
6.987
6.982
6.962
6.957
6.929
6.758
6.755
6.734
6.730
3.743
3.735
3.718
3.702
3.555
3.546
2.706
2.656
2.101
2.089
2.083
2.072
2.065
1.427
1.410
1.378
1.265
8
7
6
5
4
3
2
1
0
ppm
1.20
7.12
4.22
2.00
0.98
3.14
1.86
1.09
1.99
1.74

9{*6*}

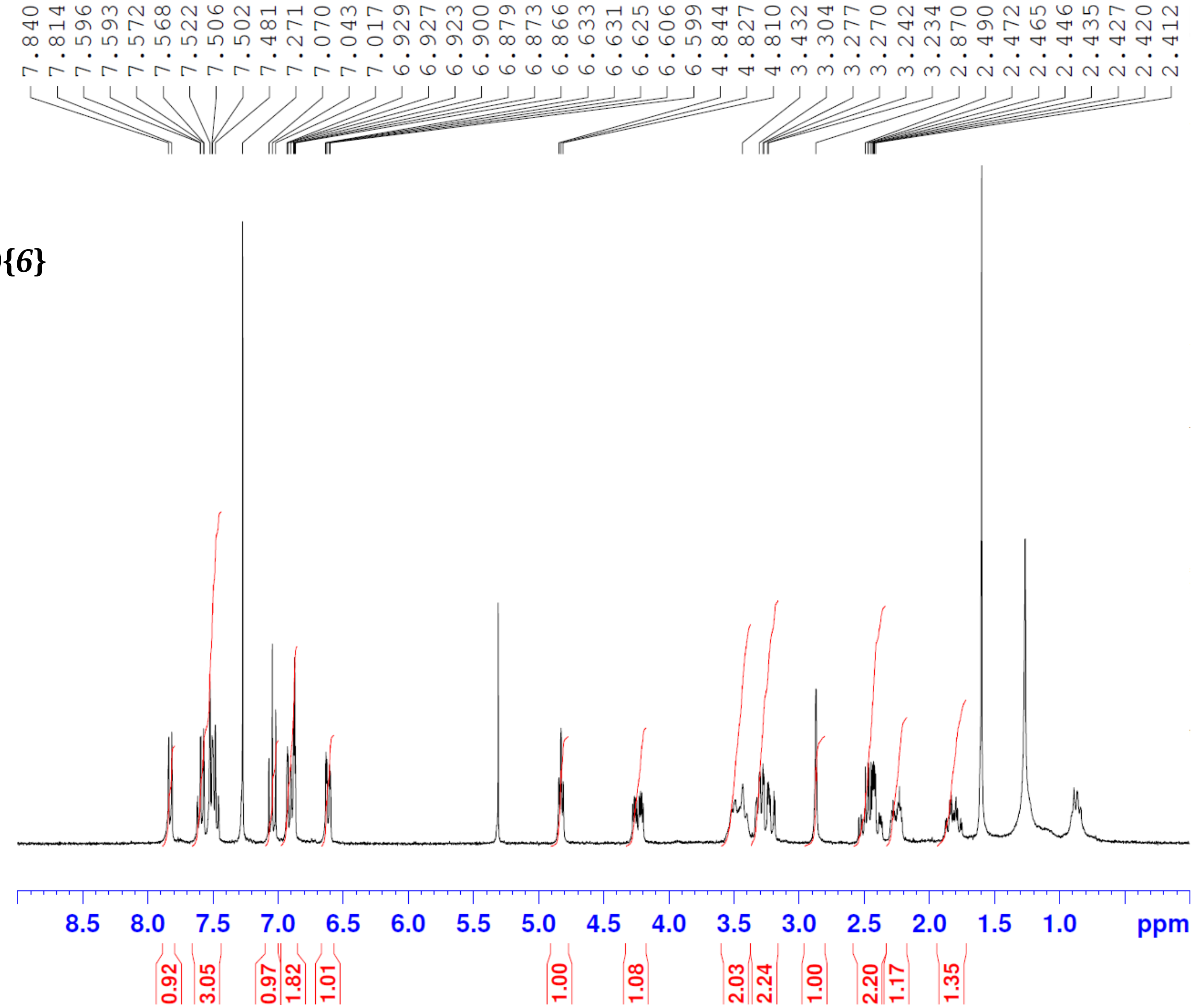

7.840 7.814 7.596 7.593 7.572 7.568 7.522 7.506 7.502 7.481 7.271 7.070 7.043 7.017 6.929 6.927 6.923 6.900 6.879 6.873 6.866 6.633 6.631 6.625 6.606 6.599 4.844 4.827 4.810 3.432 3.304 3.277 3.270 3.242 3.234 2.870 2.490 2.472 2.465 2.446 2.435 2.427 2.420 2.412
8.5 8.0 7.5 7.0 6.5 6.0 5.5 5.0 4.5 4.0 3.5 3.0 2.5 2.0 1.5 1.0 ppm
0.92 3.05 0.97 1.82 1.01 1.00 1.08 2.03 2.24 1.00 2.20 1.17 1.35

9{*6*}

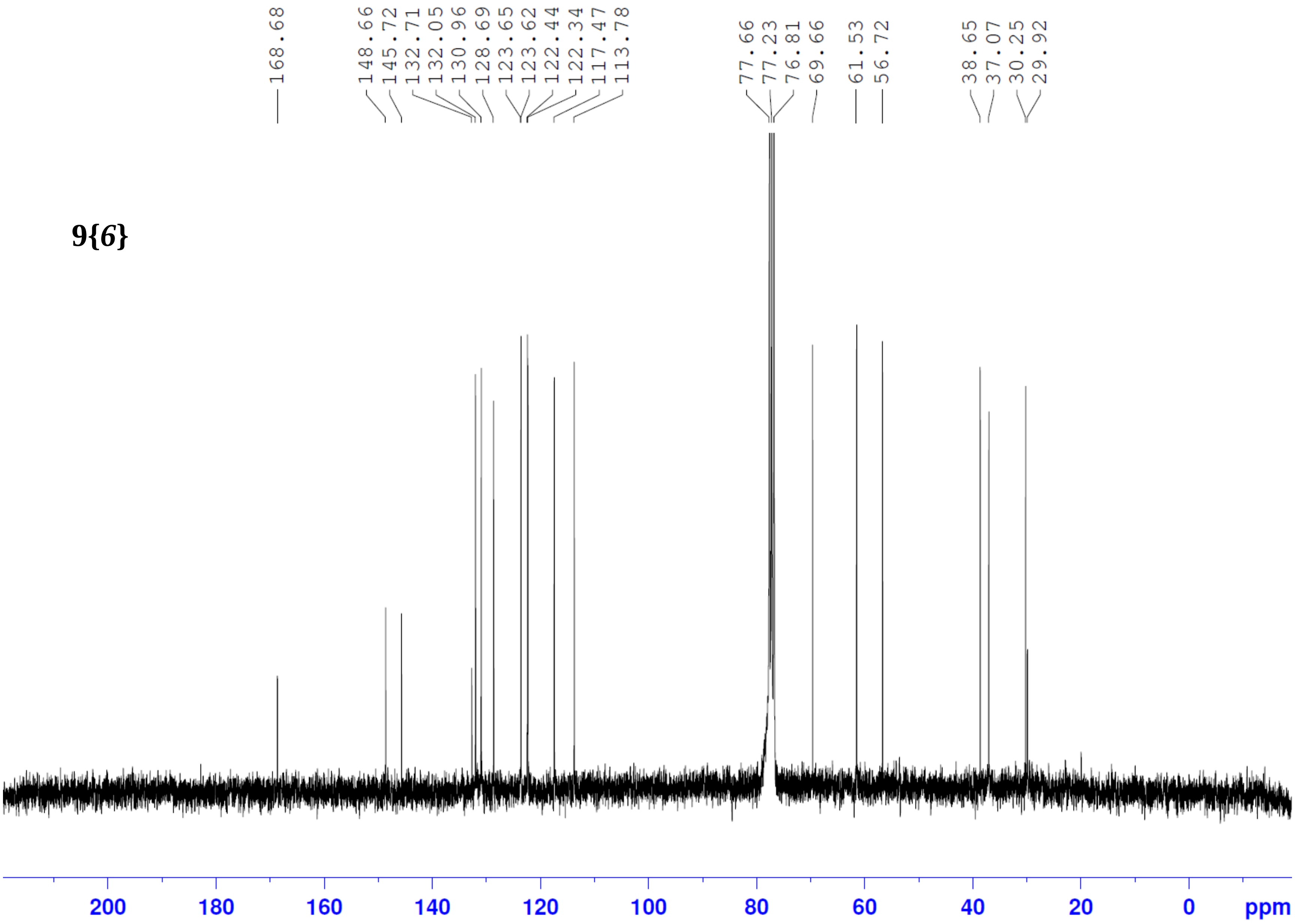
168.68
148.66
145.72
132.71
132.05
130.96
128.69
123.65
123.62
122.44
122.34
117.47
113.78
77.66
77.23
76.81
69.66
61.53
56.72
38.65
37.07
30.25
29.92
200
180
160
140
120
100
80
60
40
20
0
ppm

10{*6*}

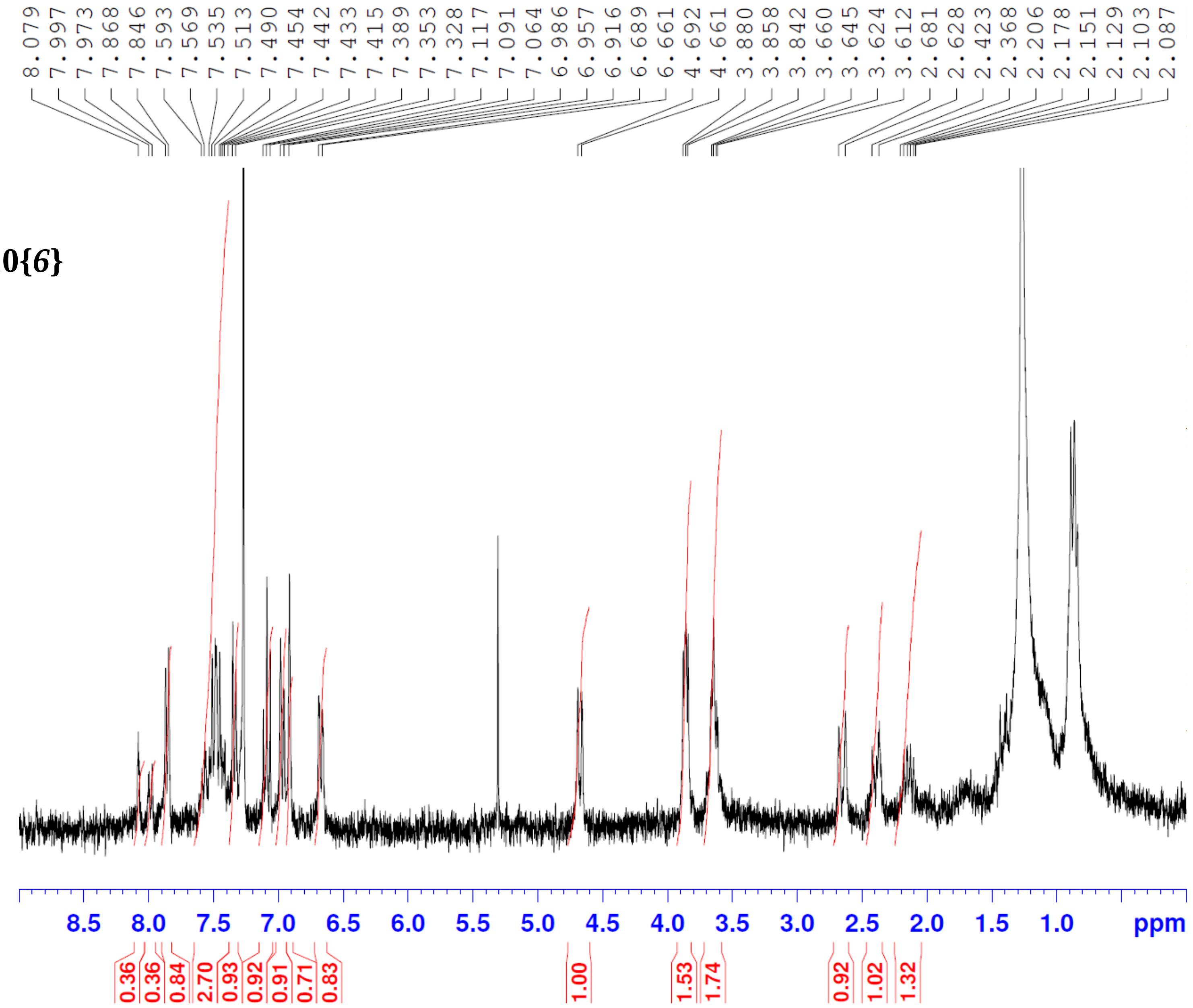
8.079
7.997
7.973
7.868
7.846
7.593
7.569
7.535
7.513
7.490
7.454
7.442
7.433
7.415
7.389
7.353
7.328
7.117
7.091
7.064
6.986
6.957
6.916
6.689
6.661
4.692
4.661
3.880
3.858
3.842
3.660
3.645
3.624
3.612
2.681
2.628
2.423
2.368
2.206
2.178
2.151
2.129
2.103
2.087
8.5 8.0 7.5 7.0 6.5 6.0 5.5 5.0 4.5 4.0 3.5 3.0 2.5 2.0 1.5 1.0 ppm
0.36 0.36 0.84 2.70 0.93 0.92 0.91 0.71 0.83 1.00 1.53 1.74 0.92 1.02 1.32

9{*7*}

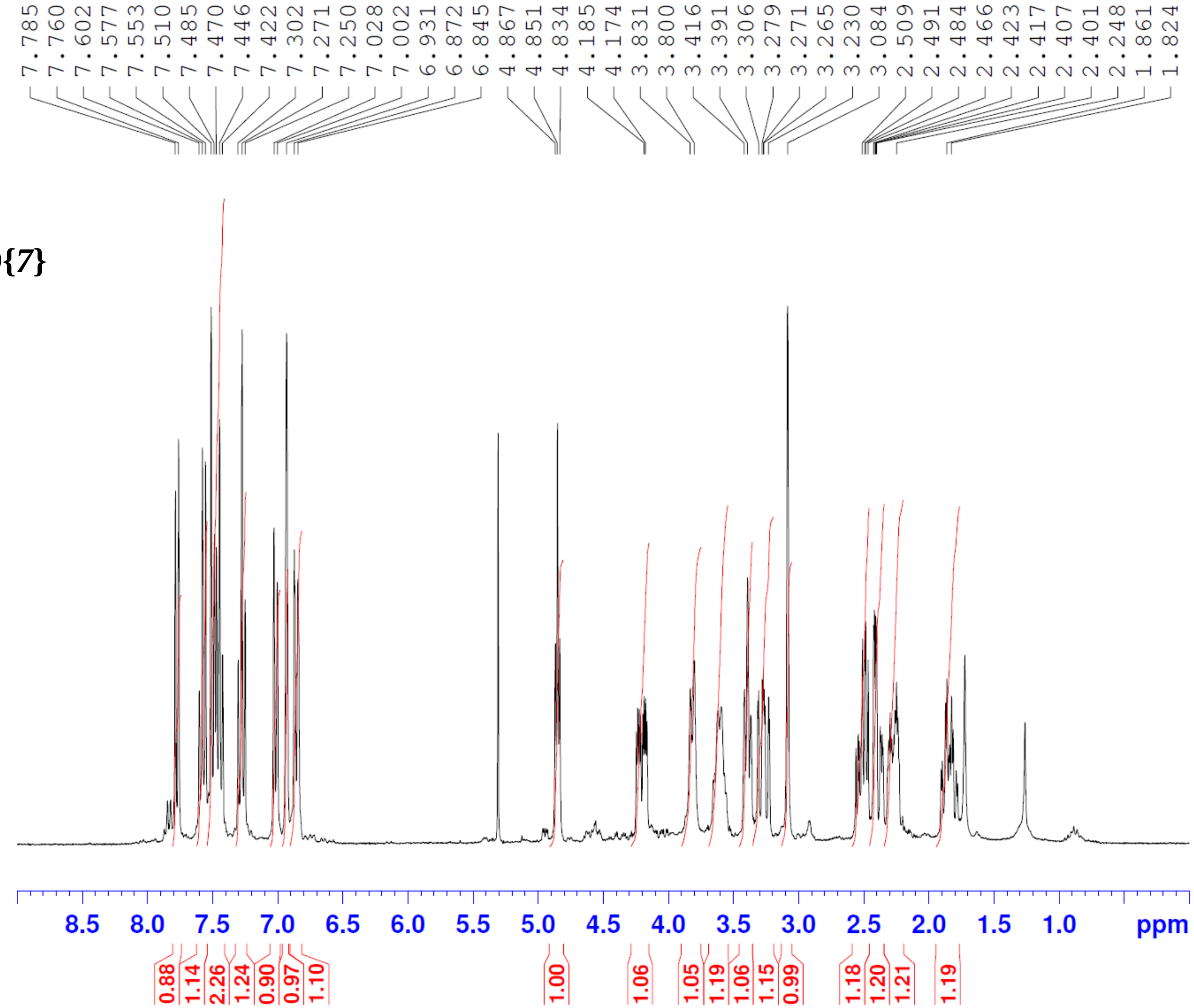

7.785
7.760
7.602
7.577
7.553
7.510
7.485
7.470
7.446
7.422
7.302
7.271
7.250
7.028
7.002
6.931
6.872
6.845
4.867
4.851
4.834
4.185
4.174
3.831
3.800
3.416
3.391
3.306
3.279
3.271
3.265
3.230
3.084
2.509
2.491
2.484
2.466
2.423
2.417
2.407
2.401
2.248
1.861
1.824
8.5
8.0
7.5
7.0
6.5
6.0
5.5
5.0
4.5
4.0
3.5
3.0
2.5
2.0
1.5
1.0
ppm
0.88
1.14
2.26
1.24
0.90
0.97
1.10
1.00
1.06
1.05
1.19
1.06
1.15
0.99
1.18
1.20
1.21
1.19

9{*7*}

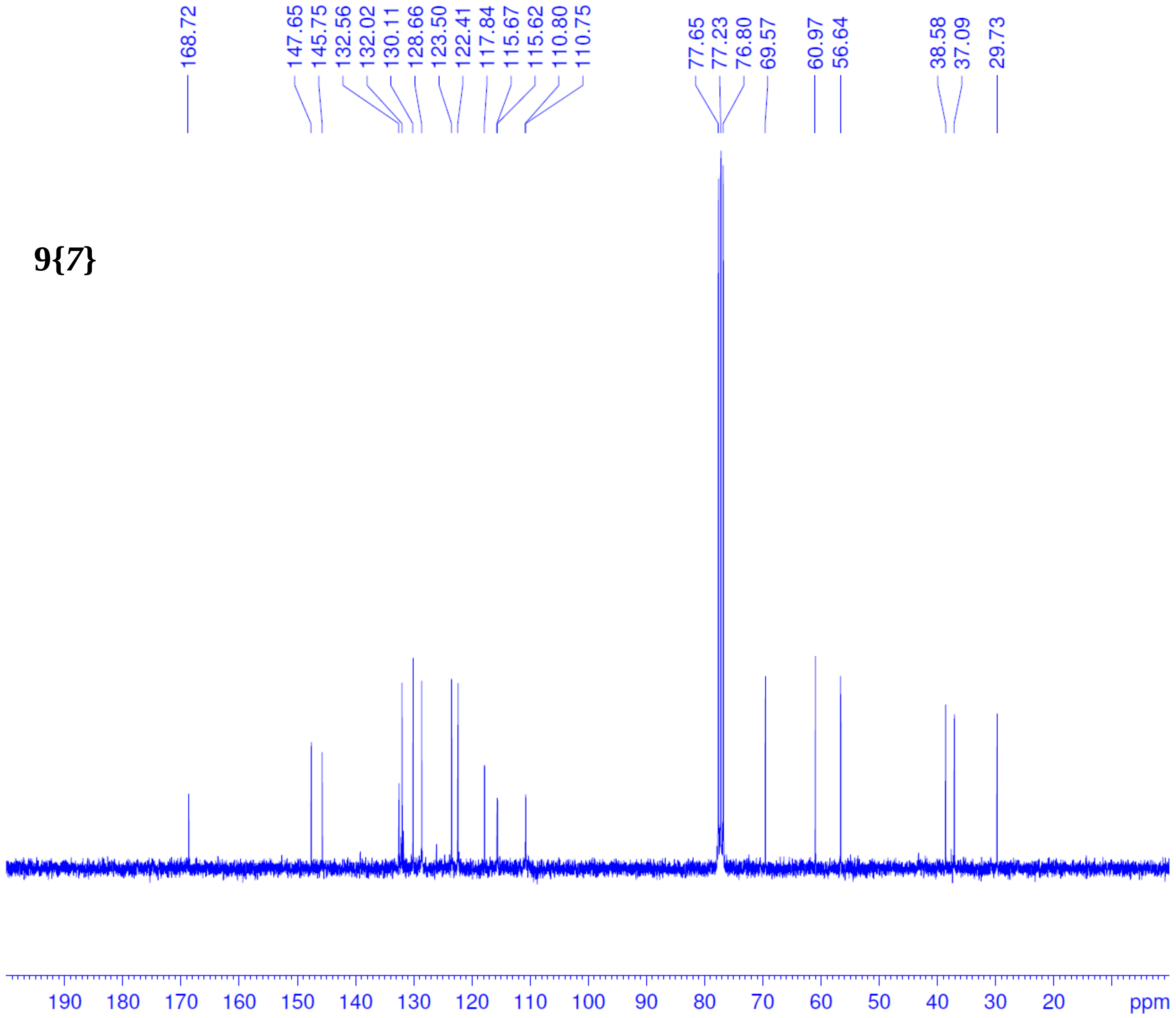

168.72
147.65
145.75
132.56
132.02
130.11
128.66
123.50
122.41
117.84
115.67
115.62
110.80
110.75
77.65
77.23
76.80
69.57
60.97
56.64
38.58
37.09
29.73
190
180
170
160
150
140
130
120
110
100
90
80
70
60
50
40
30
20
ppm

10{*7*}

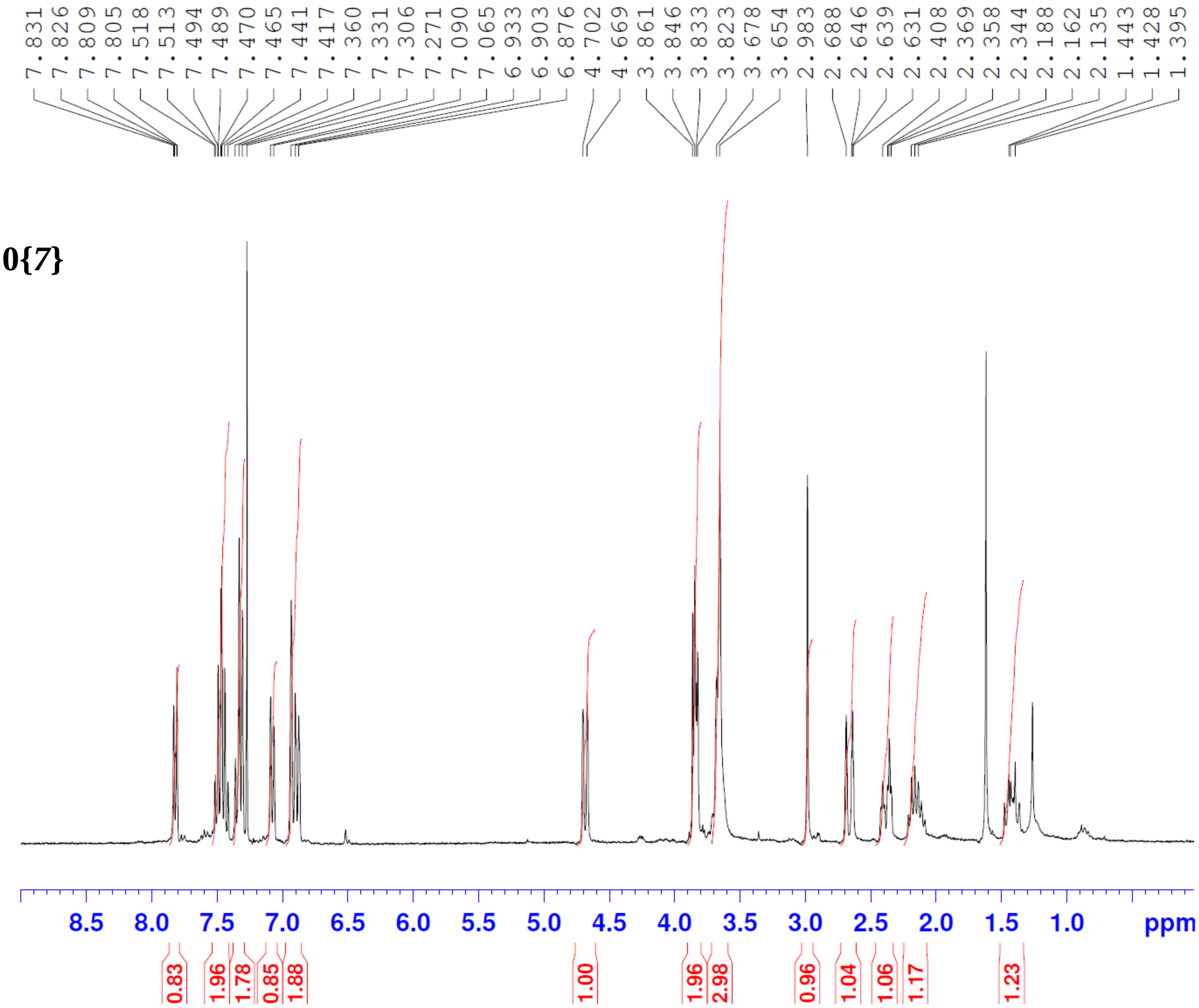
7.831
7.826
7.809
7.805
7.518
7.513
7.494
7.489
7.470
7.465
7.441
7.417
7.360
7.331
7.306
7.271
7.090
7.065
6.933
6.903
6.876
4.702
4.669
3.861
3.846
3.833
3.823
3.678
3.654
2.983
2.688
2.646
2.639
2.631
2.408
2.369
2.358
2.344
2.188
2.162
2.135
1.443
1.428
1.395
8.5
8.0
7.5
7.0
6.5
6.0
5.5
5.0
4.5
4.0
3.5
3.0
2.5
2.0
1.5
1.0
ppm
0.83
1.96
1.78
0.85
1.88
1.00
1.96
2.98
0.96
1.04
1.06
1.17
1.23

10{*7*}

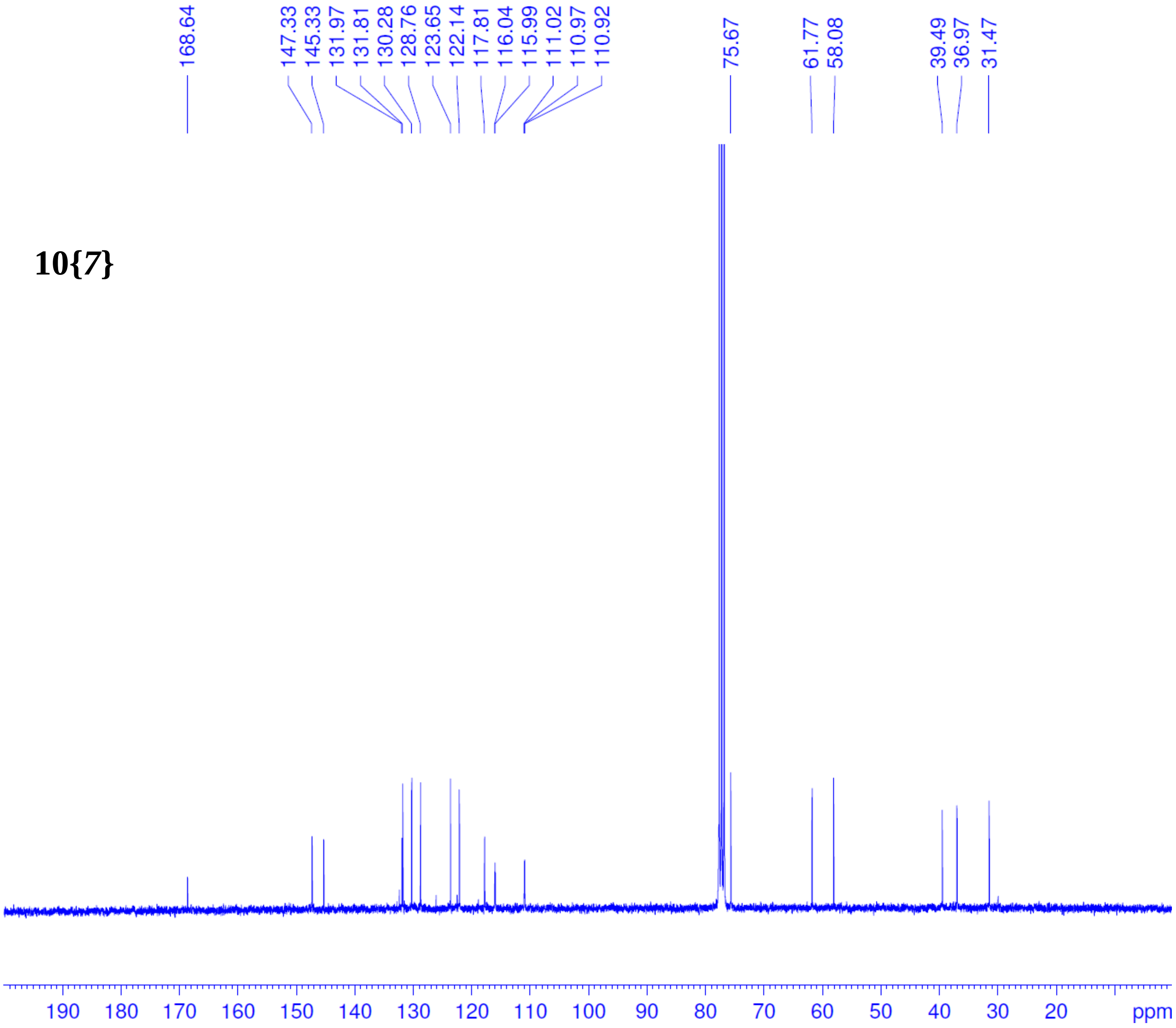
168.64
147.33
145.33
131.97
131.81
130.28
128.76
123.65
122.14
117.81
116.04
115.99
111.02
110.97
110.92
75.67
61.77
58.08
39.49
36.97
31.47
190 180 170 160 150 140 130 120 110 100 90 80 70 60 50 40 30 20 ppm

9{*8*}

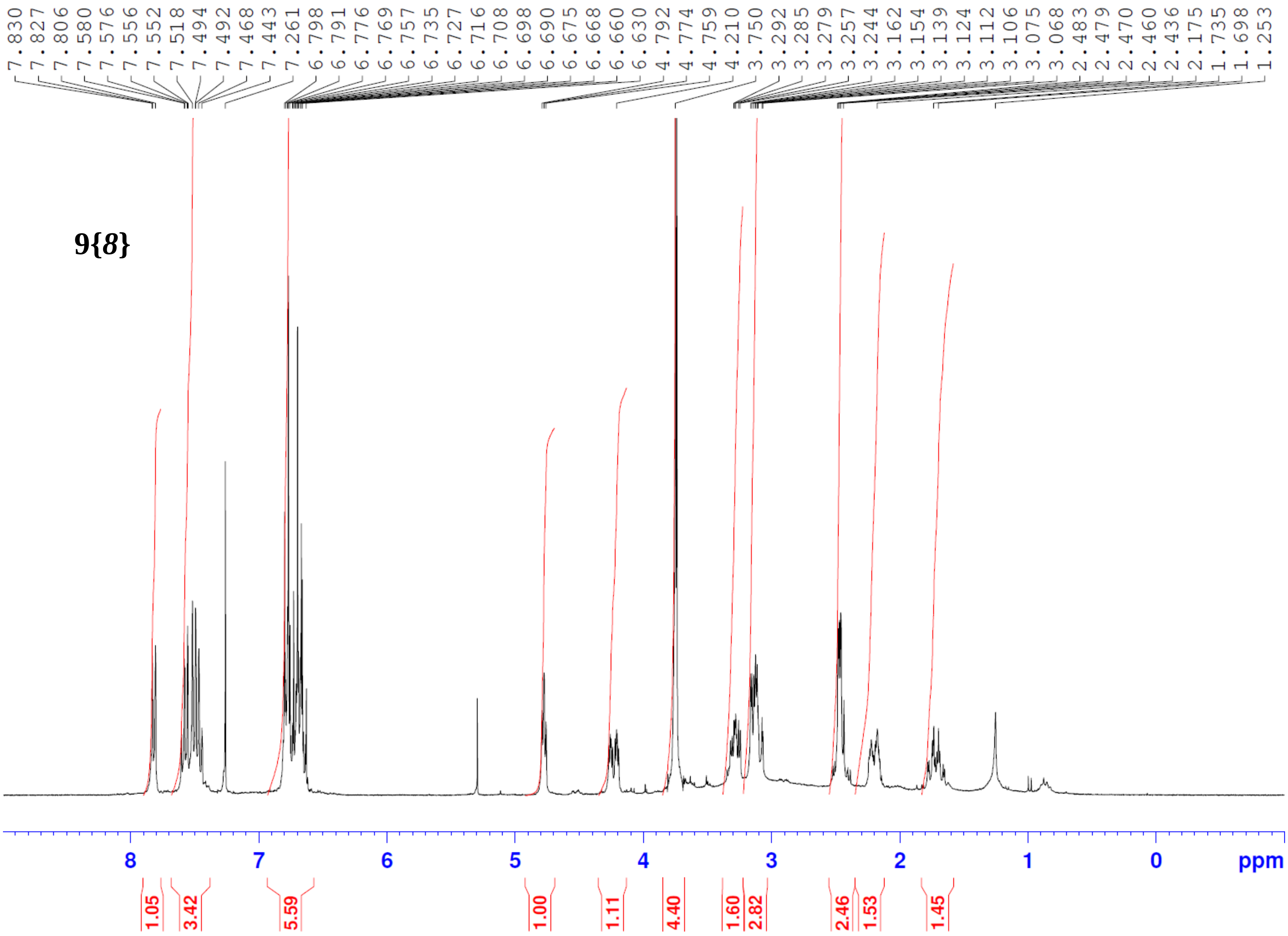

7.830
7.827
7.806
7.580
7.576
7.556
7.552
7.518
7.494
7.492
7.468
7.443
7.261
6.798
6.791
6.776
6.769
6.757
6.735
6.727
6.716
6.708
6.698
6.690
6.675
6.668
6.660
6.630
4.792
4.774
4.759
4.210
3.750
3.292
3.285
3.279
3.257
3.244
3.162
3.154
3.139
3.124
3.112
3.106
3.075
3.068
2.483
2.479
2.470
2.460
2.436
2.175
1.735
1.698
1.253
8
7
6
5
4
3
2
1
0
ppm
1.05
3.42
5.59
1.00
1.11
4.40
1.60
2.82
2.46
1.53
1.45

9{*8*}

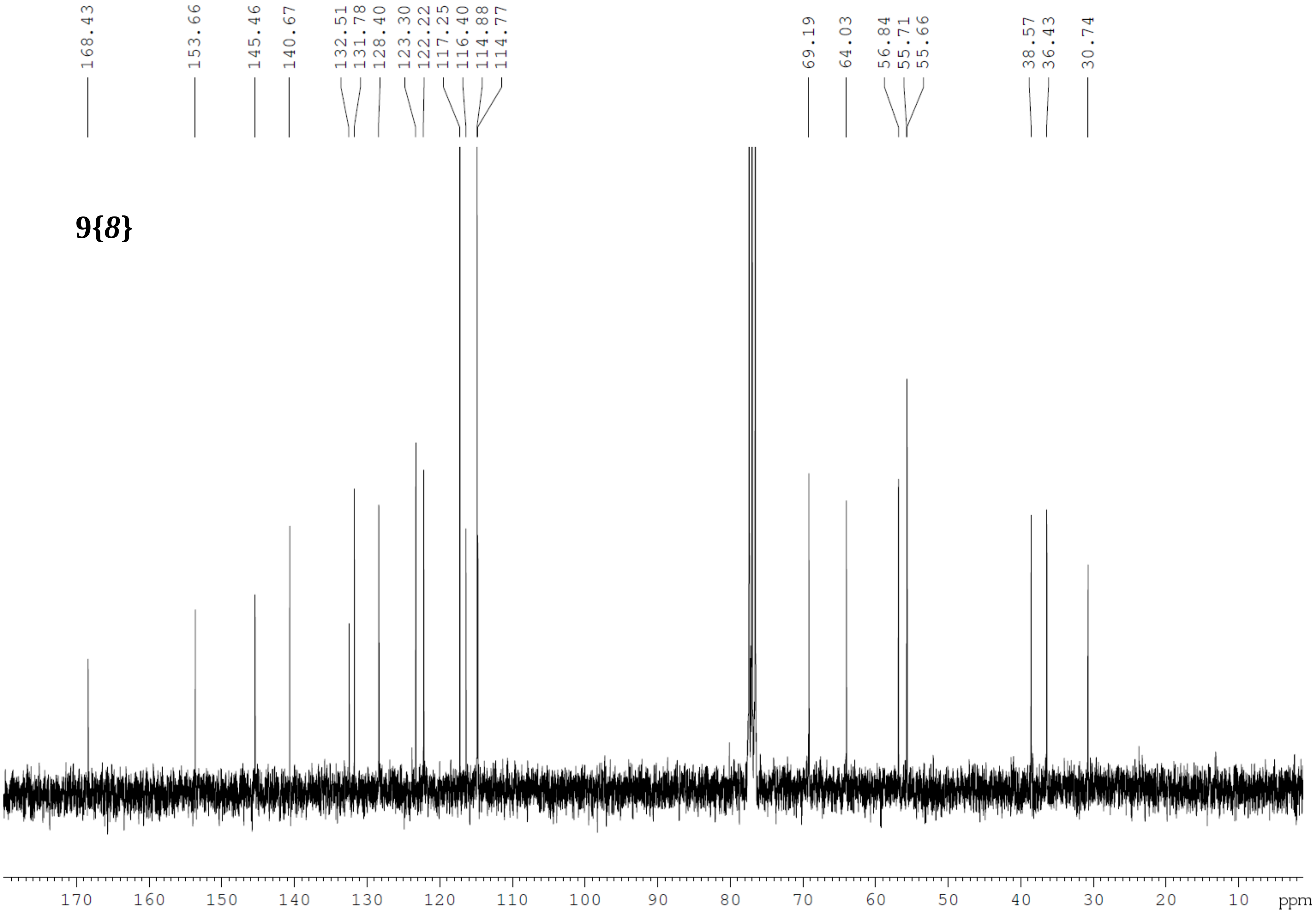

168.43
153.66
145.46
140.67
132.51
131.78
128.40
123.30
122.22
117.25
116.40
114.88
114.77
69.19
64.03
56.84
55.71
55.66
38.57
36.43
30.74
170
160
150
140
130
120
110
100
90
80
70
60
50
40
30
20
10
ppm

10{*8*}

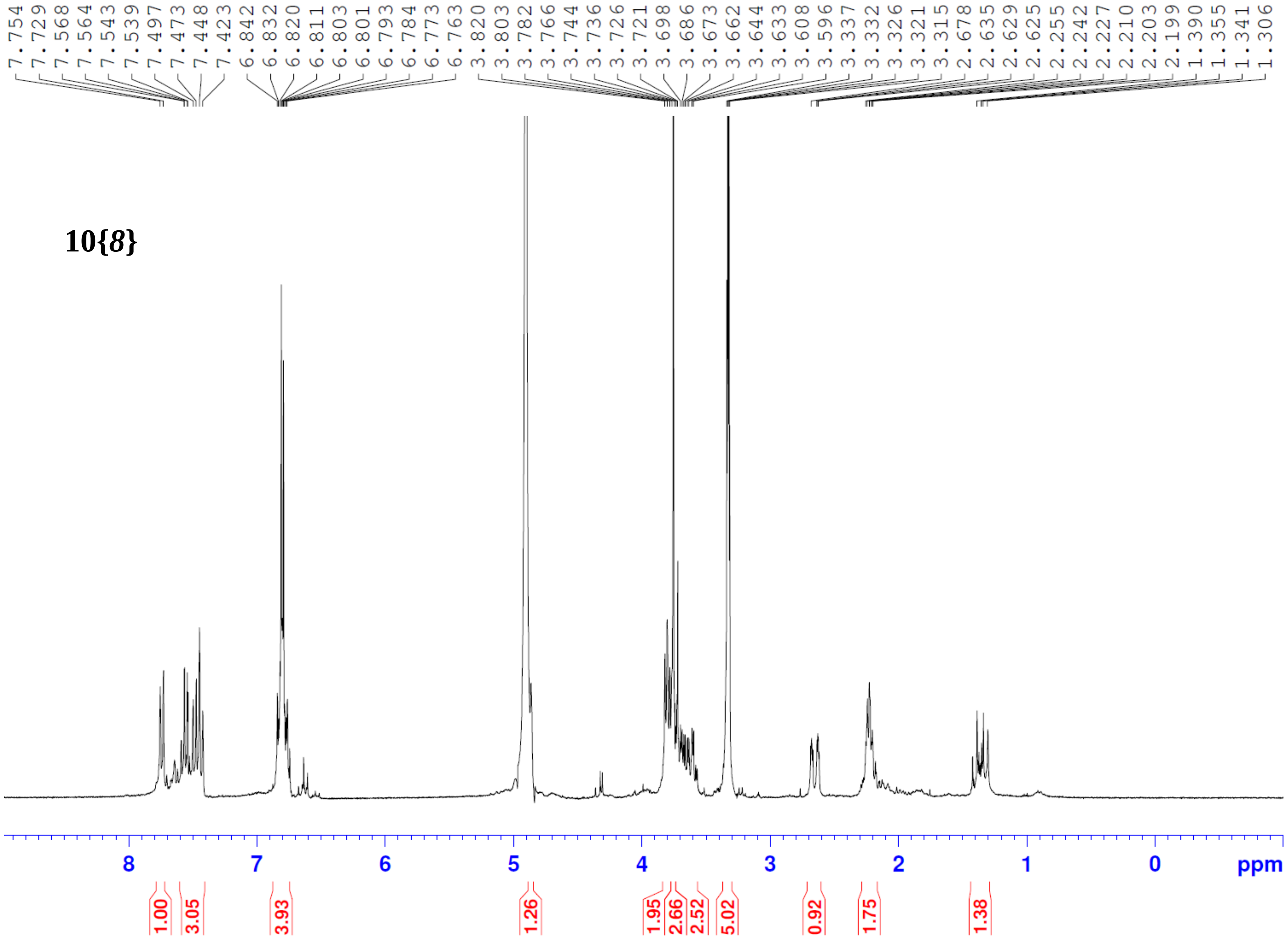

7.754 7.729 7.568 7.564 7.543 7.539 7.497 7.473 7.448 7.423 6.842 6.832 6.820 6.811 6.803 6.801 6.793 6.784 6.773 6.763 3.820 3.803 3.782 3.766 3.744 3.736 3.726 3.721 3.698 3.686 3.673 3.662 3.644 3.633 3.608 3.596 3.337 3.332 3.326 3.321 3.315 2.678 2.635 2.629 2.625 2.255 2.242 2.227 2.210 2.203 2.199 1.390 1.355 1.341 1.306
8 7 6 5 4 3 2 1 0 ppm
1.00 3.05 3.93 1.26 1.95 2.66 2.52 5.02 0.92 1.75 1.38

9{*9*}

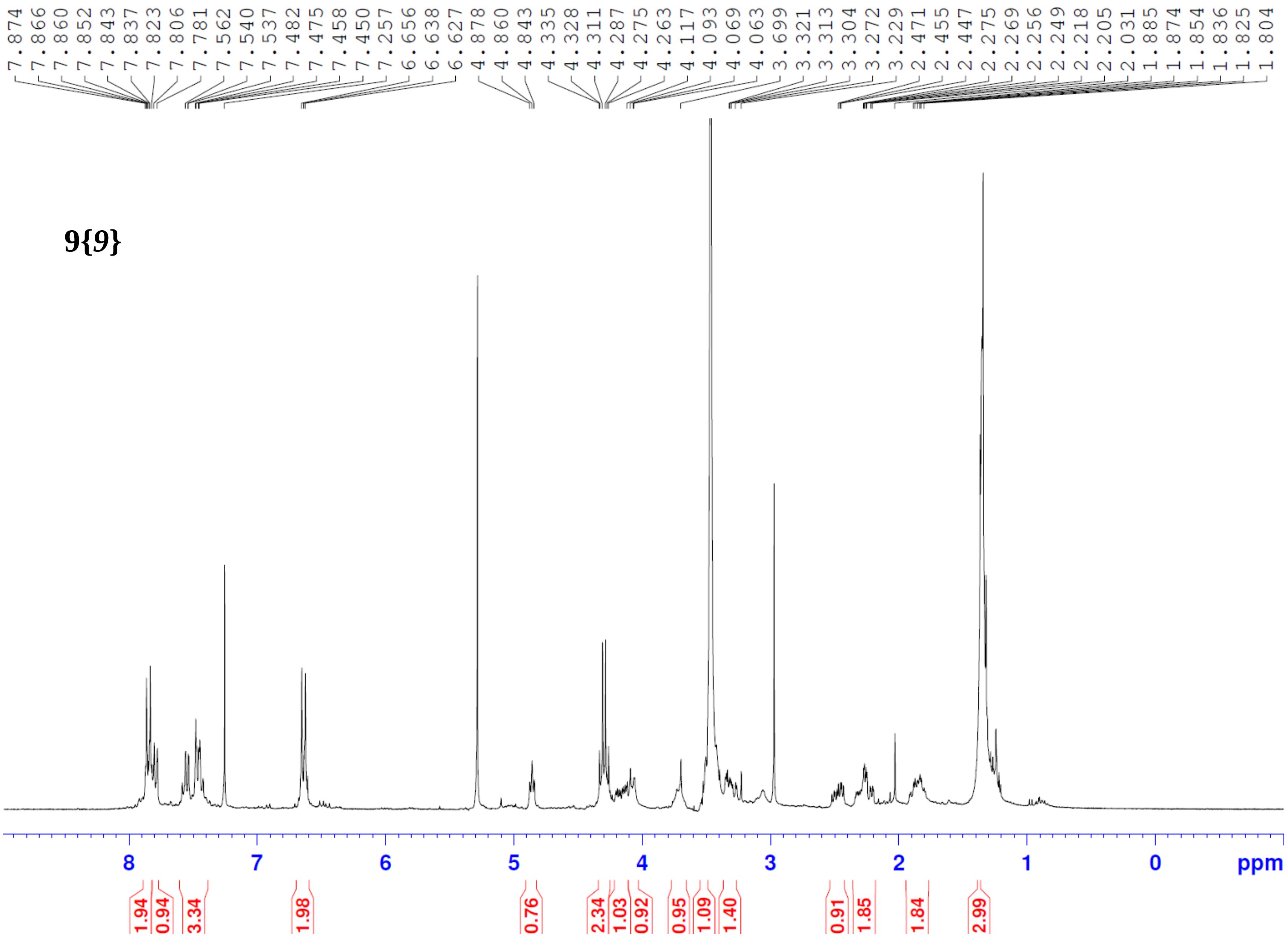

10{*9*}

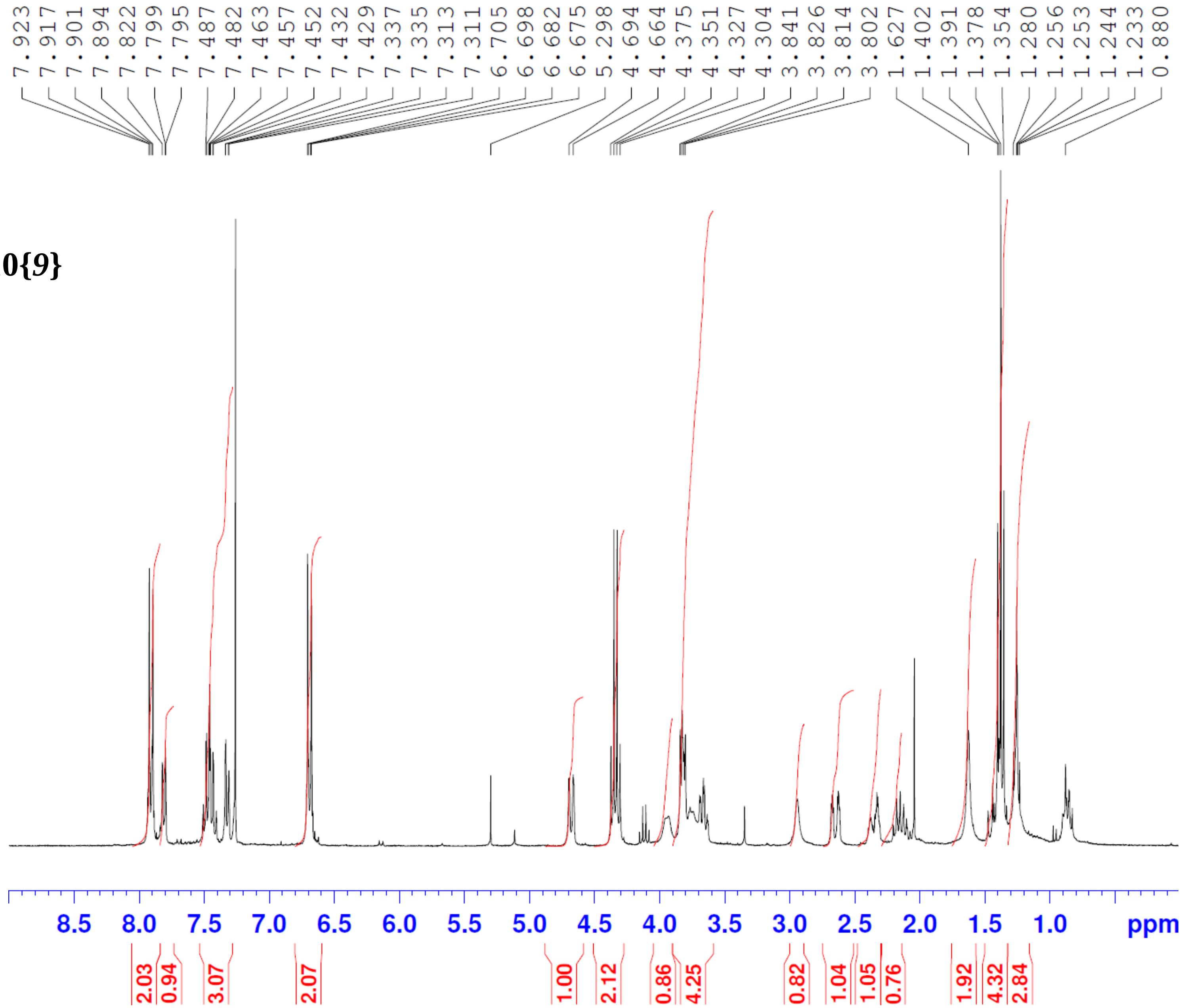
7.923
7.917
7.901
7.894
7.822
7.799
7.795
7.487
7.482
7.463
7.457
7.452
7.432
7.429
7.337
7.335
7.313
7.311
6.705
6.698
6.682
6.675
5.298
4.694
4.664
4.375
4.351
4.327
4.304
3.841
3.826
3.814
3.802
1.627
1.402
1.391
1.378
1.354
1.280
1.256
1.253
1.244
1.233
0.880
8.5
8.0
7.5
7.0
6.5
6.0
5.5
5.0
4.5
4.0
3.5
3.0
2.5
2.0
1.5
1.0
ppm
2.03
0.94
3.07
2.07
1.00
2.12
0.86
4.25
0.82
1.04
1.05
0.76
1.92
4.32
2.84

10{*9*}

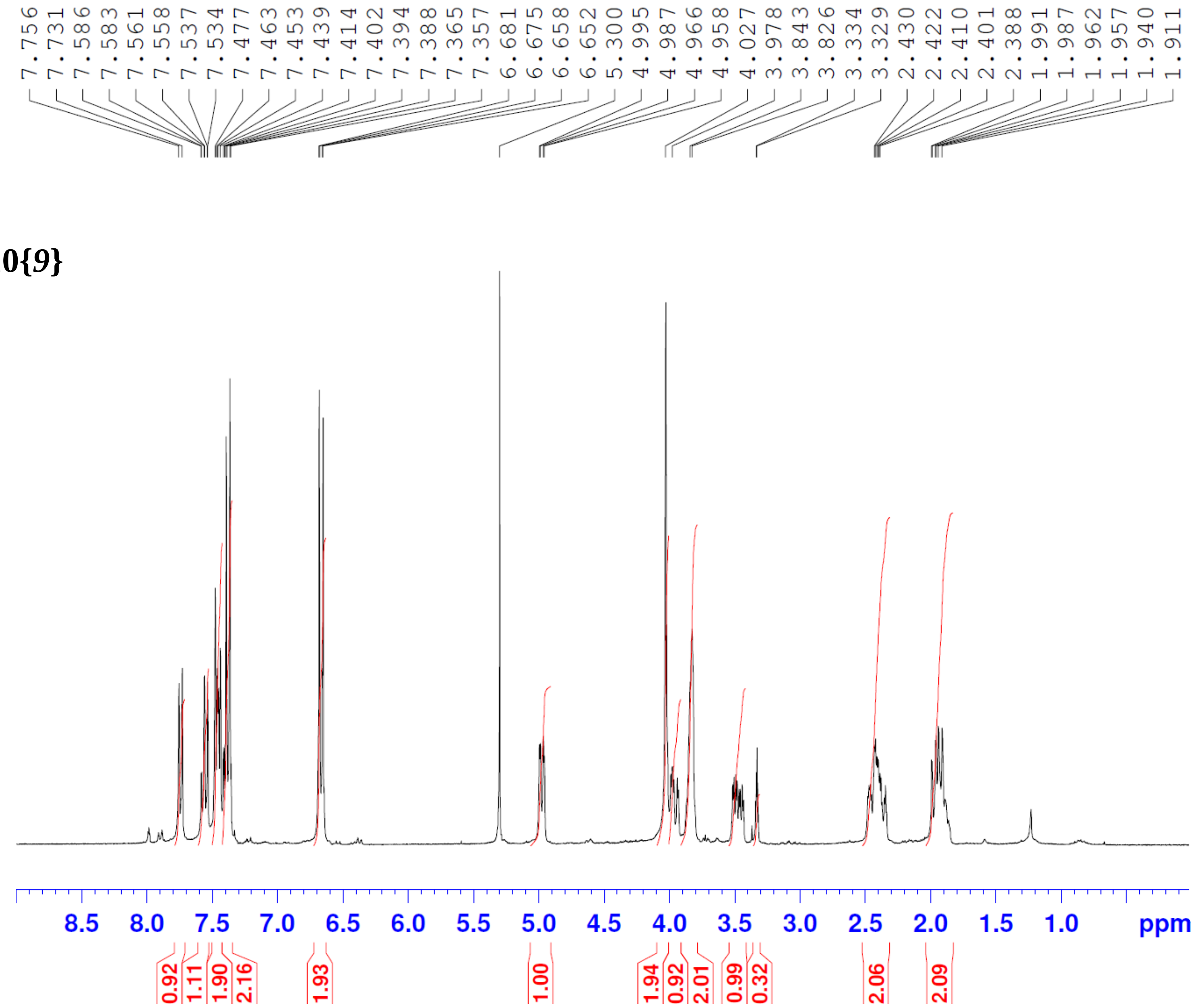

7.756
7.731
7.586
7.583
7.561
7.558
7.537
7.534
7.477
7.463
7.453
7.439
7.414
7.402
7.394
7.388
7.365
7.357
6.681
6.675
6.658
6.652
5.300
4.995
4.987
4.966
4.958
4.027
3.978
3.843
3.826
3.334
3.329
2.430
2.422
2.410
2.401
2.388
1.991
1.987
1.962
1.957
1.940
1.911
8.5
8.0
7.5
7.0
6.5
6.0
5.5
5.0
4.5
4.0
3.5
3.0
2.5
2.0
1.5
1.0
ppm
0.92
1.11
1.90
2.16
1.93
1.00
1.94
0.92
2.01
0.99
0.32
2.06
2.09

10{*10*}

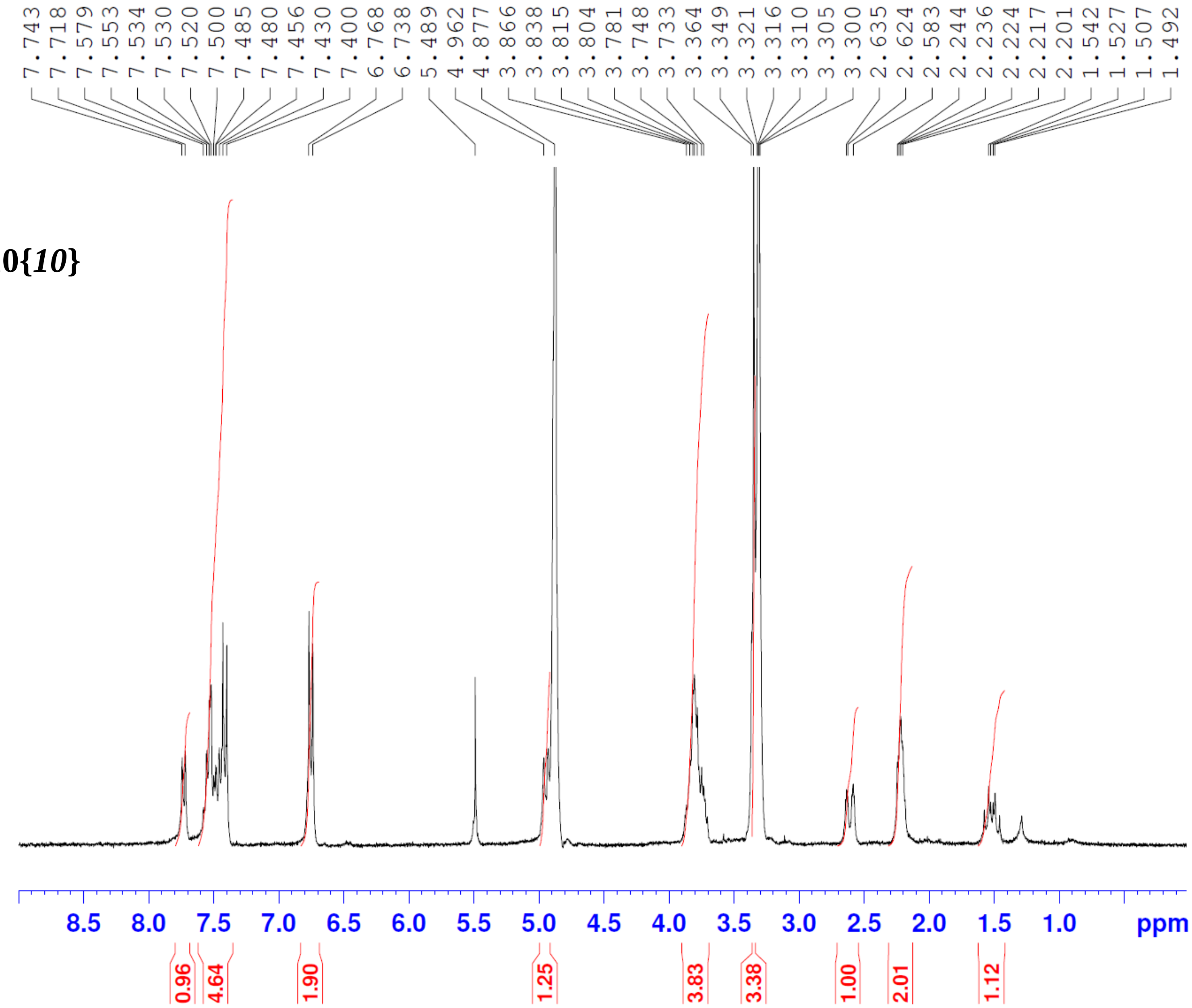
7.743
7.718
7.579
7.553
7.534
7.530
7.520
7.500
7.485
7.480
7.456
7.430
7.400
6.768
6.738
5.489
4.962
4.877
3.866
3.838
3.815
3.804
3.781
3.748
3.733
3.364
3.349
3.321
3.316
3.310
3.305
3.300
2.635
2.624
2.583
2.244
2.236
2.224
2.217
2.201
1.542
1.527
1.507
1.492
8.5 8.0 7.5 7.0 6.5 6.0 5.5 5.0 4.5 4.0 3.5 3.0 2.5 2.0 1.5 1.0 ppm
0.96
4.64
1.90
1.25
3.83
3.38
1.00
2.01
1.12

9{*11*}

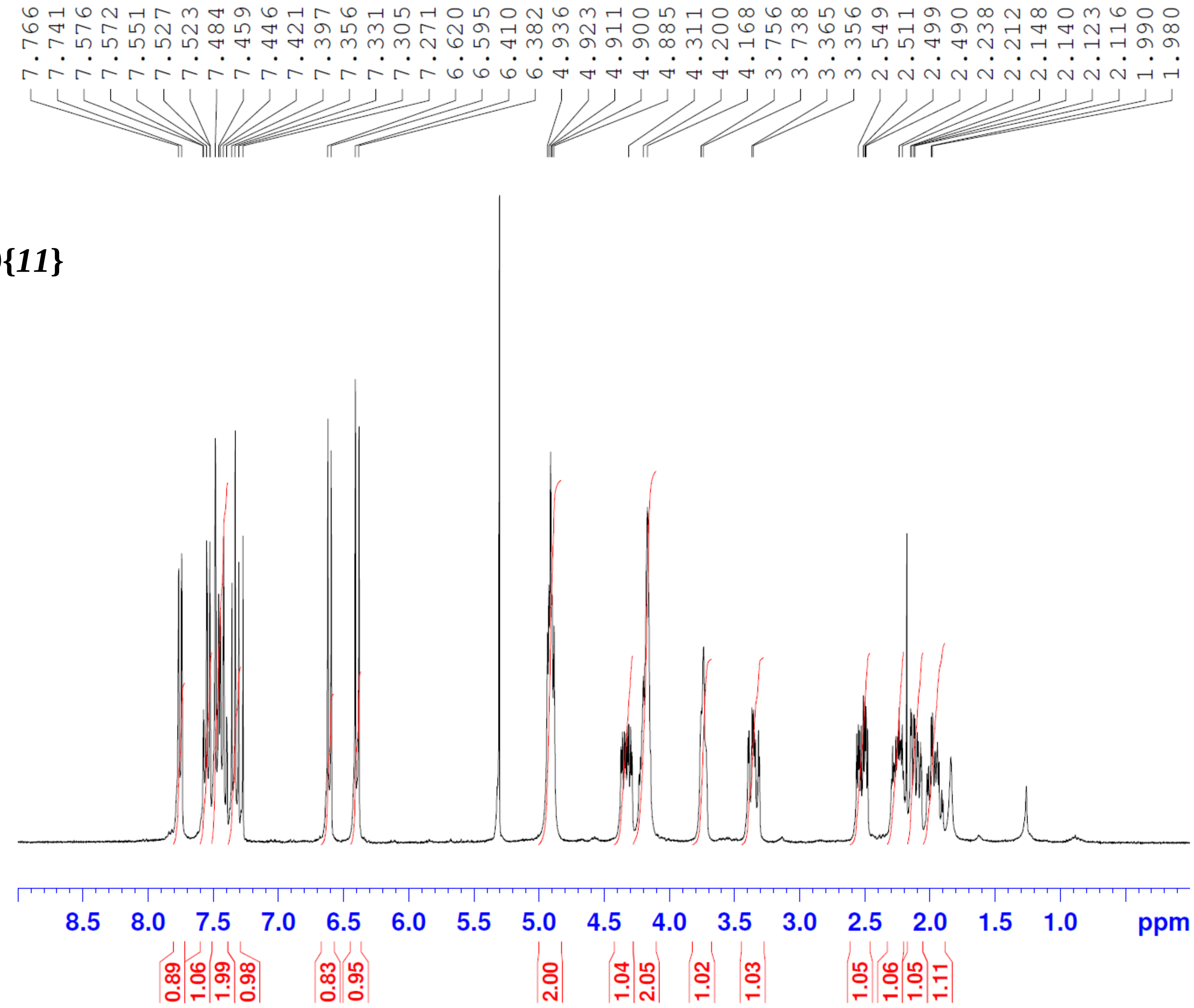
7.766
7.741
7.576
7.572
7.551
7.527
7.523
7.484
7.459
7.446
7.421
7.397
7.356
7.331
7.305
7.271
6.620
6.595
6.410
6.382
4.936
4.923
4.911
4.900
4.885
4.311
4.200
4.168
3.756
3.738
3.365
3.356
2.549
2.511
2.499
2.490
2.238
2.212
2.148
2.140
2.123
2.116
1.990
1.980
8.5
8.0
7.5
7.0
6.5
6.0
5.5
5.0
4.5
4.0
3.5
3.0
2.5
2.0
1.5
1.0
ppm
0.89
1.06
1.99
0.98
0.83
0.95
2.00
1.04
2.05
1.02
1.03
1.05
1.06
1.05
1.11

10{*11*}

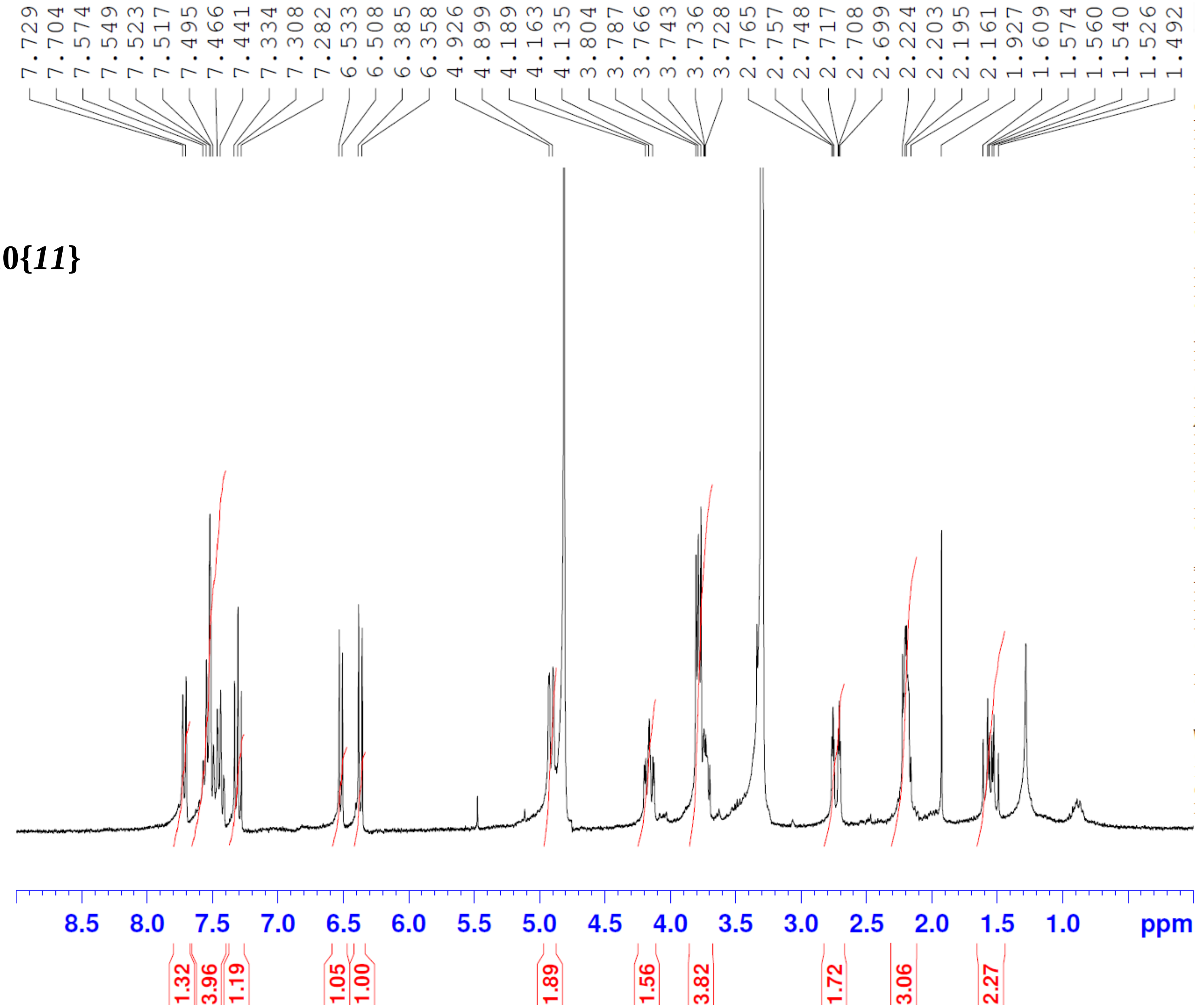
7.729
7.704
7.574
7.549
7.523
7.517
7.495
7.466
7.441
7.334
7.308
7.282
6.533
6.508
6.385
6.358
4.926
4.899
4.189
4.163
4.135
3.804
3.787
3.766
3.743
3.736
3.728
2.765
2.757
2.748
2.717
2.708
2.699
2.224
2.203
2.195
2.161
1.927
1.609
1.574
1.560
1.540
1.526
1.492
8.5
8.0
7.5
7.0
6.5
6.0
5.5
5.0
4.5
4.0
3.5
3.0
2.5
2.0
1.5
1.0
ppm
1.32
3.96
1.19
1.05
1.00
1.89
1.56
3.82
1.72
3.06
2.27

9{*12*}

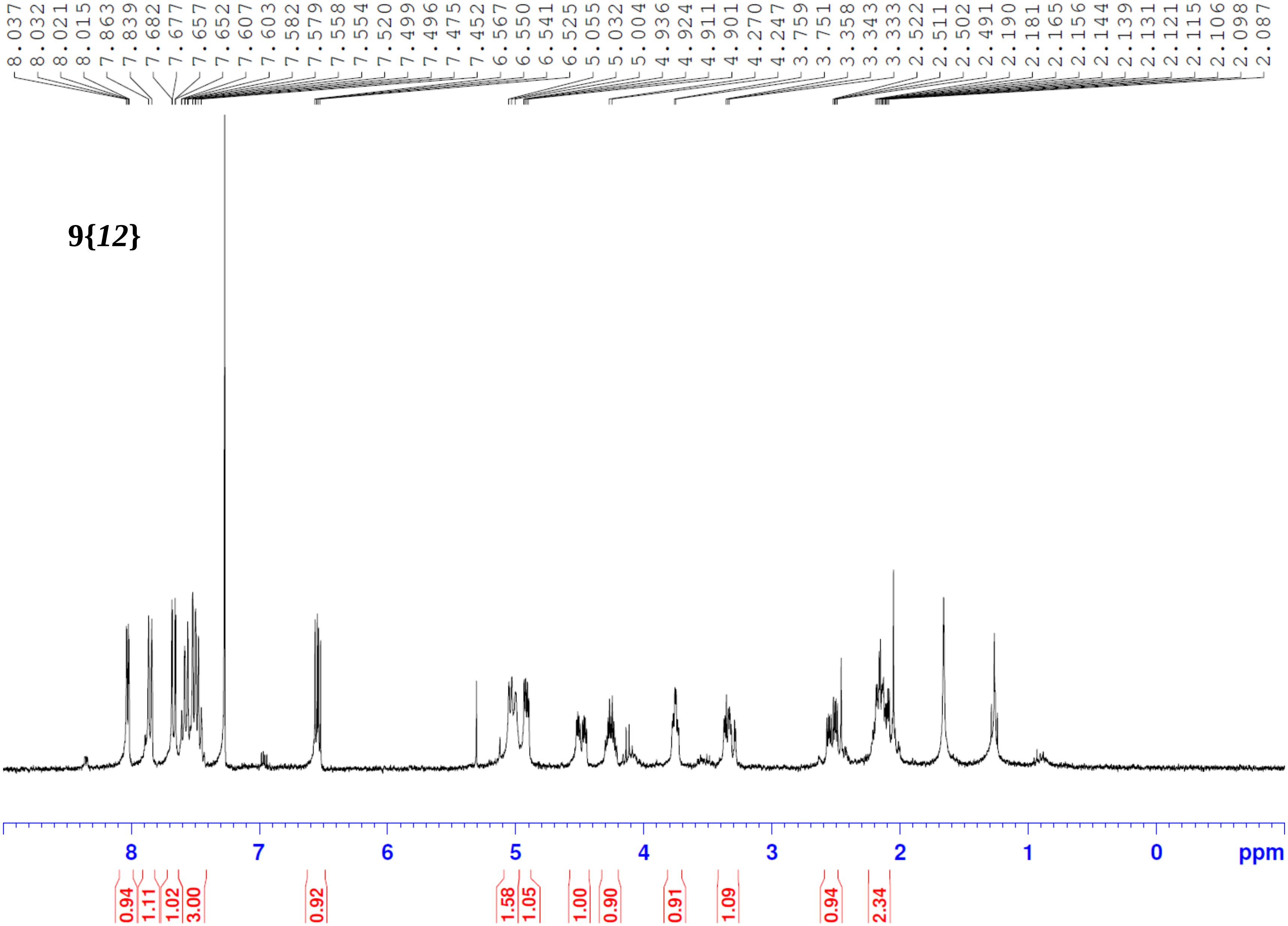
8.037
8.032
8.021
8.015
7.863
7.839
7.682
7.677
7.657
7.652
7.607
7.603
7.582
7.579
7.558
7.554
7.520
7.499
7.496
7.475
7.452
6.567
6.550
6.541
6.525
5.055
5.032
5.004
4.936
4.924
4.911
4.901
4.270
4.247
3.759
3.751
3.358
3.343
3.333
2.522
2.511
2.502
2.491
2.190
2.181
2.165
2.156
2.144
2.139
2.131
2.121
2.115
2.106
2.098
2.087
8
7
6
5
4
3
2
1
0
ppm
0.94
1.11
1.02
3.00
0.92
1.58
1.05
1.00
0.90
0.91
1.09
0.94
2.34

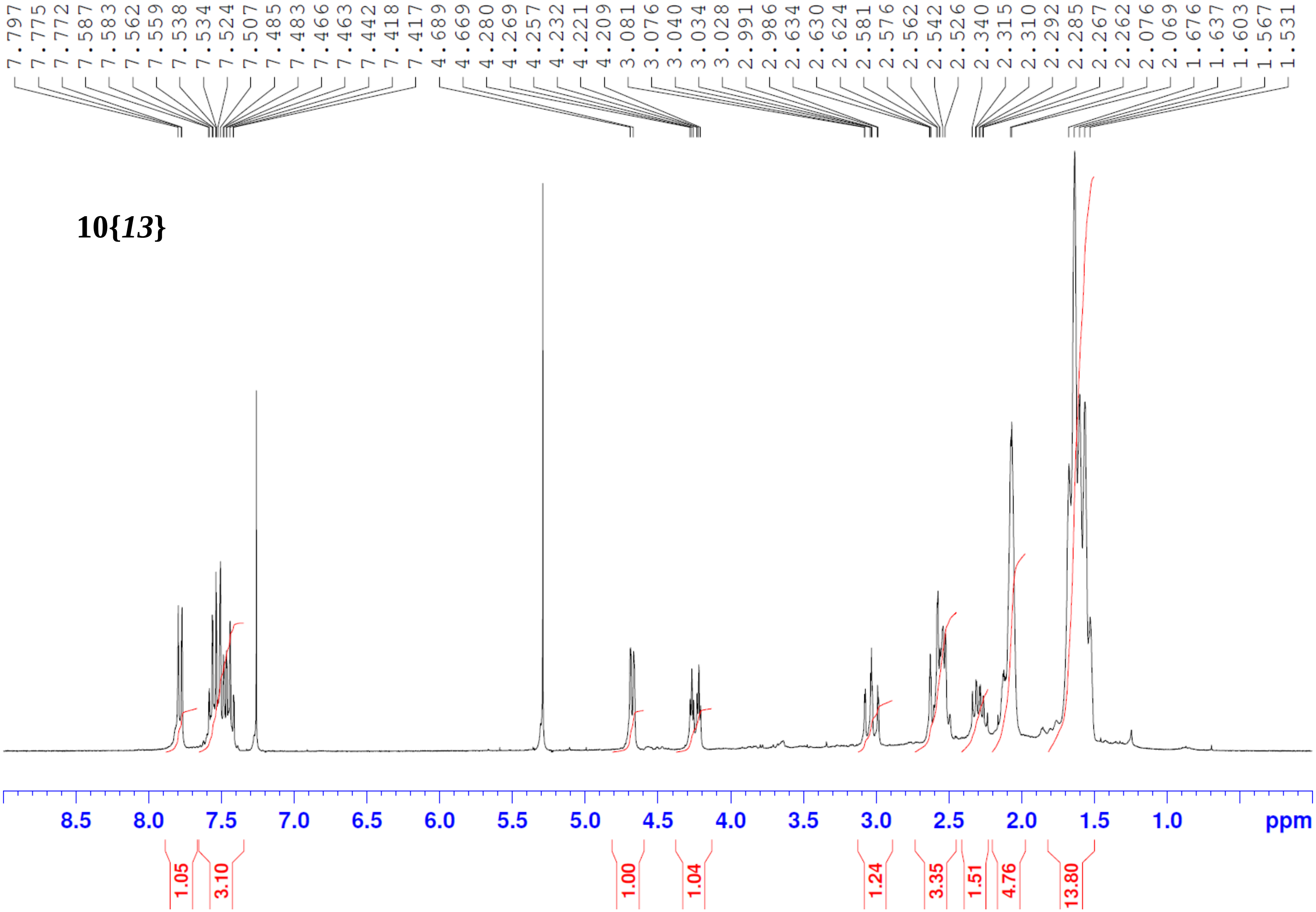
10{13}
7.797
7.775
7.772
7.587
7.583
7.562
7.559
7.538
7.534
7.524
7.507
7.485
7.483
7.466
7.463
7.442
7.418
7.417
4.689
4.669
4.280
4.269
4.257
4.232
4.221
4.209
3.081
3.076
3.040
3.034
3.028
2.991
2.986
2.634
2.630
2.624
2.581
2.576
2.562
2.542
2.526
2.340
2.315
2.310
2.292
2.285
2.267
2.262
2.076
2.069
1.676
1.637
1.603
1.567
1.531
8.5
8.0
7.5
7.0
6.5
6.0
5.5
5.0
4.5
4.0
3.5
3.0
2.5
2.0
1.5
1.0
ppm
1.05
3.10
1.00
1.04
1.24
3.35
1.51
4.76
13.80
